# Supplementary figures and images for: Lateralized cerebellar connectivity differentiates auditory pathways in echolocating and non-echolocating whales
Source: PLoS One. 2025 Jun 6;20(6):e0323617. doi: 10.1371/journal.pone.0323617 (PMC12143552; doi:10.1371/journal.pone.0323617)

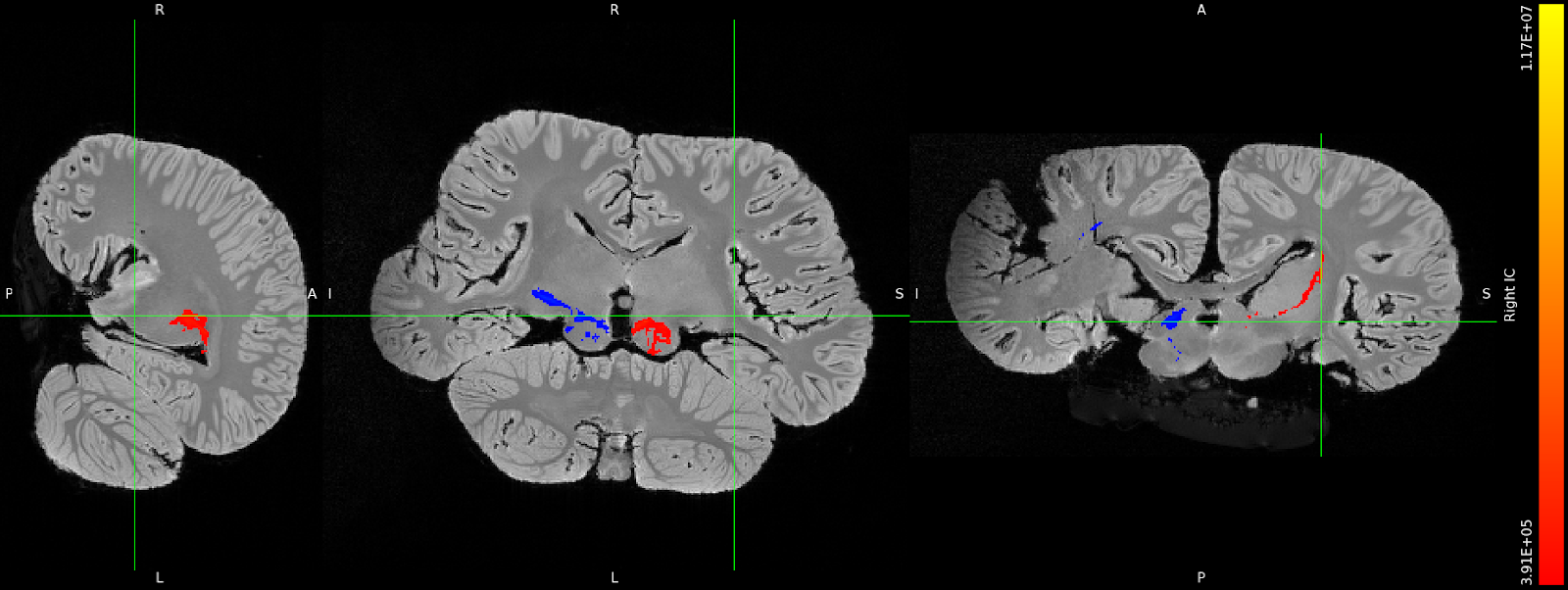

Supplement: S1 File — S1 Text. Detailed cerebellar and subcortical projection sites in IC-cerebellar traces. S2 Text. Detailed cortical projections in IC-cerebellar traces. S3 Figures. Masked regions of interest in FSLeyes. S3 Figure A. D. delphis. Red= right, blue=left for cerebella, yellow=right and turquoise=left for inferior colliculi. S3 Figure B. S. Attenuata. Red= right, blue=left for cerebella, yellow=right and turquoise=left for inferior colliculi. S3 Figure C. L. acutus. Red= right, blue=left for cerebella, yellow=right and turquoise=left for inferior colliculi. S3 Figure D. B. borealis. Red= right, blue=left for cerebella, yellow=right and turquoise=left for inferior colliculi. S4 Figures. Ascending auditory tractograms. S4 Figure A1: D. delphis, left IC tracts shown in blue, right IC tracts shown in red, minimum threshold set to 1% and maximum threshold set to 30% of waytotals. Orthographic view. S4 Figure A2: D. delphis, left IC tracts shown in blue, right IC tracts shown in red, set to a more liberal threshold of minimum 0.1% and maximum 5% of waytotals. Orthographic view. S4 Figure A3: D. delphis, left IC tracts shown in blue, right IC tracts shown in red, set to a more liberal threshold of minimum 0.1% and maximum 5% of waytotals. Still 3-dimensional view. S4 Figure A4: D. delphis, left IC tracts shown in blue, right IC tracts shown in red, set to a more liberal threshold of minimum 0.1% and maximum 5% of waytotals. Rotating 3-dimensional view. S4 Figure B1: S. attenuata, left IC tracts shown in blue, right IC tracts shown in red, minimum threshold set to 1% and maximum threshold set to 30% of waytotals. Orthographic view. S4 Figure B2: S. attenuata, left IC tracts shown in blue, right IC tracts shown in red, set to a more liberal threshold of minimum 0.1% and maximum 5% of waytotals. Orthographic view. S4 Figure B3: S. attenuata, left IC tracts shown in blue, right IC tracts shown in red, set to a more liberal threshold of minimum 0.1% and maximum 5% of waytotals. Still [file pone.0323617.s001.zip › supporting_information/s4_fig_d1.png]

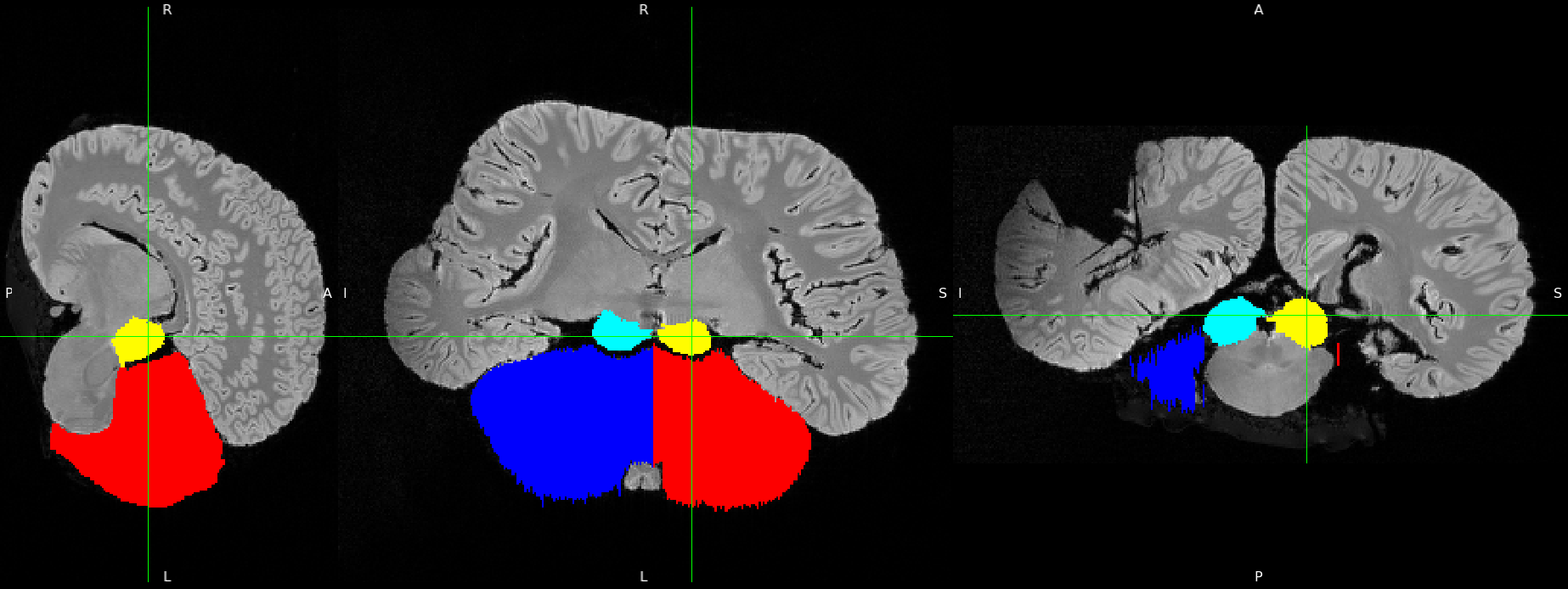

Supplement: S1 File — S1 Text. Detailed cerebellar and subcortical projection sites in IC-cerebellar traces. S2 Text. Detailed cortical projections in IC-cerebellar traces. S3 Figures. Masked regions of interest in FSLeyes. S3 Figure A. D. delphis. Red= right, blue=left for cerebella, yellow=right and turquoise=left for inferior colliculi. S3 Figure B. S. Attenuata. Red= right, blue=left for cerebella, yellow=right and turquoise=left for inferior colliculi. S3 Figure C. L. acutus. Red= right, blue=left for cerebella, yellow=right and turquoise=left for inferior colliculi. S3 Figure D. B. borealis. Red= right, blue=left for cerebella, yellow=right and turquoise=left for inferior colliculi. S4 Figures. Ascending auditory tractograms. S4 Figure A1: D. delphis, left IC tracts shown in blue, right IC tracts shown in red, minimum threshold set to 1% and maximum threshold set to 30% of waytotals. Orthographic view. S4 Figure A2: D. delphis, left IC tracts shown in blue, right IC tracts shown in red, set to a more liberal threshold of minimum 0.1% and maximum 5% of waytotals. Orthographic view. S4 Figure A3: D. delphis, left IC tracts shown in blue, right IC tracts shown in red, set to a more liberal threshold of minimum 0.1% and maximum 5% of waytotals. Still 3-dimensional view. S4 Figure A4: D. delphis, left IC tracts shown in blue, right IC tracts shown in red, set to a more liberal threshold of minimum 0.1% and maximum 5% of waytotals. Rotating 3-dimensional view. S4 Figure B1: S. attenuata, left IC tracts shown in blue, right IC tracts shown in red, minimum threshold set to 1% and maximum threshold set to 30% of waytotals. Orthographic view. S4 Figure B2: S. attenuata, left IC tracts shown in blue, right IC tracts shown in red, set to a more liberal threshold of minimum 0.1% and maximum 5% of waytotals. Orthographic view. S4 Figure B3: S. attenuata, left IC tracts shown in blue, right IC tracts shown in red, set to a more liberal threshold of minimum 0.1% and maximum 5% of waytotals. Still [file pone.0323617.s001.zip › supporting_information/s3_fig_d.png]

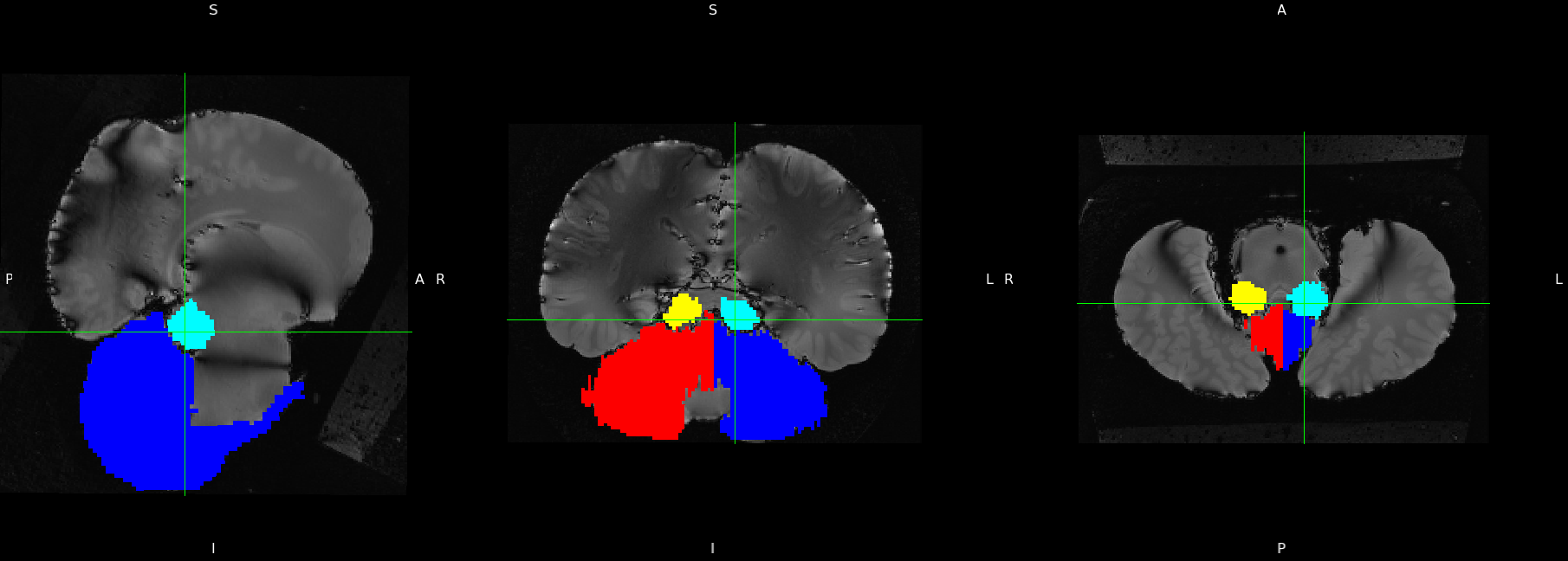

Supplement: S1 File — S1 Text. Detailed cerebellar and subcortical projection sites in IC-cerebellar traces. S2 Text. Detailed cortical projections in IC-cerebellar traces. S3 Figures. Masked regions of interest in FSLeyes. S3 Figure A. D. delphis. Red= right, blue=left for cerebella, yellow=right and turquoise=left for inferior colliculi. S3 Figure B. S. Attenuata. Red= right, blue=left for cerebella, yellow=right and turquoise=left for inferior colliculi. S3 Figure C. L. acutus. Red= right, blue=left for cerebella, yellow=right and turquoise=left for inferior colliculi. S3 Figure D. B. borealis. Red= right, blue=left for cerebella, yellow=right and turquoise=left for inferior colliculi. S4 Figures. Ascending auditory tractograms. S4 Figure A1: D. delphis, left IC tracts shown in blue, right IC tracts shown in red, minimum threshold set to 1% and maximum threshold set to 30% of waytotals. Orthographic view. S4 Figure A2: D. delphis, left IC tracts shown in blue, right IC tracts shown in red, set to a more liberal threshold of minimum 0.1% and maximum 5% of waytotals. Orthographic view. S4 Figure A3: D. delphis, left IC tracts shown in blue, right IC tracts shown in red, set to a more liberal threshold of minimum 0.1% and maximum 5% of waytotals. Still 3-dimensional view. S4 Figure A4: D. delphis, left IC tracts shown in blue, right IC tracts shown in red, set to a more liberal threshold of minimum 0.1% and maximum 5% of waytotals. Rotating 3-dimensional view. S4 Figure B1: S. attenuata, left IC tracts shown in blue, right IC tracts shown in red, minimum threshold set to 1% and maximum threshold set to 30% of waytotals. Orthographic view. S4 Figure B2: S. attenuata, left IC tracts shown in blue, right IC tracts shown in red, set to a more liberal threshold of minimum 0.1% and maximum 5% of waytotals. Orthographic view. S4 Figure B3: S. attenuata, left IC tracts shown in blue, right IC tracts shown in red, set to a more liberal threshold of minimum 0.1% and maximum 5% of waytotals. Still [file pone.0323617.s001.zip › supporting_information/s3_fig_b.png]

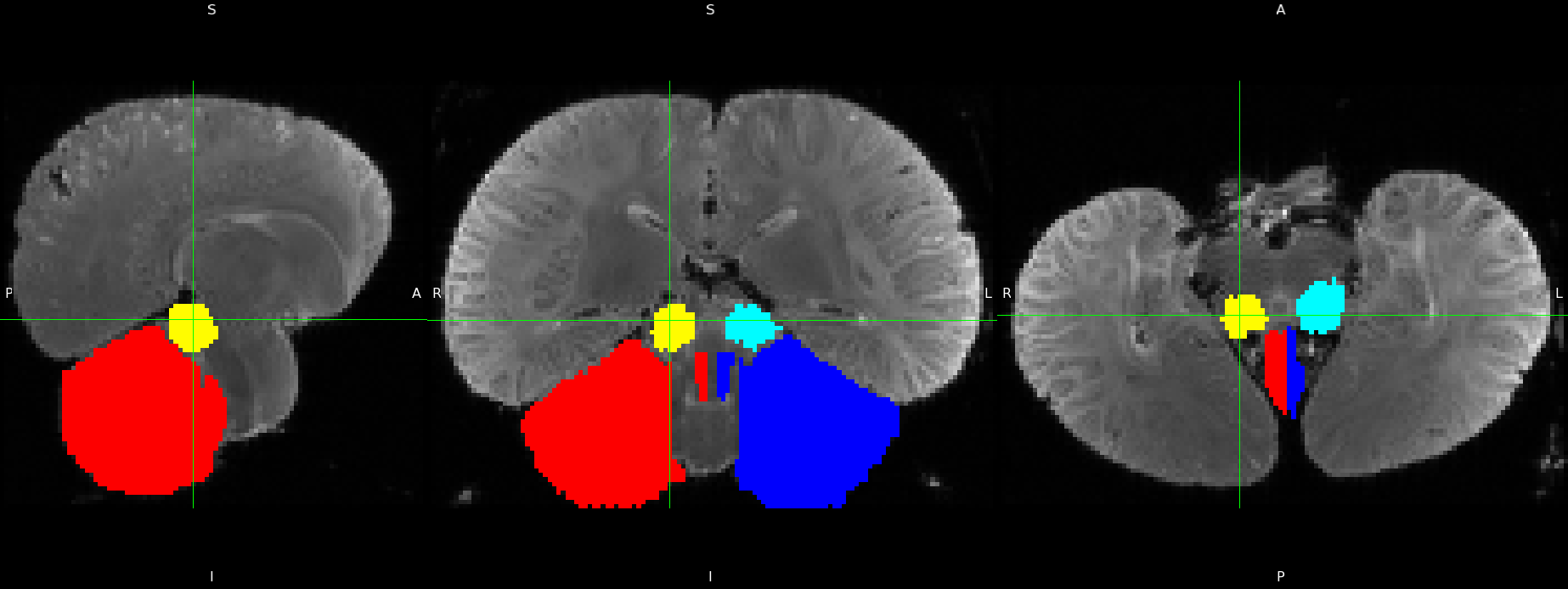

Supplement: S1 File — S1 Text. Detailed cerebellar and subcortical projection sites in IC-cerebellar traces. S2 Text. Detailed cortical projections in IC-cerebellar traces. S3 Figures. Masked regions of interest in FSLeyes. S3 Figure A. D. delphis. Red= right, blue=left for cerebella, yellow=right and turquoise=left for inferior colliculi. S3 Figure B. S. Attenuata. Red= right, blue=left for cerebella, yellow=right and turquoise=left for inferior colliculi. S3 Figure C. L. acutus. Red= right, blue=left for cerebella, yellow=right and turquoise=left for inferior colliculi. S3 Figure D. B. borealis. Red= right, blue=left for cerebella, yellow=right and turquoise=left for inferior colliculi. S4 Figures. Ascending auditory tractograms. S4 Figure A1: D. delphis, left IC tracts shown in blue, right IC tracts shown in red, minimum threshold set to 1% and maximum threshold set to 30% of waytotals. Orthographic view. S4 Figure A2: D. delphis, left IC tracts shown in blue, right IC tracts shown in red, set to a more liberal threshold of minimum 0.1% and maximum 5% of waytotals. Orthographic view. S4 Figure A3: D. delphis, left IC tracts shown in blue, right IC tracts shown in red, set to a more liberal threshold of minimum 0.1% and maximum 5% of waytotals. Still 3-dimensional view. S4 Figure A4: D. delphis, left IC tracts shown in blue, right IC tracts shown in red, set to a more liberal threshold of minimum 0.1% and maximum 5% of waytotals. Rotating 3-dimensional view. S4 Figure B1: S. attenuata, left IC tracts shown in blue, right IC tracts shown in red, minimum threshold set to 1% and maximum threshold set to 30% of waytotals. Orthographic view. S4 Figure B2: S. attenuata, left IC tracts shown in blue, right IC tracts shown in red, set to a more liberal threshold of minimum 0.1% and maximum 5% of waytotals. Orthographic view. S4 Figure B3: S. attenuata, left IC tracts shown in blue, right IC tracts shown in red, set to a more liberal threshold of minimum 0.1% and maximum 5% of waytotals. Still [file pone.0323617.s001.zip › supporting_information/s3_fig_a.png]

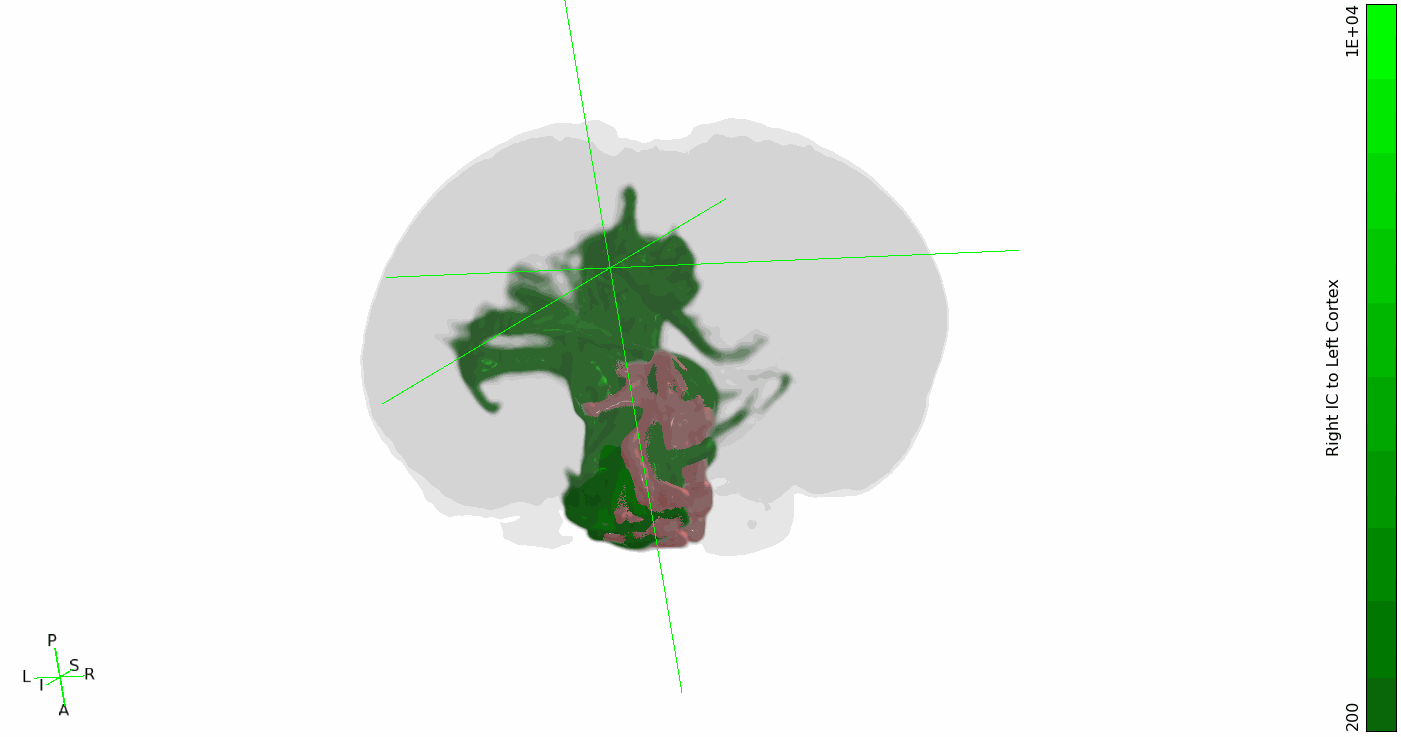

Supplement: S1 File — S1 Text. Detailed cerebellar and subcortical projection sites in IC-cerebellar traces. S2 Text. Detailed cortical projections in IC-cerebellar traces. S3 Figures. Masked regions of interest in FSLeyes. S3 Figure A. D. delphis. Red= right, blue=left for cerebella, yellow=right and turquoise=left for inferior colliculi. S3 Figure B. S. Attenuata. Red= right, blue=left for cerebella, yellow=right and turquoise=left for inferior colliculi. S3 Figure C. L. acutus. Red= right, blue=left for cerebella, yellow=right and turquoise=left for inferior colliculi. S3 Figure D. B. borealis. Red= right, blue=left for cerebella, yellow=right and turquoise=left for inferior colliculi. S4 Figures. Ascending auditory tractograms. S4 Figure A1: D. delphis, left IC tracts shown in blue, right IC tracts shown in red, minimum threshold set to 1% and maximum threshold set to 30% of waytotals. Orthographic view. S4 Figure A2: D. delphis, left IC tracts shown in blue, right IC tracts shown in red, set to a more liberal threshold of minimum 0.1% and maximum 5% of waytotals. Orthographic view. S4 Figure A3: D. delphis, left IC tracts shown in blue, right IC tracts shown in red, set to a more liberal threshold of minimum 0.1% and maximum 5% of waytotals. Still 3-dimensional view. S4 Figure A4: D. delphis, left IC tracts shown in blue, right IC tracts shown in red, set to a more liberal threshold of minimum 0.1% and maximum 5% of waytotals. Rotating 3-dimensional view. S4 Figure B1: S. attenuata, left IC tracts shown in blue, right IC tracts shown in red, minimum threshold set to 1% and maximum threshold set to 30% of waytotals. Orthographic view. S4 Figure B2: S. attenuata, left IC tracts shown in blue, right IC tracts shown in red, set to a more liberal threshold of minimum 0.1% and maximum 5% of waytotals. Orthographic view. S4 Figure B3: S. attenuata, left IC tracts shown in blue, right IC tracts shown in red, set to a more liberal threshold of minimum 0.1% and maximum 5% of waytotals. Still [file pone.0323617.s001.zip › supporting_information/s5_fig_a4.gif]

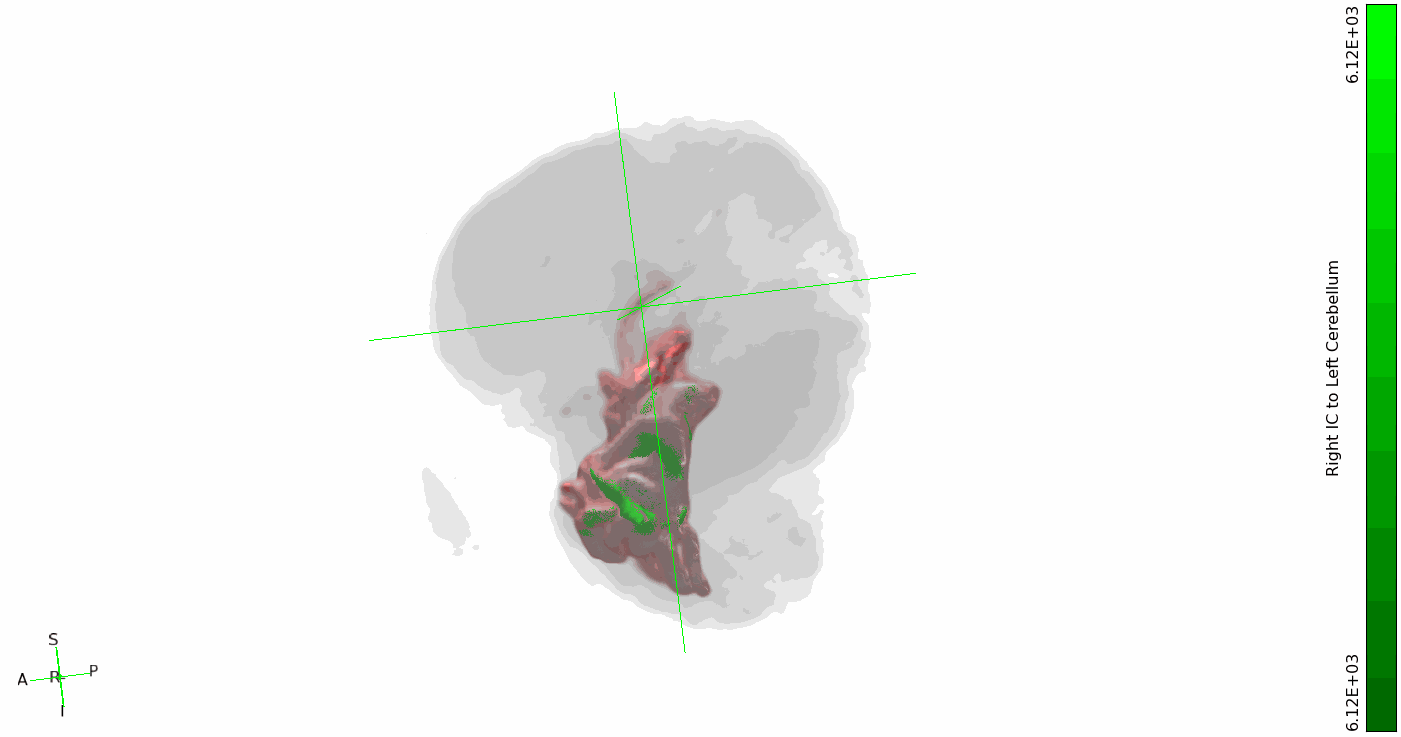

Supplement: S1 File — S1 Text. Detailed cerebellar and subcortical projection sites in IC-cerebellar traces. S2 Text. Detailed cortical projections in IC-cerebellar traces. S3 Figures. Masked regions of interest in FSLeyes. S3 Figure A. D. delphis. Red= right, blue=left for cerebella, yellow=right and turquoise=left for inferior colliculi. S3 Figure B. S. Attenuata. Red= right, blue=left for cerebella, yellow=right and turquoise=left for inferior colliculi. S3 Figure C. L. acutus. Red= right, blue=left for cerebella, yellow=right and turquoise=left for inferior colliculi. S3 Figure D. B. borealis. Red= right, blue=left for cerebella, yellow=right and turquoise=left for inferior colliculi. S4 Figures. Ascending auditory tractograms. S4 Figure A1: D. delphis, left IC tracts shown in blue, right IC tracts shown in red, minimum threshold set to 1% and maximum threshold set to 30% of waytotals. Orthographic view. S4 Figure A2: D. delphis, left IC tracts shown in blue, right IC tracts shown in red, set to a more liberal threshold of minimum 0.1% and maximum 5% of waytotals. Orthographic view. S4 Figure A3: D. delphis, left IC tracts shown in blue, right IC tracts shown in red, set to a more liberal threshold of minimum 0.1% and maximum 5% of waytotals. Still 3-dimensional view. S4 Figure A4: D. delphis, left IC tracts shown in blue, right IC tracts shown in red, set to a more liberal threshold of minimum 0.1% and maximum 5% of waytotals. Rotating 3-dimensional view. S4 Figure B1: S. attenuata, left IC tracts shown in blue, right IC tracts shown in red, minimum threshold set to 1% and maximum threshold set to 30% of waytotals. Orthographic view. S4 Figure B2: S. attenuata, left IC tracts shown in blue, right IC tracts shown in red, set to a more liberal threshold of minimum 0.1% and maximum 5% of waytotals. Orthographic view. S4 Figure B3: S. attenuata, left IC tracts shown in blue, right IC tracts shown in red, set to a more liberal threshold of minimum 0.1% and maximum 5% of waytotals. Still [file pone.0323617.s001.zip › supporting_information/s5_fig_b4.gif]

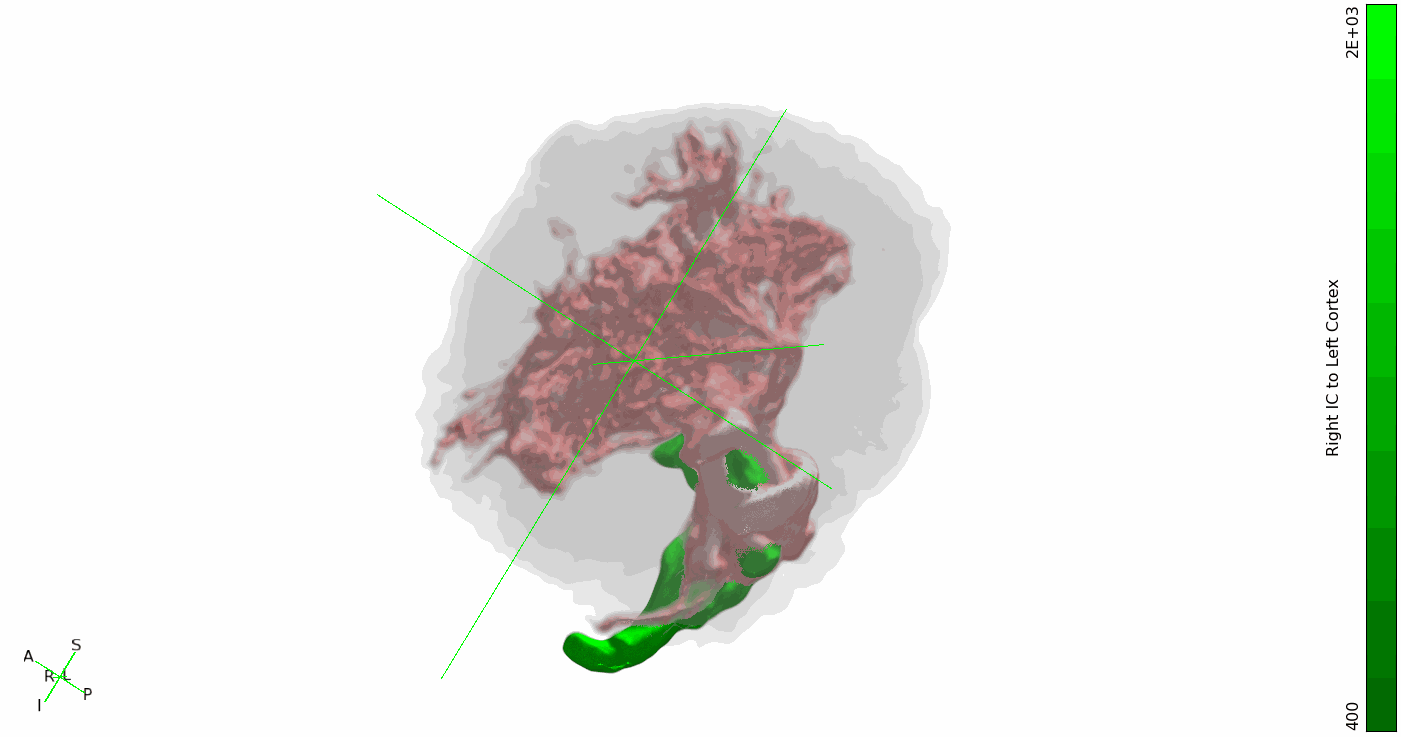

Supplement: S1 File — S1 Text. Detailed cerebellar and subcortical projection sites in IC-cerebellar traces. S2 Text. Detailed cortical projections in IC-cerebellar traces. S3 Figures. Masked regions of interest in FSLeyes. S3 Figure A. D. delphis. Red= right, blue=left for cerebella, yellow=right and turquoise=left for inferior colliculi. S3 Figure B. S. Attenuata. Red= right, blue=left for cerebella, yellow=right and turquoise=left for inferior colliculi. S3 Figure C. L. acutus. Red= right, blue=left for cerebella, yellow=right and turquoise=left for inferior colliculi. S3 Figure D. B. borealis. Red= right, blue=left for cerebella, yellow=right and turquoise=left for inferior colliculi. S4 Figures. Ascending auditory tractograms. S4 Figure A1: D. delphis, left IC tracts shown in blue, right IC tracts shown in red, minimum threshold set to 1% and maximum threshold set to 30% of waytotals. Orthographic view. S4 Figure A2: D. delphis, left IC tracts shown in blue, right IC tracts shown in red, set to a more liberal threshold of minimum 0.1% and maximum 5% of waytotals. Orthographic view. S4 Figure A3: D. delphis, left IC tracts shown in blue, right IC tracts shown in red, set to a more liberal threshold of minimum 0.1% and maximum 5% of waytotals. Still 3-dimensional view. S4 Figure A4: D. delphis, left IC tracts shown in blue, right IC tracts shown in red, set to a more liberal threshold of minimum 0.1% and maximum 5% of waytotals. Rotating 3-dimensional view. S4 Figure B1: S. attenuata, left IC tracts shown in blue, right IC tracts shown in red, minimum threshold set to 1% and maximum threshold set to 30% of waytotals. Orthographic view. S4 Figure B2: S. attenuata, left IC tracts shown in blue, right IC tracts shown in red, set to a more liberal threshold of minimum 0.1% and maximum 5% of waytotals. Orthographic view. S4 Figure B3: S. attenuata, left IC tracts shown in blue, right IC tracts shown in red, set to a more liberal threshold of minimum 0.1% and maximum 5% of waytotals. Still [file pone.0323617.s001.zip › supporting_information/s5_fig_c4.gif]

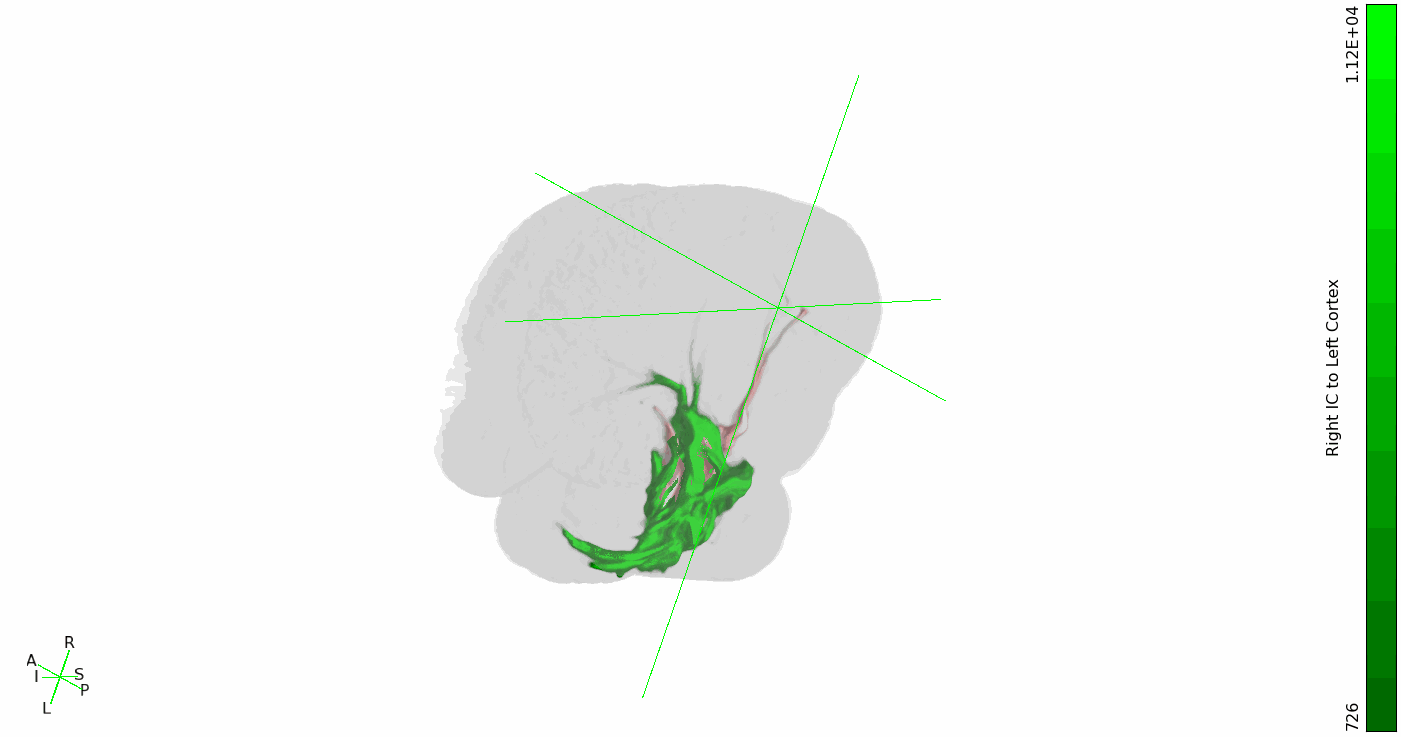

Supplement: S1 File — S1 Text. Detailed cerebellar and subcortical projection sites in IC-cerebellar traces. S2 Text. Detailed cortical projections in IC-cerebellar traces. S3 Figures. Masked regions of interest in FSLeyes. S3 Figure A. D. delphis. Red= right, blue=left for cerebella, yellow=right and turquoise=left for inferior colliculi. S3 Figure B. S. Attenuata. Red= right, blue=left for cerebella, yellow=right and turquoise=left for inferior colliculi. S3 Figure C. L. acutus. Red= right, blue=left for cerebella, yellow=right and turquoise=left for inferior colliculi. S3 Figure D. B. borealis. Red= right, blue=left for cerebella, yellow=right and turquoise=left for inferior colliculi. S4 Figures. Ascending auditory tractograms. S4 Figure A1: D. delphis, left IC tracts shown in blue, right IC tracts shown in red, minimum threshold set to 1% and maximum threshold set to 30% of waytotals. Orthographic view. S4 Figure A2: D. delphis, left IC tracts shown in blue, right IC tracts shown in red, set to a more liberal threshold of minimum 0.1% and maximum 5% of waytotals. Orthographic view. S4 Figure A3: D. delphis, left IC tracts shown in blue, right IC tracts shown in red, set to a more liberal threshold of minimum 0.1% and maximum 5% of waytotals. Still 3-dimensional view. S4 Figure A4: D. delphis, left IC tracts shown in blue, right IC tracts shown in red, set to a more liberal threshold of minimum 0.1% and maximum 5% of waytotals. Rotating 3-dimensional view. S4 Figure B1: S. attenuata, left IC tracts shown in blue, right IC tracts shown in red, minimum threshold set to 1% and maximum threshold set to 30% of waytotals. Orthographic view. S4 Figure B2: S. attenuata, left IC tracts shown in blue, right IC tracts shown in red, set to a more liberal threshold of minimum 0.1% and maximum 5% of waytotals. Orthographic view. S4 Figure B3: S. attenuata, left IC tracts shown in blue, right IC tracts shown in red, set to a more liberal threshold of minimum 0.1% and maximum 5% of waytotals. Still [file pone.0323617.s001.zip › supporting_information/s5_fig_d4.gif]

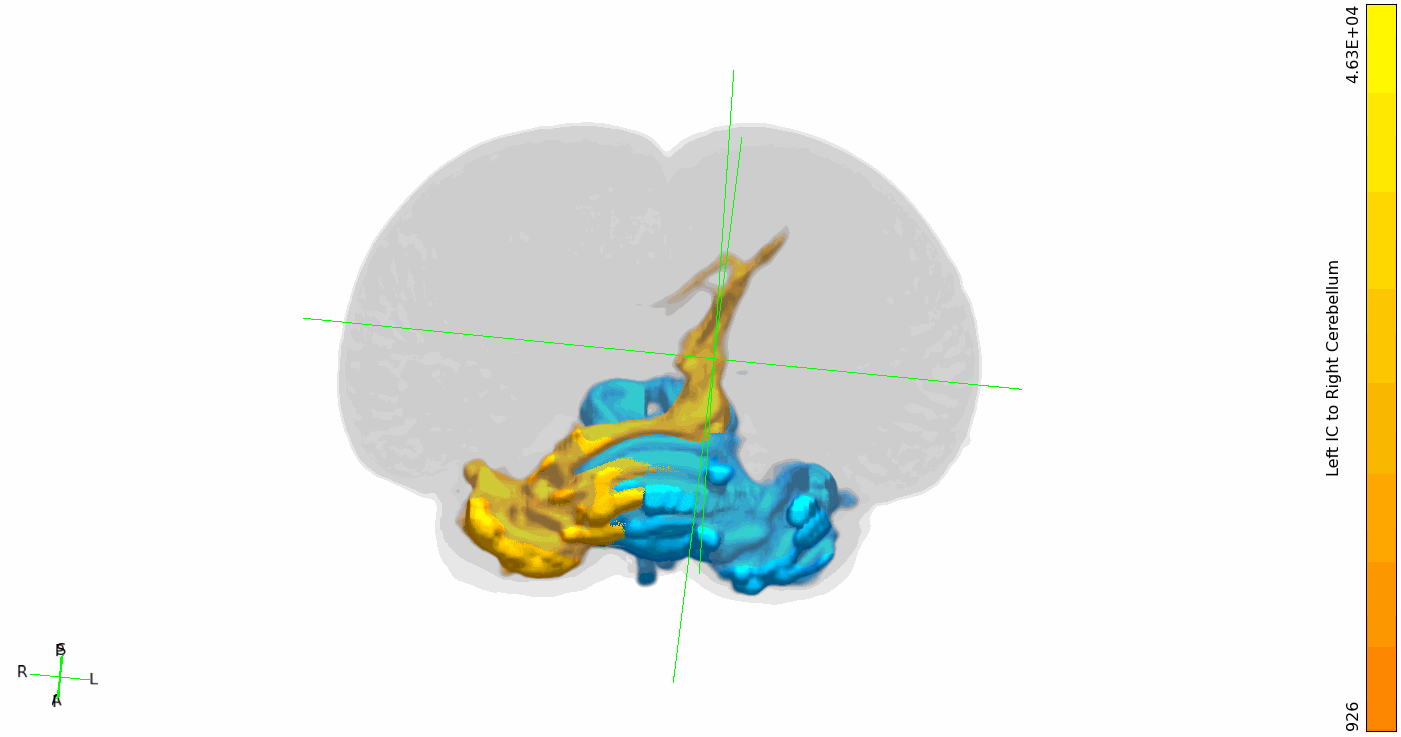

Supplement: S1 File — S1 Text. Detailed cerebellar and subcortical projection sites in IC-cerebellar traces. S2 Text. Detailed cortical projections in IC-cerebellar traces. S3 Figures. Masked regions of interest in FSLeyes. S3 Figure A. D. delphis. Red= right, blue=left for cerebella, yellow=right and turquoise=left for inferior colliculi. S3 Figure B. S. Attenuata. Red= right, blue=left for cerebella, yellow=right and turquoise=left for inferior colliculi. S3 Figure C. L. acutus. Red= right, blue=left for cerebella, yellow=right and turquoise=left for inferior colliculi. S3 Figure D. B. borealis. Red= right, blue=left for cerebella, yellow=right and turquoise=left for inferior colliculi. S4 Figures. Ascending auditory tractograms. S4 Figure A1: D. delphis, left IC tracts shown in blue, right IC tracts shown in red, minimum threshold set to 1% and maximum threshold set to 30% of waytotals. Orthographic view. S4 Figure A2: D. delphis, left IC tracts shown in blue, right IC tracts shown in red, set to a more liberal threshold of minimum 0.1% and maximum 5% of waytotals. Orthographic view. S4 Figure A3: D. delphis, left IC tracts shown in blue, right IC tracts shown in red, set to a more liberal threshold of minimum 0.1% and maximum 5% of waytotals. Still 3-dimensional view. S4 Figure A4: D. delphis, left IC tracts shown in blue, right IC tracts shown in red, set to a more liberal threshold of minimum 0.1% and maximum 5% of waytotals. Rotating 3-dimensional view. S4 Figure B1: S. attenuata, left IC tracts shown in blue, right IC tracts shown in red, minimum threshold set to 1% and maximum threshold set to 30% of waytotals. Orthographic view. S4 Figure B2: S. attenuata, left IC tracts shown in blue, right IC tracts shown in red, set to a more liberal threshold of minimum 0.1% and maximum 5% of waytotals. Orthographic view. S4 Figure B3: S. attenuata, left IC tracts shown in blue, right IC tracts shown in red, set to a more liberal threshold of minimum 0.1% and maximum 5% of waytotals. Still [file pone.0323617.s001.zip › supporting_information/s6_fig_a4.gif]

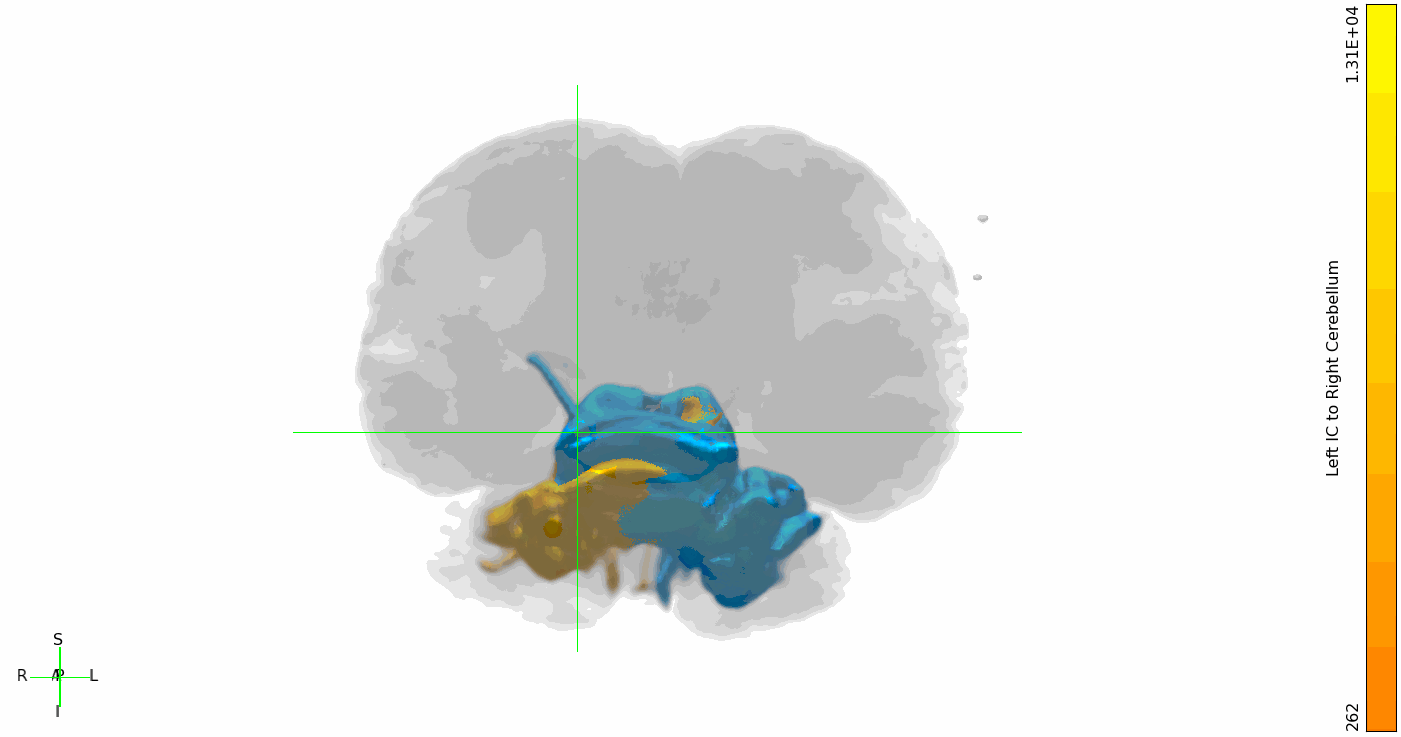

Supplement: S1 File — S1 Text. Detailed cerebellar and subcortical projection sites in IC-cerebellar traces. S2 Text. Detailed cortical projections in IC-cerebellar traces. S3 Figures. Masked regions of interest in FSLeyes. S3 Figure A. D. delphis. Red= right, blue=left for cerebella, yellow=right and turquoise=left for inferior colliculi. S3 Figure B. S. Attenuata. Red= right, blue=left for cerebella, yellow=right and turquoise=left for inferior colliculi. S3 Figure C. L. acutus. Red= right, blue=left for cerebella, yellow=right and turquoise=left for inferior colliculi. S3 Figure D. B. borealis. Red= right, blue=left for cerebella, yellow=right and turquoise=left for inferior colliculi. S4 Figures. Ascending auditory tractograms. S4 Figure A1: D. delphis, left IC tracts shown in blue, right IC tracts shown in red, minimum threshold set to 1% and maximum threshold set to 30% of waytotals. Orthographic view. S4 Figure A2: D. delphis, left IC tracts shown in blue, right IC tracts shown in red, set to a more liberal threshold of minimum 0.1% and maximum 5% of waytotals. Orthographic view. S4 Figure A3: D. delphis, left IC tracts shown in blue, right IC tracts shown in red, set to a more liberal threshold of minimum 0.1% and maximum 5% of waytotals. Still 3-dimensional view. S4 Figure A4: D. delphis, left IC tracts shown in blue, right IC tracts shown in red, set to a more liberal threshold of minimum 0.1% and maximum 5% of waytotals. Rotating 3-dimensional view. S4 Figure B1: S. attenuata, left IC tracts shown in blue, right IC tracts shown in red, minimum threshold set to 1% and maximum threshold set to 30% of waytotals. Orthographic view. S4 Figure B2: S. attenuata, left IC tracts shown in blue, right IC tracts shown in red, set to a more liberal threshold of minimum 0.1% and maximum 5% of waytotals. Orthographic view. S4 Figure B3: S. attenuata, left IC tracts shown in blue, right IC tracts shown in red, set to a more liberal threshold of minimum 0.1% and maximum 5% of waytotals. Still [file pone.0323617.s001.zip › supporting_information/s6_fig_b4.gif]

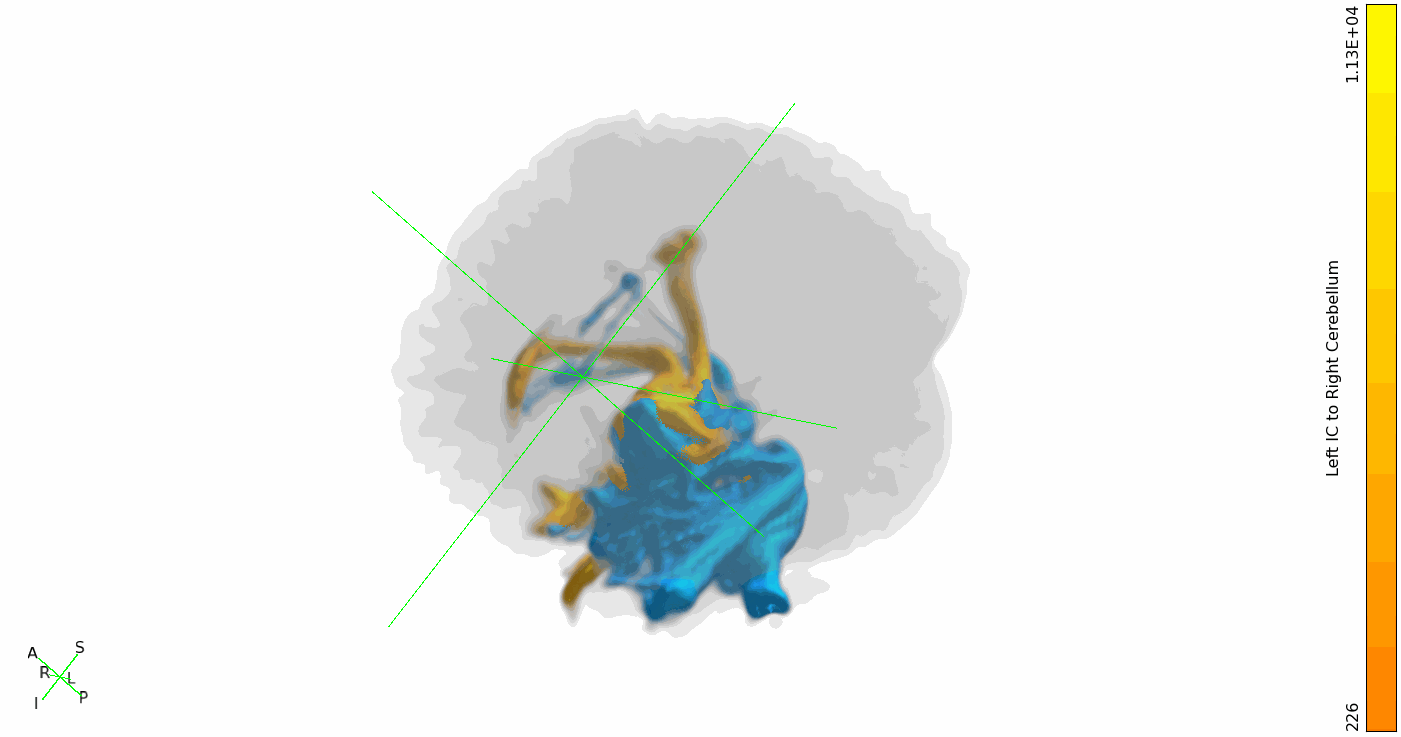

Supplement: S1 File — S1 Text. Detailed cerebellar and subcortical projection sites in IC-cerebellar traces. S2 Text. Detailed cortical projections in IC-cerebellar traces. S3 Figures. Masked regions of interest in FSLeyes. S3 Figure A. D. delphis. Red= right, blue=left for cerebella, yellow=right and turquoise=left for inferior colliculi. S3 Figure B. S. Attenuata. Red= right, blue=left for cerebella, yellow=right and turquoise=left for inferior colliculi. S3 Figure C. L. acutus. Red= right, blue=left for cerebella, yellow=right and turquoise=left for inferior colliculi. S3 Figure D. B. borealis. Red= right, blue=left for cerebella, yellow=right and turquoise=left for inferior colliculi. S4 Figures. Ascending auditory tractograms. S4 Figure A1: D. delphis, left IC tracts shown in blue, right IC tracts shown in red, minimum threshold set to 1% and maximum threshold set to 30% of waytotals. Orthographic view. S4 Figure A2: D. delphis, left IC tracts shown in blue, right IC tracts shown in red, set to a more liberal threshold of minimum 0.1% and maximum 5% of waytotals. Orthographic view. S4 Figure A3: D. delphis, left IC tracts shown in blue, right IC tracts shown in red, set to a more liberal threshold of minimum 0.1% and maximum 5% of waytotals. Still 3-dimensional view. S4 Figure A4: D. delphis, left IC tracts shown in blue, right IC tracts shown in red, set to a more liberal threshold of minimum 0.1% and maximum 5% of waytotals. Rotating 3-dimensional view. S4 Figure B1: S. attenuata, left IC tracts shown in blue, right IC tracts shown in red, minimum threshold set to 1% and maximum threshold set to 30% of waytotals. Orthographic view. S4 Figure B2: S. attenuata, left IC tracts shown in blue, right IC tracts shown in red, set to a more liberal threshold of minimum 0.1% and maximum 5% of waytotals. Orthographic view. S4 Figure B3: S. attenuata, left IC tracts shown in blue, right IC tracts shown in red, set to a more liberal threshold of minimum 0.1% and maximum 5% of waytotals. Still [file pone.0323617.s001.zip › supporting_information/s6_fig_c4.gif]

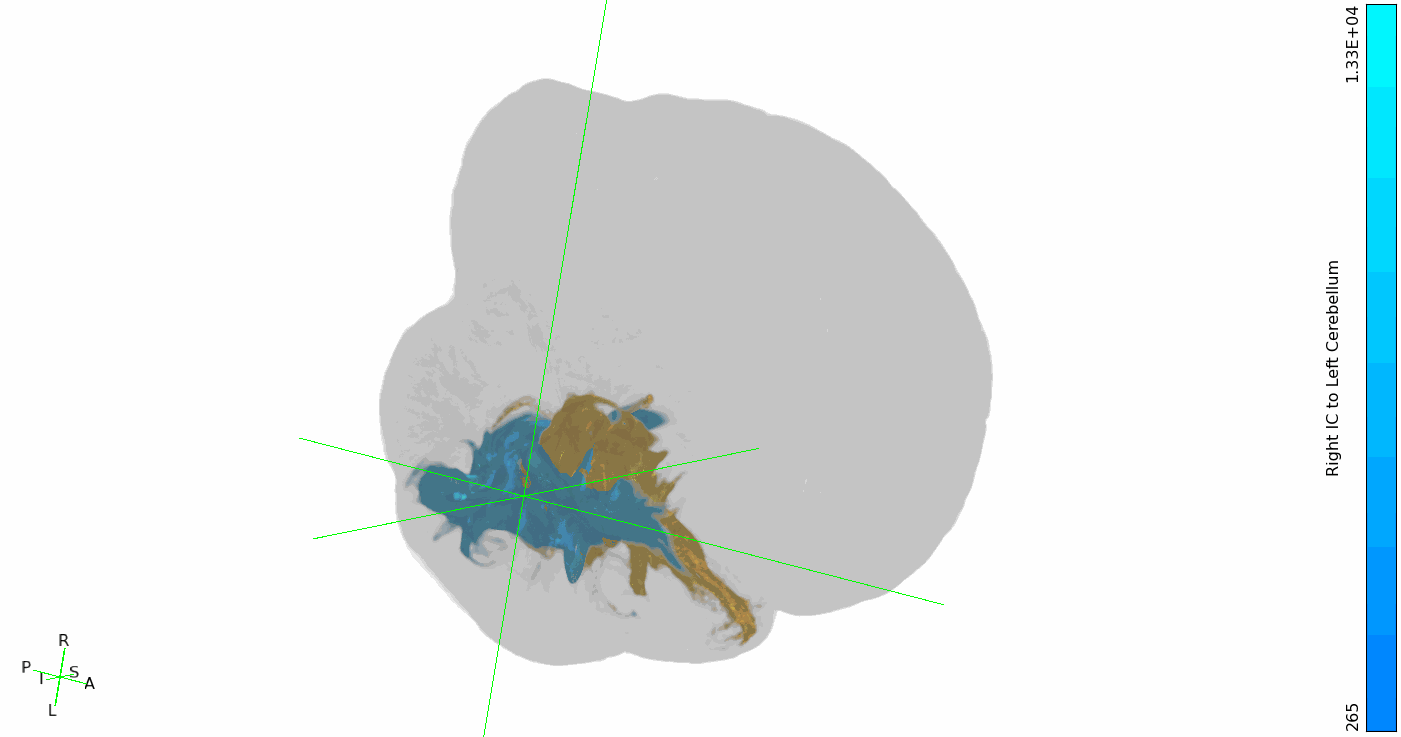

Supplement: S1 File — S1 Text. Detailed cerebellar and subcortical projection sites in IC-cerebellar traces. S2 Text. Detailed cortical projections in IC-cerebellar traces. S3 Figures. Masked regions of interest in FSLeyes. S3 Figure A. D. delphis. Red= right, blue=left for cerebella, yellow=right and turquoise=left for inferior colliculi. S3 Figure B. S. Attenuata. Red= right, blue=left for cerebella, yellow=right and turquoise=left for inferior colliculi. S3 Figure C. L. acutus. Red= right, blue=left for cerebella, yellow=right and turquoise=left for inferior colliculi. S3 Figure D. B. borealis. Red= right, blue=left for cerebella, yellow=right and turquoise=left for inferior colliculi. S4 Figures. Ascending auditory tractograms. S4 Figure A1: D. delphis, left IC tracts shown in blue, right IC tracts shown in red, minimum threshold set to 1% and maximum threshold set to 30% of waytotals. Orthographic view. S4 Figure A2: D. delphis, left IC tracts shown in blue, right IC tracts shown in red, set to a more liberal threshold of minimum 0.1% and maximum 5% of waytotals. Orthographic view. S4 Figure A3: D. delphis, left IC tracts shown in blue, right IC tracts shown in red, set to a more liberal threshold of minimum 0.1% and maximum 5% of waytotals. Still 3-dimensional view. S4 Figure A4: D. delphis, left IC tracts shown in blue, right IC tracts shown in red, set to a more liberal threshold of minimum 0.1% and maximum 5% of waytotals. Rotating 3-dimensional view. S4 Figure B1: S. attenuata, left IC tracts shown in blue, right IC tracts shown in red, minimum threshold set to 1% and maximum threshold set to 30% of waytotals. Orthographic view. S4 Figure B2: S. attenuata, left IC tracts shown in blue, right IC tracts shown in red, set to a more liberal threshold of minimum 0.1% and maximum 5% of waytotals. Orthographic view. S4 Figure B3: S. attenuata, left IC tracts shown in blue, right IC tracts shown in red, set to a more liberal threshold of minimum 0.1% and maximum 5% of waytotals. Still [file pone.0323617.s001.zip › supporting_information/s6_fig_d4.gif]

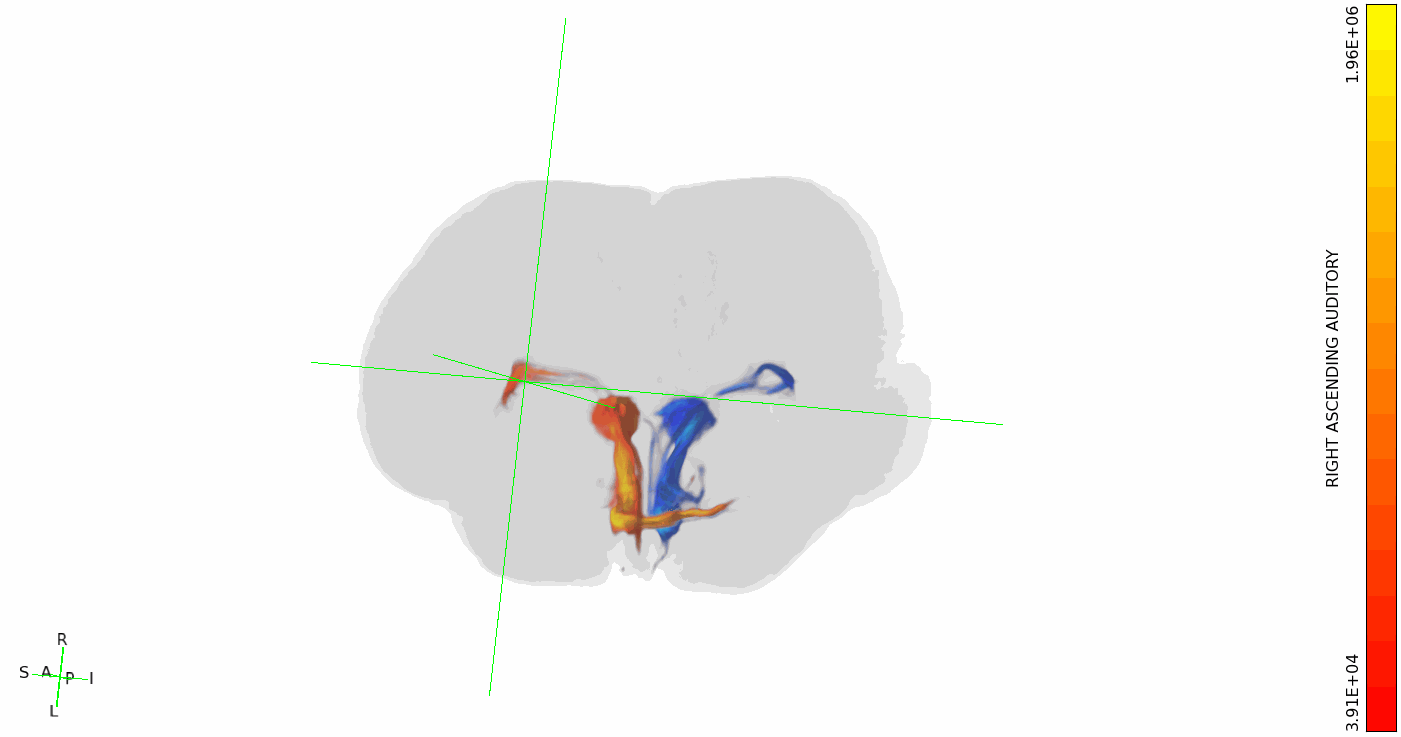

Supplement: S1 File — S1 Text. Detailed cerebellar and subcortical projection sites in IC-cerebellar traces. S2 Text. Detailed cortical projections in IC-cerebellar traces. S3 Figures. Masked regions of interest in FSLeyes. S3 Figure A. D. delphis. Red= right, blue=left for cerebella, yellow=right and turquoise=left for inferior colliculi. S3 Figure B. S. Attenuata. Red= right, blue=left for cerebella, yellow=right and turquoise=left for inferior colliculi. S3 Figure C. L. acutus. Red= right, blue=left for cerebella, yellow=right and turquoise=left for inferior colliculi. S3 Figure D. B. borealis. Red= right, blue=left for cerebella, yellow=right and turquoise=left for inferior colliculi. S4 Figures. Ascending auditory tractograms. S4 Figure A1: D. delphis, left IC tracts shown in blue, right IC tracts shown in red, minimum threshold set to 1% and maximum threshold set to 30% of waytotals. Orthographic view. S4 Figure A2: D. delphis, left IC tracts shown in blue, right IC tracts shown in red, set to a more liberal threshold of minimum 0.1% and maximum 5% of waytotals. Orthographic view. S4 Figure A3: D. delphis, left IC tracts shown in blue, right IC tracts shown in red, set to a more liberal threshold of minimum 0.1% and maximum 5% of waytotals. Still 3-dimensional view. S4 Figure A4: D. delphis, left IC tracts shown in blue, right IC tracts shown in red, set to a more liberal threshold of minimum 0.1% and maximum 5% of waytotals. Rotating 3-dimensional view. S4 Figure B1: S. attenuata, left IC tracts shown in blue, right IC tracts shown in red, minimum threshold set to 1% and maximum threshold set to 30% of waytotals. Orthographic view. S4 Figure B2: S. attenuata, left IC tracts shown in blue, right IC tracts shown in red, set to a more liberal threshold of minimum 0.1% and maximum 5% of waytotals. Orthographic view. S4 Figure B3: S. attenuata, left IC tracts shown in blue, right IC tracts shown in red, set to a more liberal threshold of minimum 0.1% and maximum 5% of waytotals. Still [file pone.0323617.s001.zip › supporting_information/s4_fig_d4.gif]

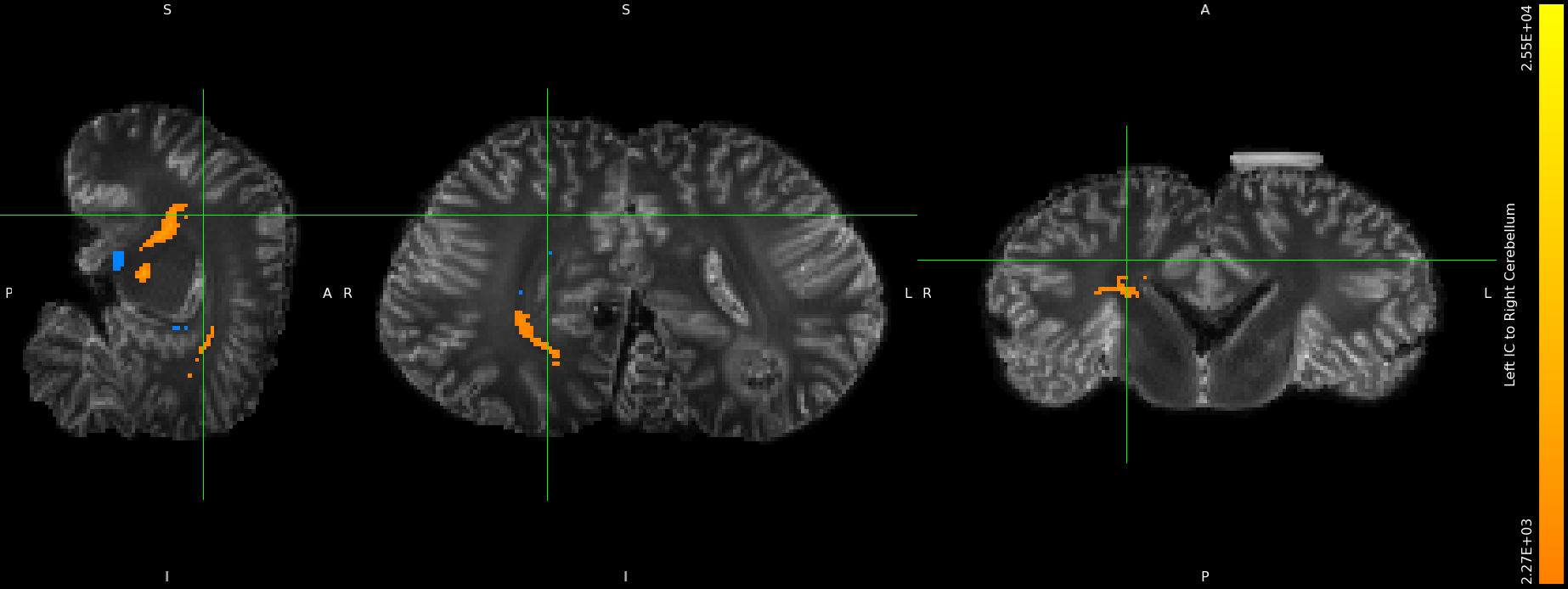

Supplement: S1 File — S1 Text. Detailed cerebellar and subcortical projection sites in IC-cerebellar traces. S2 Text. Detailed cortical projections in IC-cerebellar traces. S3 Figures. Masked regions of interest in FSLeyes. S3 Figure A. D. delphis. Red= right, blue=left for cerebella, yellow=right and turquoise=left for inferior colliculi. S3 Figure B. S. Attenuata. Red= right, blue=left for cerebella, yellow=right and turquoise=left for inferior colliculi. S3 Figure C. L. acutus. Red= right, blue=left for cerebella, yellow=right and turquoise=left for inferior colliculi. S3 Figure D. B. borealis. Red= right, blue=left for cerebella, yellow=right and turquoise=left for inferior colliculi. S4 Figures. Ascending auditory tractograms. S4 Figure A1: D. delphis, left IC tracts shown in blue, right IC tracts shown in red, minimum threshold set to 1% and maximum threshold set to 30% of waytotals. Orthographic view. S4 Figure A2: D. delphis, left IC tracts shown in blue, right IC tracts shown in red, set to a more liberal threshold of minimum 0.1% and maximum 5% of waytotals. Orthographic view. S4 Figure A3: D. delphis, left IC tracts shown in blue, right IC tracts shown in red, set to a more liberal threshold of minimum 0.1% and maximum 5% of waytotals. Still 3-dimensional view. S4 Figure A4: D. delphis, left IC tracts shown in blue, right IC tracts shown in red, set to a more liberal threshold of minimum 0.1% and maximum 5% of waytotals. Rotating 3-dimensional view. S4 Figure B1: S. attenuata, left IC tracts shown in blue, right IC tracts shown in red, minimum threshold set to 1% and maximum threshold set to 30% of waytotals. Orthographic view. S4 Figure B2: S. attenuata, left IC tracts shown in blue, right IC tracts shown in red, set to a more liberal threshold of minimum 0.1% and maximum 5% of waytotals. Orthographic view. S4 Figure B3: S. attenuata, left IC tracts shown in blue, right IC tracts shown in red, set to a more liberal threshold of minimum 0.1% and maximum 5% of waytotals. Still [file pone.0323617.s001.zip › supporting_information/s6_fig_c1.png]

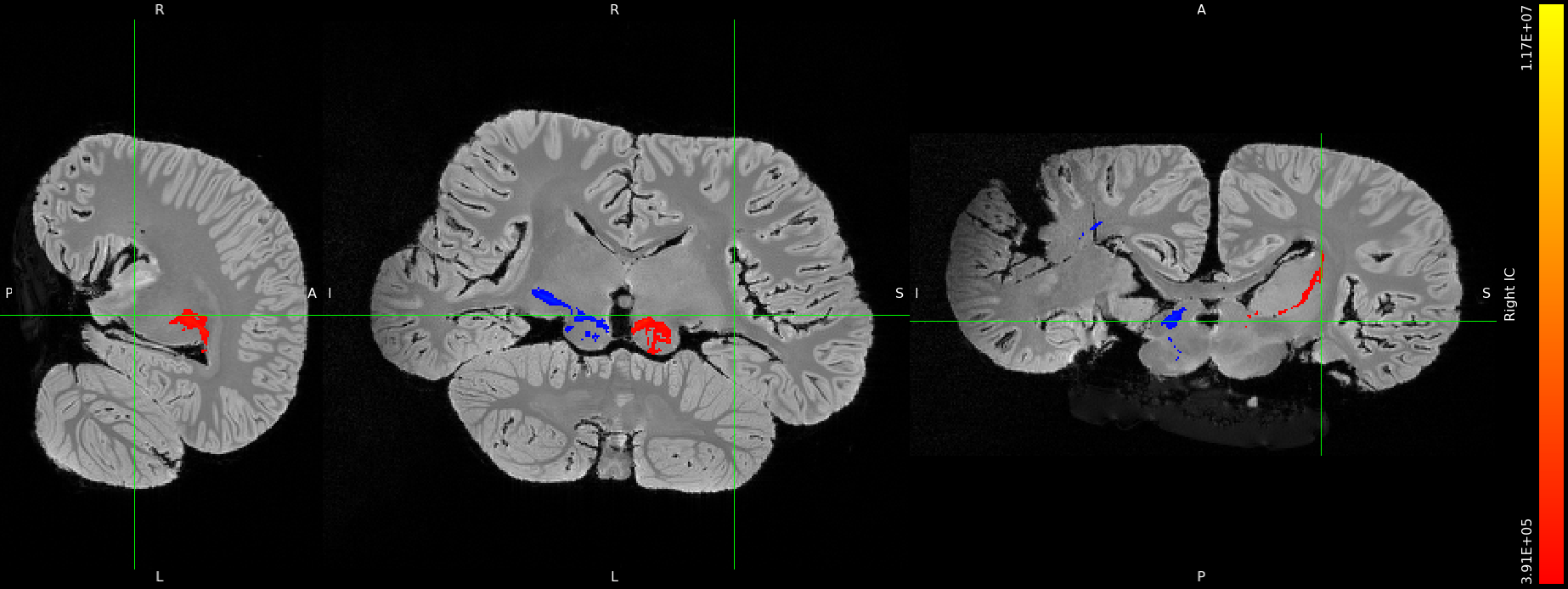

Supplement: S1 File — S1 Text. Detailed cerebellar and subcortical projection sites in IC-cerebellar traces. S2 Text. Detailed cortical projections in IC-cerebellar traces. S3 Figures. Masked regions of interest in FSLeyes. S3 Figure A. D. delphis. Red= right, blue=left for cerebella, yellow=right and turquoise=left for inferior colliculi. S3 Figure B. S. Attenuata. Red= right, blue=left for cerebella, yellow=right and turquoise=left for inferior colliculi. S3 Figure C. L. acutus. Red= right, blue=left for cerebella, yellow=right and turquoise=left for inferior colliculi. S3 Figure D. B. borealis. Red= right, blue=left for cerebella, yellow=right and turquoise=left for inferior colliculi. S4 Figures. Ascending auditory tractograms. S4 Figure A1: D. delphis, left IC tracts shown in blue, right IC tracts shown in red, minimum threshold set to 1% and maximum threshold set to 30% of waytotals. Orthographic view. S4 Figure A2: D. delphis, left IC tracts shown in blue, right IC tracts shown in red, set to a more liberal threshold of minimum 0.1% and maximum 5% of waytotals. Orthographic view. S4 Figure A3: D. delphis, left IC tracts shown in blue, right IC tracts shown in red, set to a more liberal threshold of minimum 0.1% and maximum 5% of waytotals. Still 3-dimensional view. S4 Figure A4: D. delphis, left IC tracts shown in blue, right IC tracts shown in red, set to a more liberal threshold of minimum 0.1% and maximum 5% of waytotals. Rotating 3-dimensional view. S4 Figure B1: S. attenuata, left IC tracts shown in blue, right IC tracts shown in red, minimum threshold set to 1% and maximum threshold set to 30% of waytotals. Orthographic view. S4 Figure B2: S. attenuata, left IC tracts shown in blue, right IC tracts shown in red, set to a more liberal threshold of minimum 0.1% and maximum 5% of waytotals. Orthographic view. S4 Figure B3: S. attenuata, left IC tracts shown in blue, right IC tracts shown in red, set to a more liberal threshold of minimum 0.1% and maximum 5% of waytotals. Still [file pone.0323617.s001.zip › supporting_information/s4_fig_d2.png]

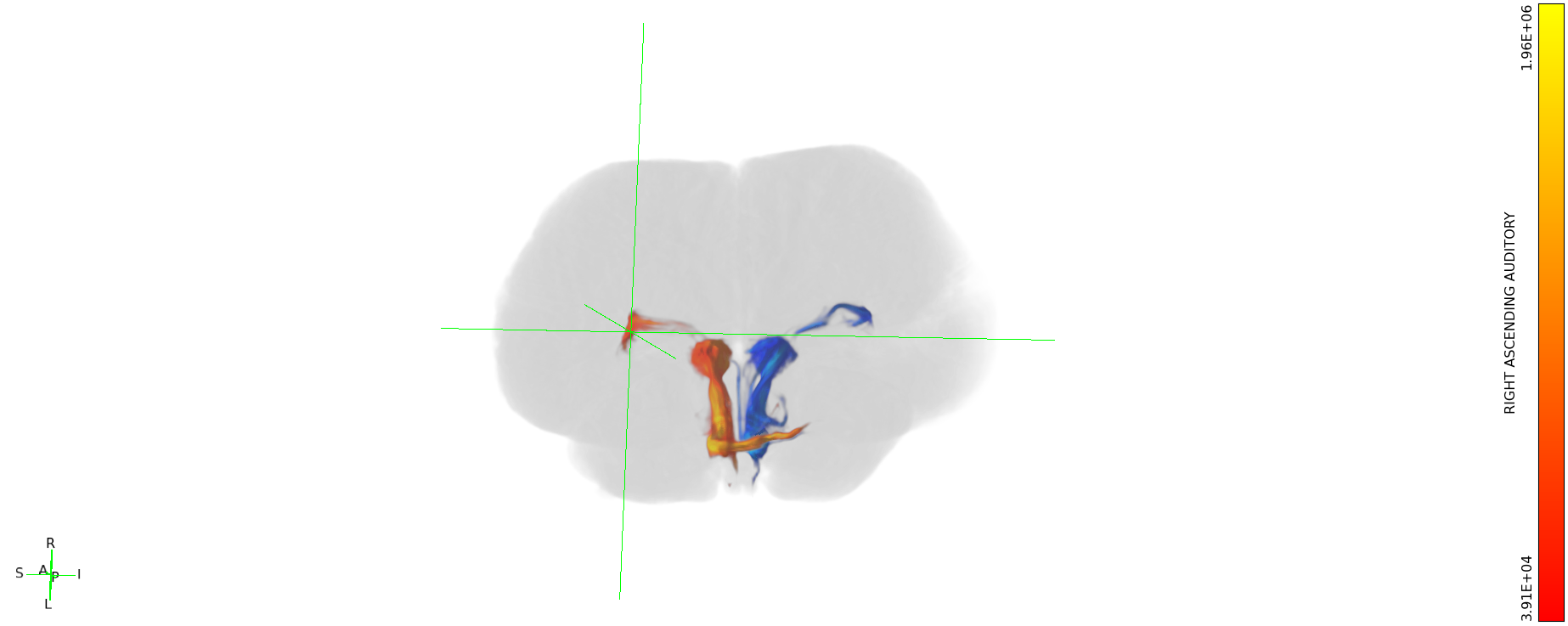

Supplement: S1 File — S1 Text. Detailed cerebellar and subcortical projection sites in IC-cerebellar traces. S2 Text. Detailed cortical projections in IC-cerebellar traces. S3 Figures. Masked regions of interest in FSLeyes. S3 Figure A. D. delphis. Red= right, blue=left for cerebella, yellow=right and turquoise=left for inferior colliculi. S3 Figure B. S. Attenuata. Red= right, blue=left for cerebella, yellow=right and turquoise=left for inferior colliculi. S3 Figure C. L. acutus. Red= right, blue=left for cerebella, yellow=right and turquoise=left for inferior colliculi. S3 Figure D. B. borealis. Red= right, blue=left for cerebella, yellow=right and turquoise=left for inferior colliculi. S4 Figures. Ascending auditory tractograms. S4 Figure A1: D. delphis, left IC tracts shown in blue, right IC tracts shown in red, minimum threshold set to 1% and maximum threshold set to 30% of waytotals. Orthographic view. S4 Figure A2: D. delphis, left IC tracts shown in blue, right IC tracts shown in red, set to a more liberal threshold of minimum 0.1% and maximum 5% of waytotals. Orthographic view. S4 Figure A3: D. delphis, left IC tracts shown in blue, right IC tracts shown in red, set to a more liberal threshold of minimum 0.1% and maximum 5% of waytotals. Still 3-dimensional view. S4 Figure A4: D. delphis, left IC tracts shown in blue, right IC tracts shown in red, set to a more liberal threshold of minimum 0.1% and maximum 5% of waytotals. Rotating 3-dimensional view. S4 Figure B1: S. attenuata, left IC tracts shown in blue, right IC tracts shown in red, minimum threshold set to 1% and maximum threshold set to 30% of waytotals. Orthographic view. S4 Figure B2: S. attenuata, left IC tracts shown in blue, right IC tracts shown in red, set to a more liberal threshold of minimum 0.1% and maximum 5% of waytotals. Orthographic view. S4 Figure B3: S. attenuata, left IC tracts shown in blue, right IC tracts shown in red, set to a more liberal threshold of minimum 0.1% and maximum 5% of waytotals. Still [file pone.0323617.s001.zip › supporting_information/s4_fig_d3.png]

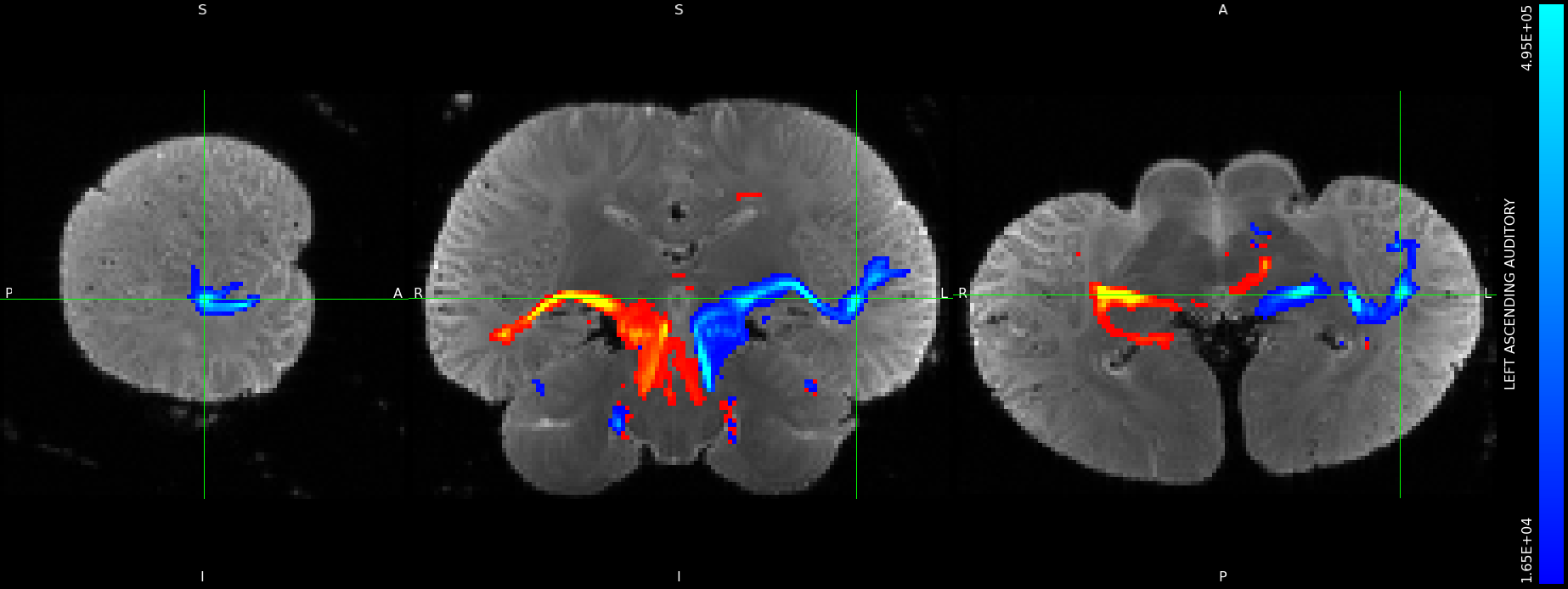

Supplement: S1 File — S1 Text. Detailed cerebellar and subcortical projection sites in IC-cerebellar traces. S2 Text. Detailed cortical projections in IC-cerebellar traces. S3 Figures. Masked regions of interest in FSLeyes. S3 Figure A. D. delphis. Red= right, blue=left for cerebella, yellow=right and turquoise=left for inferior colliculi. S3 Figure B. S. Attenuata. Red= right, blue=left for cerebella, yellow=right and turquoise=left for inferior colliculi. S3 Figure C. L. acutus. Red= right, blue=left for cerebella, yellow=right and turquoise=left for inferior colliculi. S3 Figure D. B. borealis. Red= right, blue=left for cerebella, yellow=right and turquoise=left for inferior colliculi. S4 Figures. Ascending auditory tractograms. S4 Figure A1: D. delphis, left IC tracts shown in blue, right IC tracts shown in red, minimum threshold set to 1% and maximum threshold set to 30% of waytotals. Orthographic view. S4 Figure A2: D. delphis, left IC tracts shown in blue, right IC tracts shown in red, set to a more liberal threshold of minimum 0.1% and maximum 5% of waytotals. Orthographic view. S4 Figure A3: D. delphis, left IC tracts shown in blue, right IC tracts shown in red, set to a more liberal threshold of minimum 0.1% and maximum 5% of waytotals. Still 3-dimensional view. S4 Figure A4: D. delphis, left IC tracts shown in blue, right IC tracts shown in red, set to a more liberal threshold of minimum 0.1% and maximum 5% of waytotals. Rotating 3-dimensional view. S4 Figure B1: S. attenuata, left IC tracts shown in blue, right IC tracts shown in red, minimum threshold set to 1% and maximum threshold set to 30% of waytotals. Orthographic view. S4 Figure B2: S. attenuata, left IC tracts shown in blue, right IC tracts shown in red, set to a more liberal threshold of minimum 0.1% and maximum 5% of waytotals. Orthographic view. S4 Figure B3: S. attenuata, left IC tracts shown in blue, right IC tracts shown in red, set to a more liberal threshold of minimum 0.1% and maximum 5% of waytotals. Still [file pone.0323617.s001.zip › supporting_information/s4_fig_a1.png]

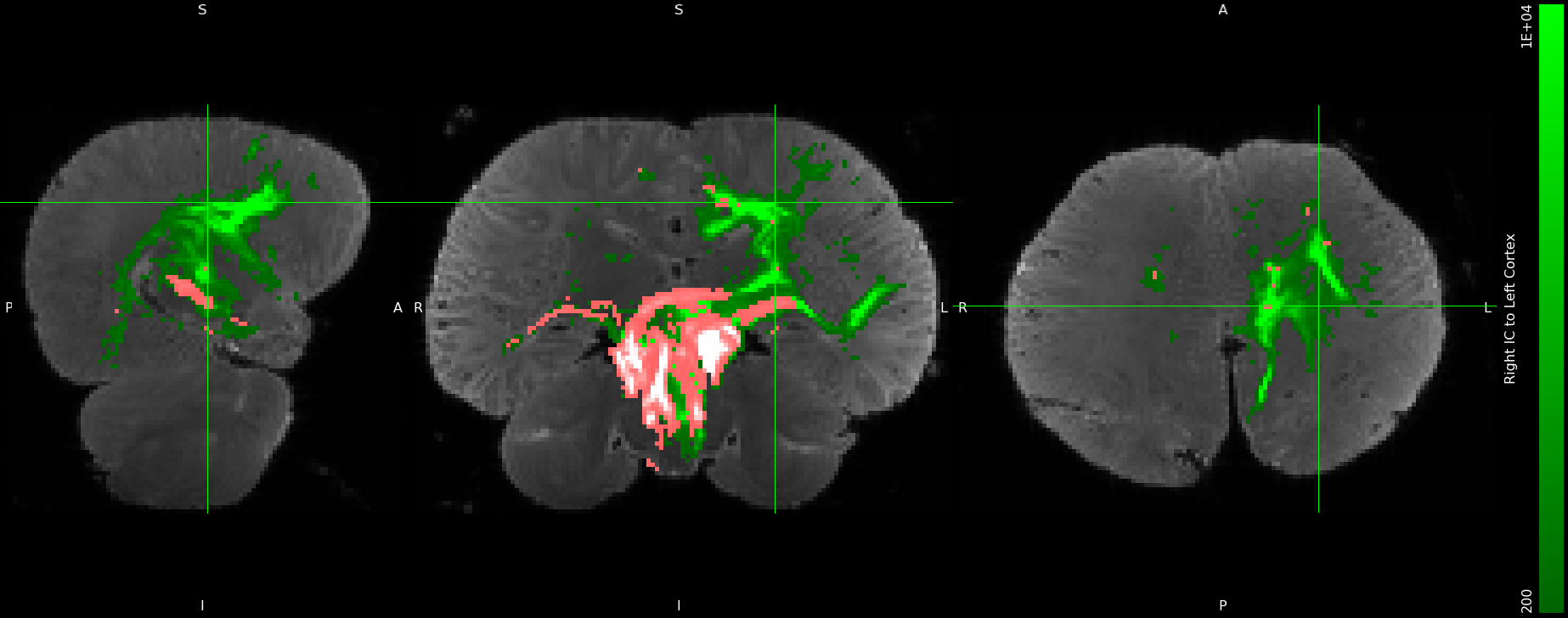

Supplement: S1 File — S1 Text. Detailed cerebellar and subcortical projection sites in IC-cerebellar traces. S2 Text. Detailed cortical projections in IC-cerebellar traces. S3 Figures. Masked regions of interest in FSLeyes. S3 Figure A. D. delphis. Red= right, blue=left for cerebella, yellow=right and turquoise=left for inferior colliculi. S3 Figure B. S. Attenuata. Red= right, blue=left for cerebella, yellow=right and turquoise=left for inferior colliculi. S3 Figure C. L. acutus. Red= right, blue=left for cerebella, yellow=right and turquoise=left for inferior colliculi. S3 Figure D. B. borealis. Red= right, blue=left for cerebella, yellow=right and turquoise=left for inferior colliculi. S4 Figures. Ascending auditory tractograms. S4 Figure A1: D. delphis, left IC tracts shown in blue, right IC tracts shown in red, minimum threshold set to 1% and maximum threshold set to 30% of waytotals. Orthographic view. S4 Figure A2: D. delphis, left IC tracts shown in blue, right IC tracts shown in red, set to a more liberal threshold of minimum 0.1% and maximum 5% of waytotals. Orthographic view. S4 Figure A3: D. delphis, left IC tracts shown in blue, right IC tracts shown in red, set to a more liberal threshold of minimum 0.1% and maximum 5% of waytotals. Still 3-dimensional view. S4 Figure A4: D. delphis, left IC tracts shown in blue, right IC tracts shown in red, set to a more liberal threshold of minimum 0.1% and maximum 5% of waytotals. Rotating 3-dimensional view. S4 Figure B1: S. attenuata, left IC tracts shown in blue, right IC tracts shown in red, minimum threshold set to 1% and maximum threshold set to 30% of waytotals. Orthographic view. S4 Figure B2: S. attenuata, left IC tracts shown in blue, right IC tracts shown in red, set to a more liberal threshold of minimum 0.1% and maximum 5% of waytotals. Orthographic view. S4 Figure B3: S. attenuata, left IC tracts shown in blue, right IC tracts shown in red, set to a more liberal threshold of minimum 0.1% and maximum 5% of waytotals. Still [file pone.0323617.s001.zip › supporting_information/s5_fig_a2.png]

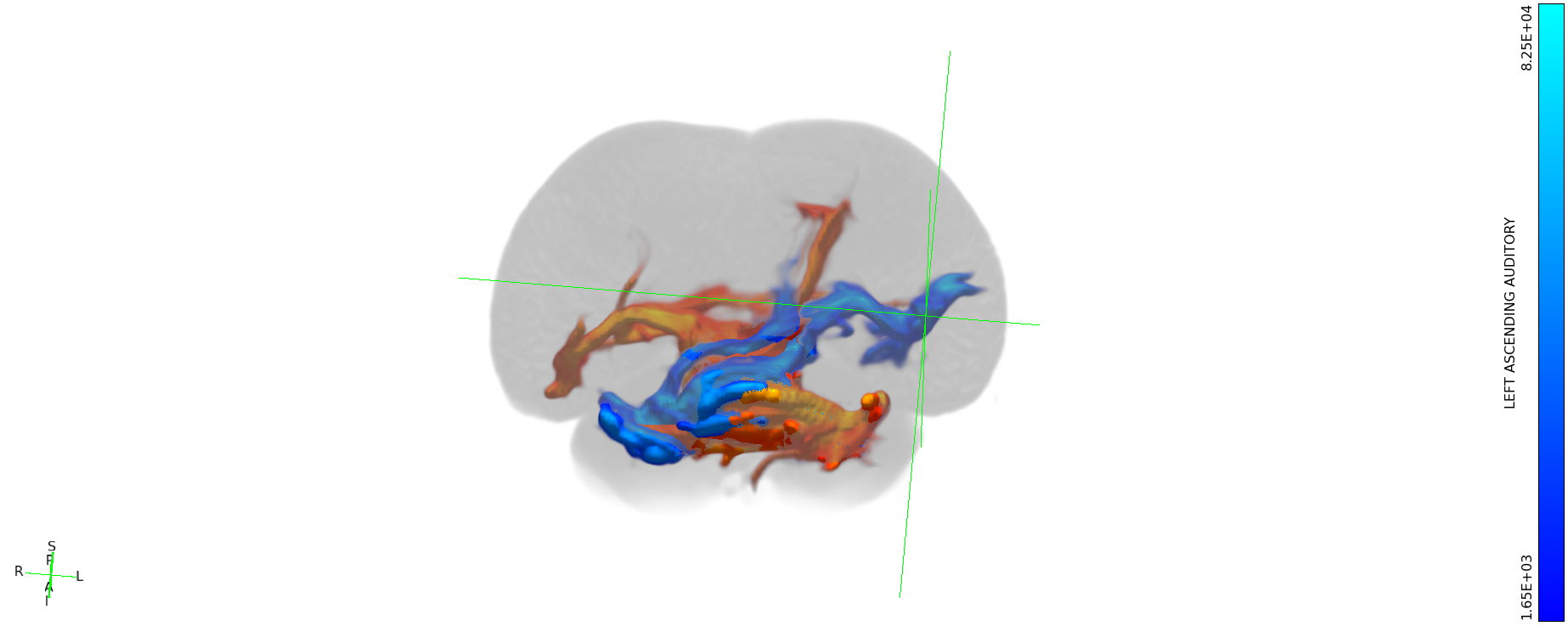

Supplement: S1 File — S1 Text. Detailed cerebellar and subcortical projection sites in IC-cerebellar traces. S2 Text. Detailed cortical projections in IC-cerebellar traces. S3 Figures. Masked regions of interest in FSLeyes. S3 Figure A. D. delphis. Red= right, blue=left for cerebella, yellow=right and turquoise=left for inferior colliculi. S3 Figure B. S. Attenuata. Red= right, blue=left for cerebella, yellow=right and turquoise=left for inferior colliculi. S3 Figure C. L. acutus. Red= right, blue=left for cerebella, yellow=right and turquoise=left for inferior colliculi. S3 Figure D. B. borealis. Red= right, blue=left for cerebella, yellow=right and turquoise=left for inferior colliculi. S4 Figures. Ascending auditory tractograms. S4 Figure A1: D. delphis, left IC tracts shown in blue, right IC tracts shown in red, minimum threshold set to 1% and maximum threshold set to 30% of waytotals. Orthographic view. S4 Figure A2: D. delphis, left IC tracts shown in blue, right IC tracts shown in red, set to a more liberal threshold of minimum 0.1% and maximum 5% of waytotals. Orthographic view. S4 Figure A3: D. delphis, left IC tracts shown in blue, right IC tracts shown in red, set to a more liberal threshold of minimum 0.1% and maximum 5% of waytotals. Still 3-dimensional view. S4 Figure A4: D. delphis, left IC tracts shown in blue, right IC tracts shown in red, set to a more liberal threshold of minimum 0.1% and maximum 5% of waytotals. Rotating 3-dimensional view. S4 Figure B1: S. attenuata, left IC tracts shown in blue, right IC tracts shown in red, minimum threshold set to 1% and maximum threshold set to 30% of waytotals. Orthographic view. S4 Figure B2: S. attenuata, left IC tracts shown in blue, right IC tracts shown in red, set to a more liberal threshold of minimum 0.1% and maximum 5% of waytotals. Orthographic view. S4 Figure B3: S. attenuata, left IC tracts shown in blue, right IC tracts shown in red, set to a more liberal threshold of minimum 0.1% and maximum 5% of waytotals. Still [file pone.0323617.s001.zip › supporting_information/s4_fig_a3.png]

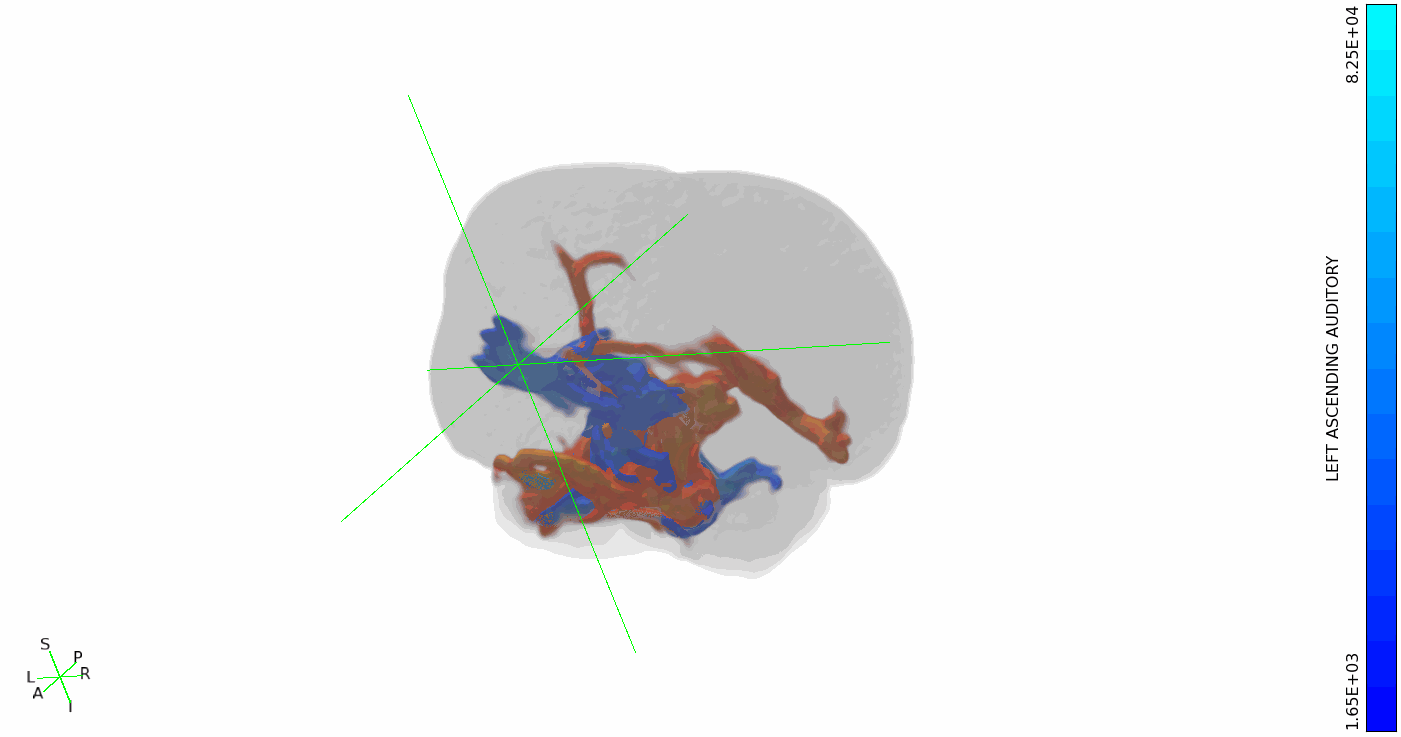

Supplement: S1 File — S1 Text. Detailed cerebellar and subcortical projection sites in IC-cerebellar traces. S2 Text. Detailed cortical projections in IC-cerebellar traces. S3 Figures. Masked regions of interest in FSLeyes. S3 Figure A. D. delphis. Red= right, blue=left for cerebella, yellow=right and turquoise=left for inferior colliculi. S3 Figure B. S. Attenuata. Red= right, blue=left for cerebella, yellow=right and turquoise=left for inferior colliculi. S3 Figure C. L. acutus. Red= right, blue=left for cerebella, yellow=right and turquoise=left for inferior colliculi. S3 Figure D. B. borealis. Red= right, blue=left for cerebella, yellow=right and turquoise=left for inferior colliculi. S4 Figures. Ascending auditory tractograms. S4 Figure A1: D. delphis, left IC tracts shown in blue, right IC tracts shown in red, minimum threshold set to 1% and maximum threshold set to 30% of waytotals. Orthographic view. S4 Figure A2: D. delphis, left IC tracts shown in blue, right IC tracts shown in red, set to a more liberal threshold of minimum 0.1% and maximum 5% of waytotals. Orthographic view. S4 Figure A3: D. delphis, left IC tracts shown in blue, right IC tracts shown in red, set to a more liberal threshold of minimum 0.1% and maximum 5% of waytotals. Still 3-dimensional view. S4 Figure A4: D. delphis, left IC tracts shown in blue, right IC tracts shown in red, set to a more liberal threshold of minimum 0.1% and maximum 5% of waytotals. Rotating 3-dimensional view. S4 Figure B1: S. attenuata, left IC tracts shown in blue, right IC tracts shown in red, minimum threshold set to 1% and maximum threshold set to 30% of waytotals. Orthographic view. S4 Figure B2: S. attenuata, left IC tracts shown in blue, right IC tracts shown in red, set to a more liberal threshold of minimum 0.1% and maximum 5% of waytotals. Orthographic view. S4 Figure B3: S. attenuata, left IC tracts shown in blue, right IC tracts shown in red, set to a more liberal threshold of minimum 0.1% and maximum 5% of waytotals. Still [file pone.0323617.s001.zip › supporting_information/s4_fig_a4.gif]

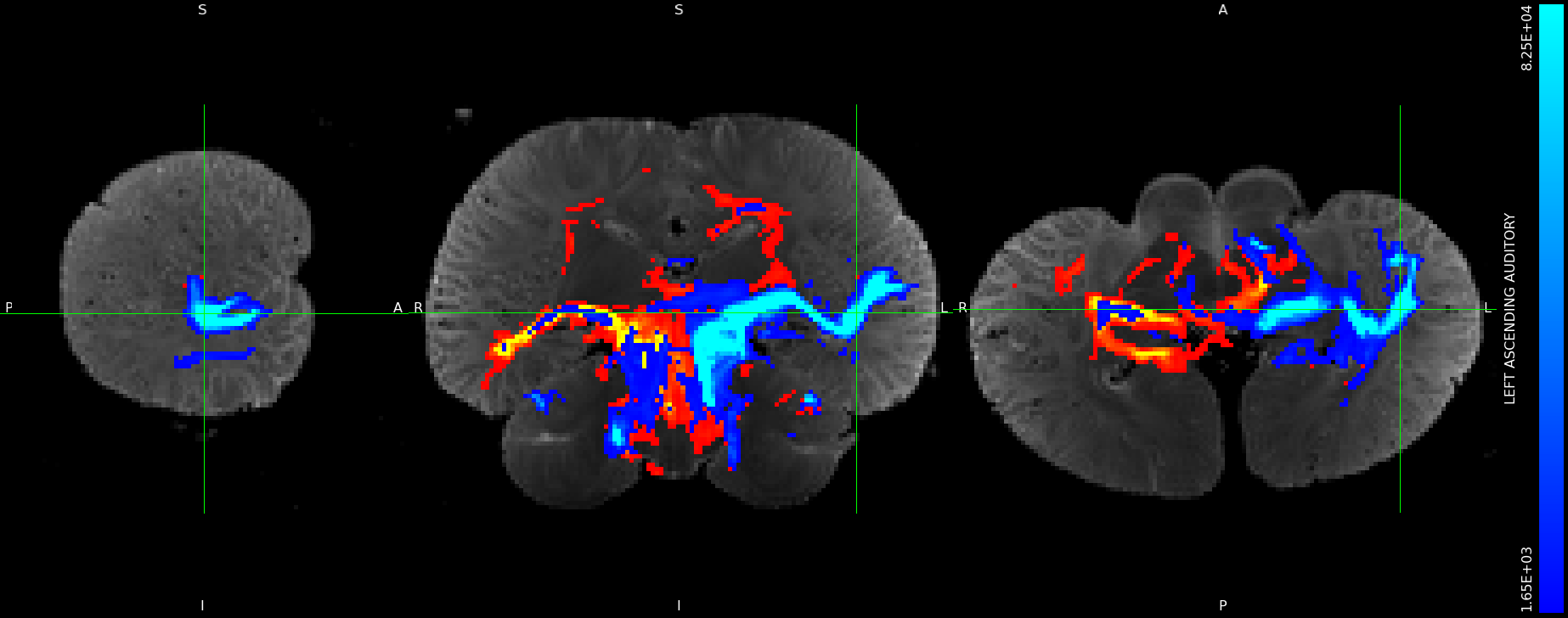

Supplement: S1 File — S1 Text. Detailed cerebellar and subcortical projection sites in IC-cerebellar traces. S2 Text. Detailed cortical projections in IC-cerebellar traces. S3 Figures. Masked regions of interest in FSLeyes. S3 Figure A. D. delphis. Red= right, blue=left for cerebella, yellow=right and turquoise=left for inferior colliculi. S3 Figure B. S. Attenuata. Red= right, blue=left for cerebella, yellow=right and turquoise=left for inferior colliculi. S3 Figure C. L. acutus. Red= right, blue=left for cerebella, yellow=right and turquoise=left for inferior colliculi. S3 Figure D. B. borealis. Red= right, blue=left for cerebella, yellow=right and turquoise=left for inferior colliculi. S4 Figures. Ascending auditory tractograms. S4 Figure A1: D. delphis, left IC tracts shown in blue, right IC tracts shown in red, minimum threshold set to 1% and maximum threshold set to 30% of waytotals. Orthographic view. S4 Figure A2: D. delphis, left IC tracts shown in blue, right IC tracts shown in red, set to a more liberal threshold of minimum 0.1% and maximum 5% of waytotals. Orthographic view. S4 Figure A3: D. delphis, left IC tracts shown in blue, right IC tracts shown in red, set to a more liberal threshold of minimum 0.1% and maximum 5% of waytotals. Still 3-dimensional view. S4 Figure A4: D. delphis, left IC tracts shown in blue, right IC tracts shown in red, set to a more liberal threshold of minimum 0.1% and maximum 5% of waytotals. Rotating 3-dimensional view. S4 Figure B1: S. attenuata, left IC tracts shown in blue, right IC tracts shown in red, minimum threshold set to 1% and maximum threshold set to 30% of waytotals. Orthographic view. S4 Figure B2: S. attenuata, left IC tracts shown in blue, right IC tracts shown in red, set to a more liberal threshold of minimum 0.1% and maximum 5% of waytotals. Orthographic view. S4 Figure B3: S. attenuata, left IC tracts shown in blue, right IC tracts shown in red, set to a more liberal threshold of minimum 0.1% and maximum 5% of waytotals. Still [file pone.0323617.s001.zip › supporting_information/s4_fig_a2.png]

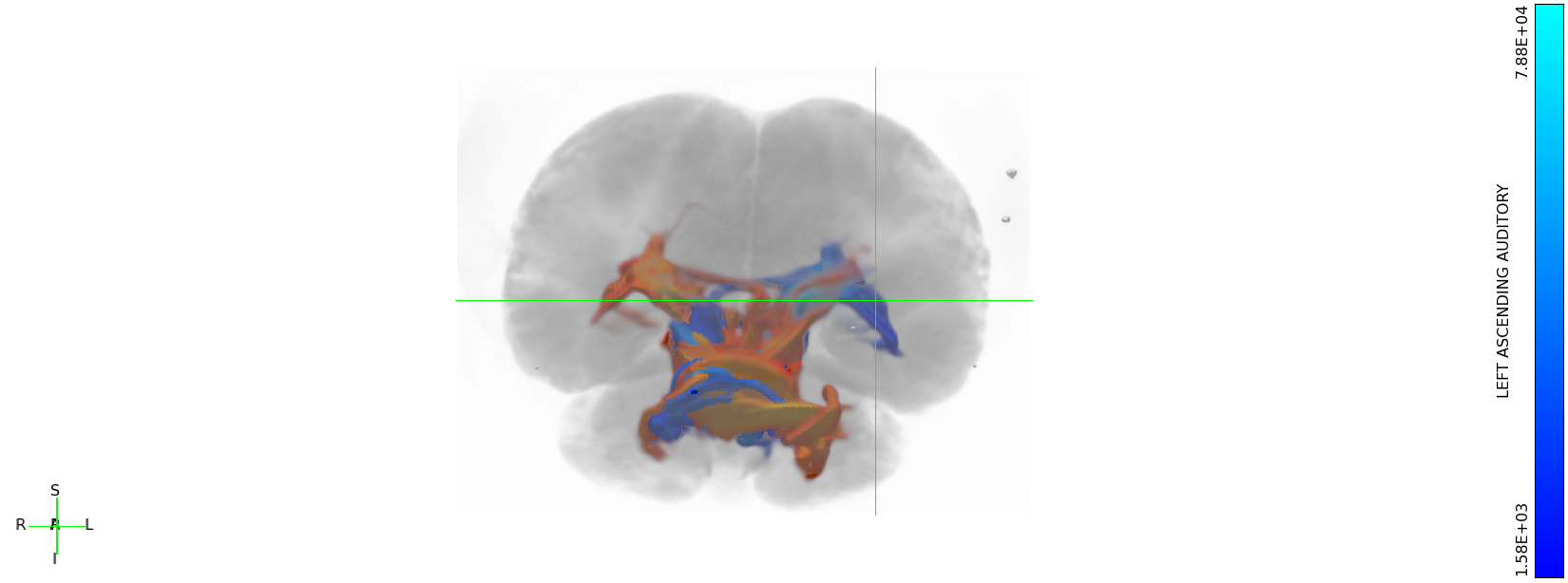

Supplement: S1 File — S1 Text. Detailed cerebellar and subcortical projection sites in IC-cerebellar traces. S2 Text. Detailed cortical projections in IC-cerebellar traces. S3 Figures. Masked regions of interest in FSLeyes. S3 Figure A. D. delphis. Red= right, blue=left for cerebella, yellow=right and turquoise=left for inferior colliculi. S3 Figure B. S. Attenuata. Red= right, blue=left for cerebella, yellow=right and turquoise=left for inferior colliculi. S3 Figure C. L. acutus. Red= right, blue=left for cerebella, yellow=right and turquoise=left for inferior colliculi. S3 Figure D. B. borealis. Red= right, blue=left for cerebella, yellow=right and turquoise=left for inferior colliculi. S4 Figures. Ascending auditory tractograms. S4 Figure A1: D. delphis, left IC tracts shown in blue, right IC tracts shown in red, minimum threshold set to 1% and maximum threshold set to 30% of waytotals. Orthographic view. S4 Figure A2: D. delphis, left IC tracts shown in blue, right IC tracts shown in red, set to a more liberal threshold of minimum 0.1% and maximum 5% of waytotals. Orthographic view. S4 Figure A3: D. delphis, left IC tracts shown in blue, right IC tracts shown in red, set to a more liberal threshold of minimum 0.1% and maximum 5% of waytotals. Still 3-dimensional view. S4 Figure A4: D. delphis, left IC tracts shown in blue, right IC tracts shown in red, set to a more liberal threshold of minimum 0.1% and maximum 5% of waytotals. Rotating 3-dimensional view. S4 Figure B1: S. attenuata, left IC tracts shown in blue, right IC tracts shown in red, minimum threshold set to 1% and maximum threshold set to 30% of waytotals. Orthographic view. S4 Figure B2: S. attenuata, left IC tracts shown in blue, right IC tracts shown in red, set to a more liberal threshold of minimum 0.1% and maximum 5% of waytotals. Orthographic view. S4 Figure B3: S. attenuata, left IC tracts shown in blue, right IC tracts shown in red, set to a more liberal threshold of minimum 0.1% and maximum 5% of waytotals. Still [file pone.0323617.s001.zip › supporting_information/s4_fig_b3.png]

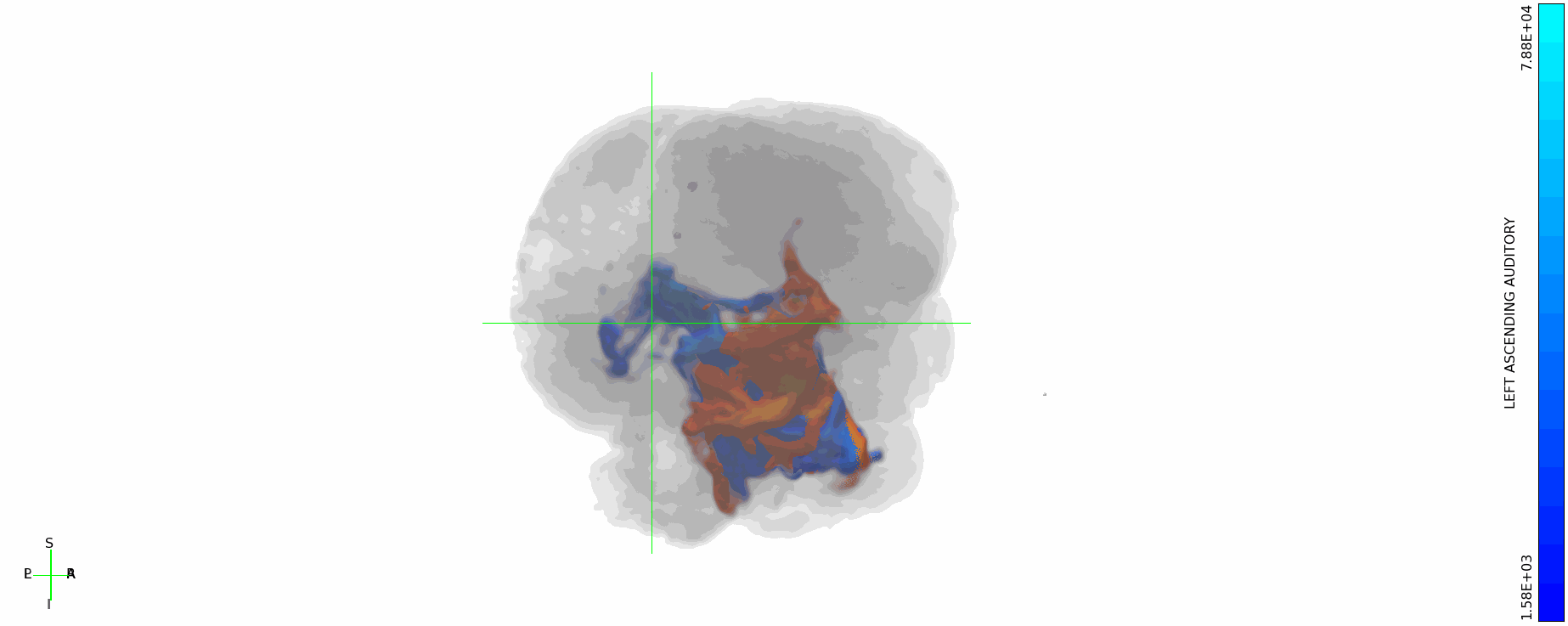

Supplement: S1 File — S1 Text. Detailed cerebellar and subcortical projection sites in IC-cerebellar traces. S2 Text. Detailed cortical projections in IC-cerebellar traces. S3 Figures. Masked regions of interest in FSLeyes. S3 Figure A. D. delphis. Red= right, blue=left for cerebella, yellow=right and turquoise=left for inferior colliculi. S3 Figure B. S. Attenuata. Red= right, blue=left for cerebella, yellow=right and turquoise=left for inferior colliculi. S3 Figure C. L. acutus. Red= right, blue=left for cerebella, yellow=right and turquoise=left for inferior colliculi. S3 Figure D. B. borealis. Red= right, blue=left for cerebella, yellow=right and turquoise=left for inferior colliculi. S4 Figures. Ascending auditory tractograms. S4 Figure A1: D. delphis, left IC tracts shown in blue, right IC tracts shown in red, minimum threshold set to 1% and maximum threshold set to 30% of waytotals. Orthographic view. S4 Figure A2: D. delphis, left IC tracts shown in blue, right IC tracts shown in red, set to a more liberal threshold of minimum 0.1% and maximum 5% of waytotals. Orthographic view. S4 Figure A3: D. delphis, left IC tracts shown in blue, right IC tracts shown in red, set to a more liberal threshold of minimum 0.1% and maximum 5% of waytotals. Still 3-dimensional view. S4 Figure A4: D. delphis, left IC tracts shown in blue, right IC tracts shown in red, set to a more liberal threshold of minimum 0.1% and maximum 5% of waytotals. Rotating 3-dimensional view. S4 Figure B1: S. attenuata, left IC tracts shown in blue, right IC tracts shown in red, minimum threshold set to 1% and maximum threshold set to 30% of waytotals. Orthographic view. S4 Figure B2: S. attenuata, left IC tracts shown in blue, right IC tracts shown in red, set to a more liberal threshold of minimum 0.1% and maximum 5% of waytotals. Orthographic view. S4 Figure B3: S. attenuata, left IC tracts shown in blue, right IC tracts shown in red, set to a more liberal threshold of minimum 0.1% and maximum 5% of waytotals. Still [file pone.0323617.s001.zip › supporting_information/s4_fig_b4.gif]

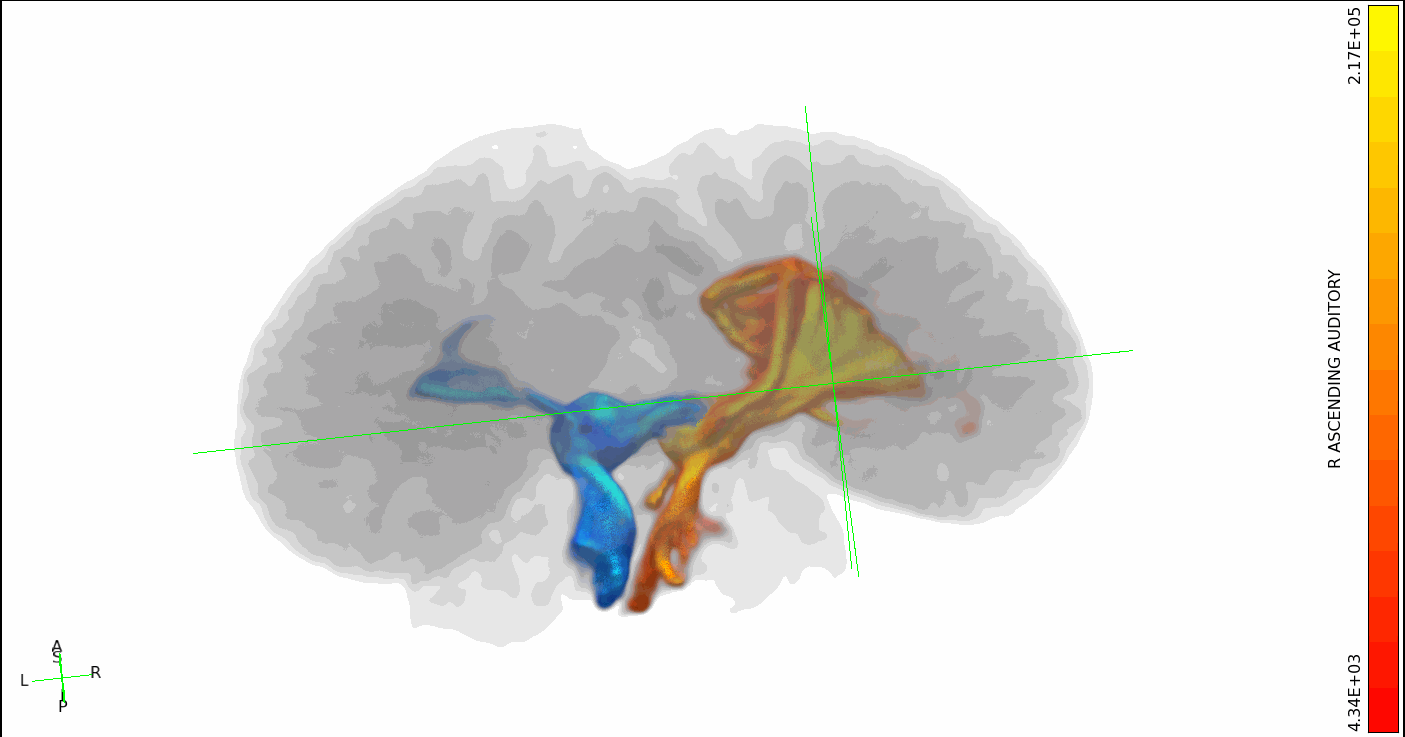

Supplement: S1 File — S1 Text. Detailed cerebellar and subcortical projection sites in IC-cerebellar traces. S2 Text. Detailed cortical projections in IC-cerebellar traces. S3 Figures. Masked regions of interest in FSLeyes. S3 Figure A. D. delphis. Red= right, blue=left for cerebella, yellow=right and turquoise=left for inferior colliculi. S3 Figure B. S. Attenuata. Red= right, blue=left for cerebella, yellow=right and turquoise=left for inferior colliculi. S3 Figure C. L. acutus. Red= right, blue=left for cerebella, yellow=right and turquoise=left for inferior colliculi. S3 Figure D. B. borealis. Red= right, blue=left for cerebella, yellow=right and turquoise=left for inferior colliculi. S4 Figures. Ascending auditory tractograms. S4 Figure A1: D. delphis, left IC tracts shown in blue, right IC tracts shown in red, minimum threshold set to 1% and maximum threshold set to 30% of waytotals. Orthographic view. S4 Figure A2: D. delphis, left IC tracts shown in blue, right IC tracts shown in red, set to a more liberal threshold of minimum 0.1% and maximum 5% of waytotals. Orthographic view. S4 Figure A3: D. delphis, left IC tracts shown in blue, right IC tracts shown in red, set to a more liberal threshold of minimum 0.1% and maximum 5% of waytotals. Still 3-dimensional view. S4 Figure A4: D. delphis, left IC tracts shown in blue, right IC tracts shown in red, set to a more liberal threshold of minimum 0.1% and maximum 5% of waytotals. Rotating 3-dimensional view. S4 Figure B1: S. attenuata, left IC tracts shown in blue, right IC tracts shown in red, minimum threshold set to 1% and maximum threshold set to 30% of waytotals. Orthographic view. S4 Figure B2: S. attenuata, left IC tracts shown in blue, right IC tracts shown in red, set to a more liberal threshold of minimum 0.1% and maximum 5% of waytotals. Orthographic view. S4 Figure B3: S. attenuata, left IC tracts shown in blue, right IC tracts shown in red, set to a more liberal threshold of minimum 0.1% and maximum 5% of waytotals. Still [file pone.0323617.s001.zip › supporting_information/s4_fig_c3.png]

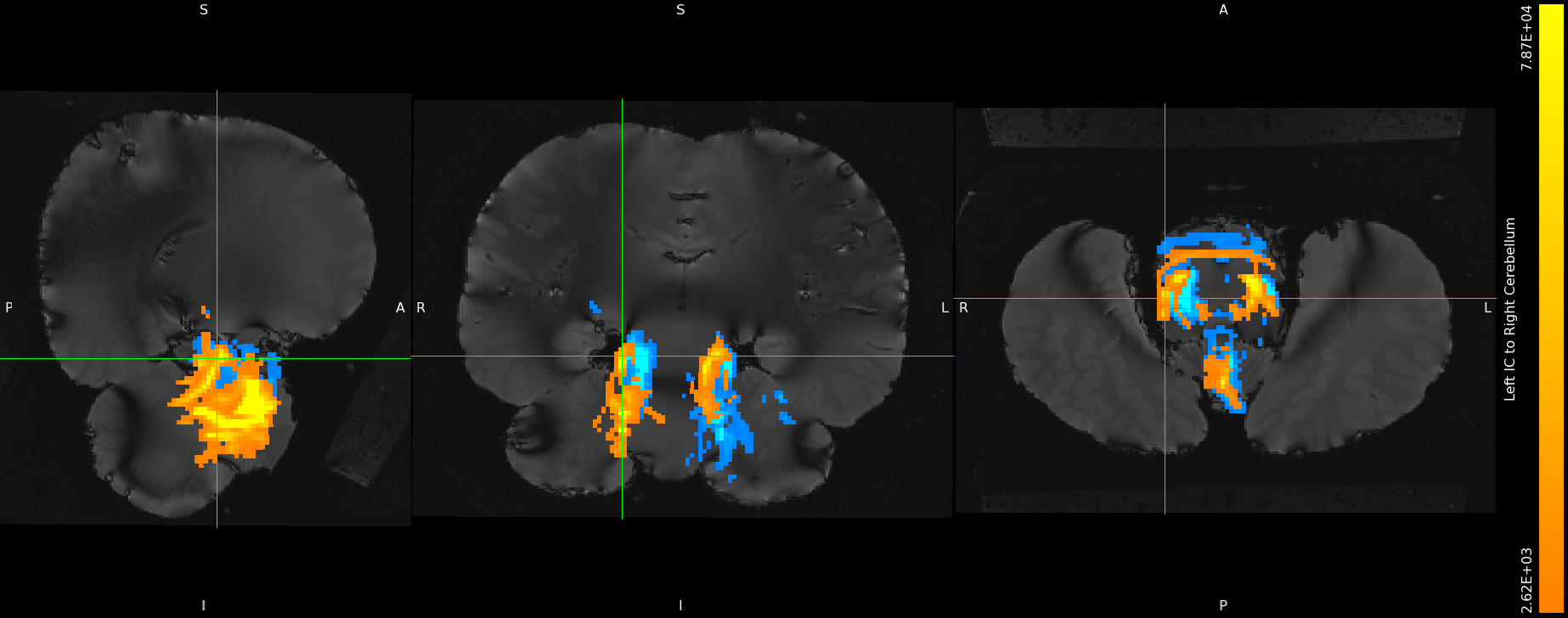

Supplement: S1 File — S1 Text. Detailed cerebellar and subcortical projection sites in IC-cerebellar traces. S2 Text. Detailed cortical projections in IC-cerebellar traces. S3 Figures. Masked regions of interest in FSLeyes. S3 Figure A. D. delphis. Red= right, blue=left for cerebella, yellow=right and turquoise=left for inferior colliculi. S3 Figure B. S. Attenuata. Red= right, blue=left for cerebella, yellow=right and turquoise=left for inferior colliculi. S3 Figure C. L. acutus. Red= right, blue=left for cerebella, yellow=right and turquoise=left for inferior colliculi. S3 Figure D. B. borealis. Red= right, blue=left for cerebella, yellow=right and turquoise=left for inferior colliculi. S4 Figures. Ascending auditory tractograms. S4 Figure A1: D. delphis, left IC tracts shown in blue, right IC tracts shown in red, minimum threshold set to 1% and maximum threshold set to 30% of waytotals. Orthographic view. S4 Figure A2: D. delphis, left IC tracts shown in blue, right IC tracts shown in red, set to a more liberal threshold of minimum 0.1% and maximum 5% of waytotals. Orthographic view. S4 Figure A3: D. delphis, left IC tracts shown in blue, right IC tracts shown in red, set to a more liberal threshold of minimum 0.1% and maximum 5% of waytotals. Still 3-dimensional view. S4 Figure A4: D. delphis, left IC tracts shown in blue, right IC tracts shown in red, set to a more liberal threshold of minimum 0.1% and maximum 5% of waytotals. Rotating 3-dimensional view. S4 Figure B1: S. attenuata, left IC tracts shown in blue, right IC tracts shown in red, minimum threshold set to 1% and maximum threshold set to 30% of waytotals. Orthographic view. S4 Figure B2: S. attenuata, left IC tracts shown in blue, right IC tracts shown in red, set to a more liberal threshold of minimum 0.1% and maximum 5% of waytotals. Orthographic view. S4 Figure B3: S. attenuata, left IC tracts shown in blue, right IC tracts shown in red, set to a more liberal threshold of minimum 0.1% and maximum 5% of waytotals. Still [file pone.0323617.s001.zip › supporting_information/s6_fig_b1.png]

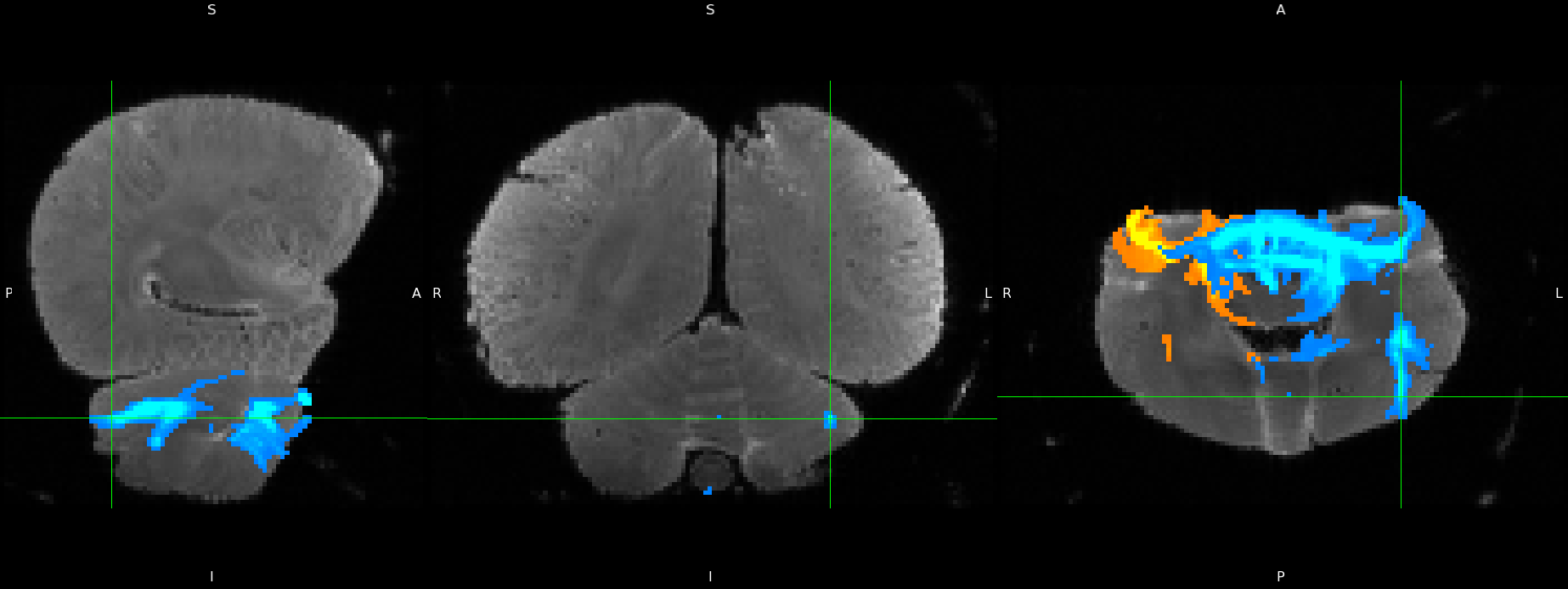

Supplement: S1 File — S1 Text. Detailed cerebellar and subcortical projection sites in IC-cerebellar traces. S2 Text. Detailed cortical projections in IC-cerebellar traces. S3 Figures. Masked regions of interest in FSLeyes. S3 Figure A. D. delphis. Red= right, blue=left for cerebella, yellow=right and turquoise=left for inferior colliculi. S3 Figure B. S. Attenuata. Red= right, blue=left for cerebella, yellow=right and turquoise=left for inferior colliculi. S3 Figure C. L. acutus. Red= right, blue=left for cerebella, yellow=right and turquoise=left for inferior colliculi. S3 Figure D. B. borealis. Red= right, blue=left for cerebella, yellow=right and turquoise=left for inferior colliculi. S4 Figures. Ascending auditory tractograms. S4 Figure A1: D. delphis, left IC tracts shown in blue, right IC tracts shown in red, minimum threshold set to 1% and maximum threshold set to 30% of waytotals. Orthographic view. S4 Figure A2: D. delphis, left IC tracts shown in blue, right IC tracts shown in red, set to a more liberal threshold of minimum 0.1% and maximum 5% of waytotals. Orthographic view. S4 Figure A3: D. delphis, left IC tracts shown in blue, right IC tracts shown in red, set to a more liberal threshold of minimum 0.1% and maximum 5% of waytotals. Still 3-dimensional view. S4 Figure A4: D. delphis, left IC tracts shown in blue, right IC tracts shown in red, set to a more liberal threshold of minimum 0.1% and maximum 5% of waytotals. Rotating 3-dimensional view. S4 Figure B1: S. attenuata, left IC tracts shown in blue, right IC tracts shown in red, minimum threshold set to 1% and maximum threshold set to 30% of waytotals. Orthographic view. S4 Figure B2: S. attenuata, left IC tracts shown in blue, right IC tracts shown in red, set to a more liberal threshold of minimum 0.1% and maximum 5% of waytotals. Orthographic view. S4 Figure B3: S. attenuata, left IC tracts shown in blue, right IC tracts shown in red, set to a more liberal threshold of minimum 0.1% and maximum 5% of waytotals. Still [file pone.0323617.s001.zip › supporting_information/s7_fig_a2.png]

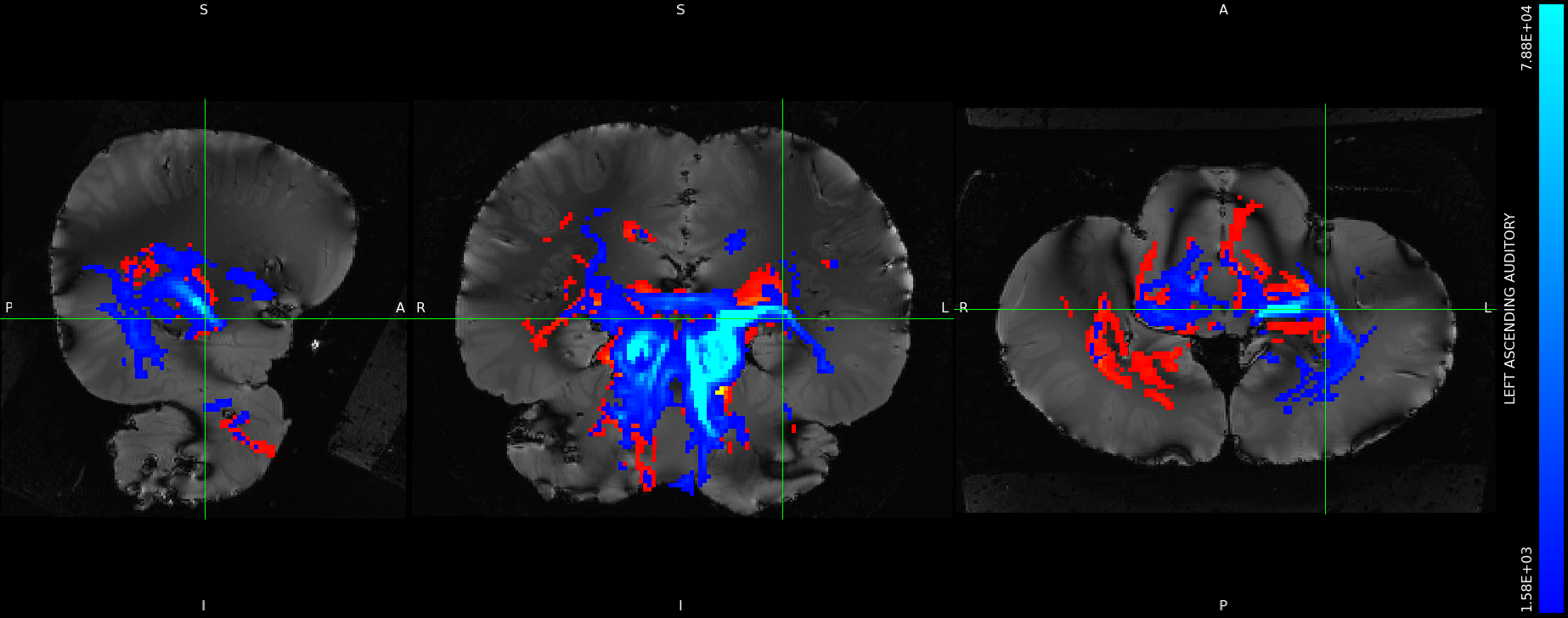

Supplement: S1 File — S1 Text. Detailed cerebellar and subcortical projection sites in IC-cerebellar traces. S2 Text. Detailed cortical projections in IC-cerebellar traces. S3 Figures. Masked regions of interest in FSLeyes. S3 Figure A. D. delphis. Red= right, blue=left for cerebella, yellow=right and turquoise=left for inferior colliculi. S3 Figure B. S. Attenuata. Red= right, blue=left for cerebella, yellow=right and turquoise=left for inferior colliculi. S3 Figure C. L. acutus. Red= right, blue=left for cerebella, yellow=right and turquoise=left for inferior colliculi. S3 Figure D. B. borealis. Red= right, blue=left for cerebella, yellow=right and turquoise=left for inferior colliculi. S4 Figures. Ascending auditory tractograms. S4 Figure A1: D. delphis, left IC tracts shown in blue, right IC tracts shown in red, minimum threshold set to 1% and maximum threshold set to 30% of waytotals. Orthographic view. S4 Figure A2: D. delphis, left IC tracts shown in blue, right IC tracts shown in red, set to a more liberal threshold of minimum 0.1% and maximum 5% of waytotals. Orthographic view. S4 Figure A3: D. delphis, left IC tracts shown in blue, right IC tracts shown in red, set to a more liberal threshold of minimum 0.1% and maximum 5% of waytotals. Still 3-dimensional view. S4 Figure A4: D. delphis, left IC tracts shown in blue, right IC tracts shown in red, set to a more liberal threshold of minimum 0.1% and maximum 5% of waytotals. Rotating 3-dimensional view. S4 Figure B1: S. attenuata, left IC tracts shown in blue, right IC tracts shown in red, minimum threshold set to 1% and maximum threshold set to 30% of waytotals. Orthographic view. S4 Figure B2: S. attenuata, left IC tracts shown in blue, right IC tracts shown in red, set to a more liberal threshold of minimum 0.1% and maximum 5% of waytotals. Orthographic view. S4 Figure B3: S. attenuata, left IC tracts shown in blue, right IC tracts shown in red, set to a more liberal threshold of minimum 0.1% and maximum 5% of waytotals. Still [file pone.0323617.s001.zip › supporting_information/s4_fig_b2.png]

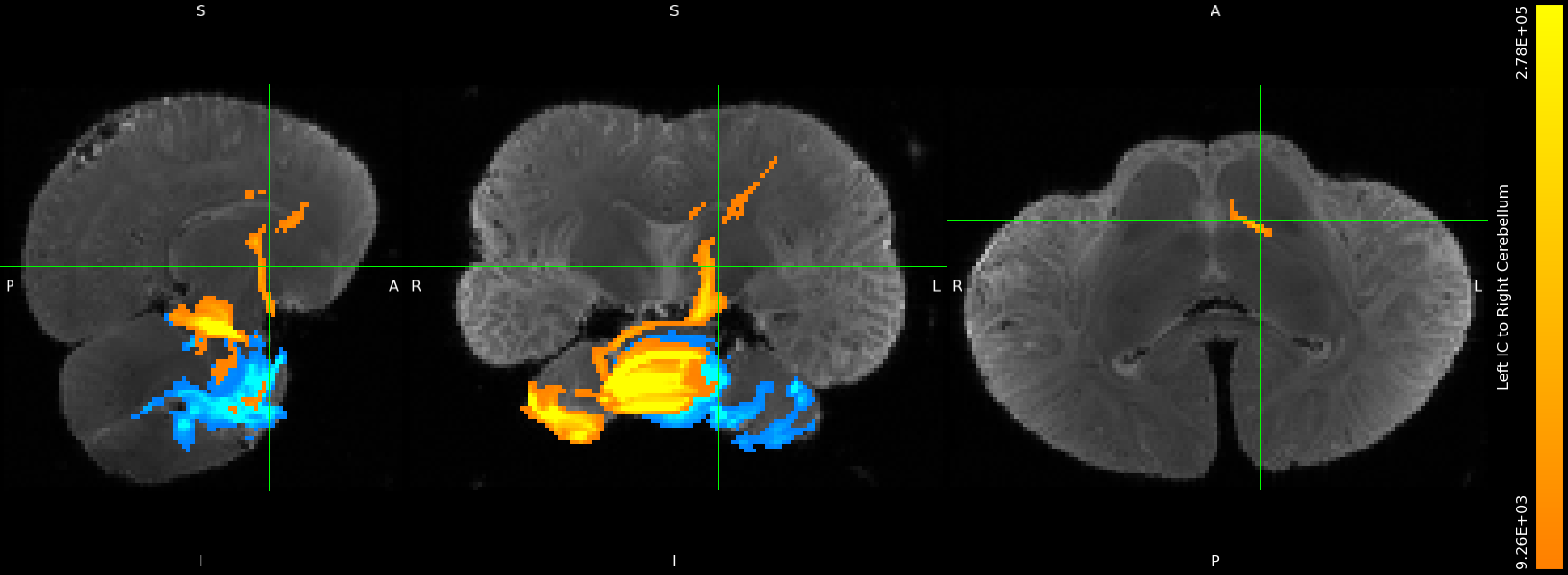

Supplement: S1 File — S1 Text. Detailed cerebellar and subcortical projection sites in IC-cerebellar traces. S2 Text. Detailed cortical projections in IC-cerebellar traces. S3 Figures. Masked regions of interest in FSLeyes. S3 Figure A. D. delphis. Red= right, blue=left for cerebella, yellow=right and turquoise=left for inferior colliculi. S3 Figure B. S. Attenuata. Red= right, blue=left for cerebella, yellow=right and turquoise=left for inferior colliculi. S3 Figure C. L. acutus. Red= right, blue=left for cerebella, yellow=right and turquoise=left for inferior colliculi. S3 Figure D. B. borealis. Red= right, blue=left for cerebella, yellow=right and turquoise=left for inferior colliculi. S4 Figures. Ascending auditory tractograms. S4 Figure A1: D. delphis, left IC tracts shown in blue, right IC tracts shown in red, minimum threshold set to 1% and maximum threshold set to 30% of waytotals. Orthographic view. S4 Figure A2: D. delphis, left IC tracts shown in blue, right IC tracts shown in red, set to a more liberal threshold of minimum 0.1% and maximum 5% of waytotals. Orthographic view. S4 Figure A3: D. delphis, left IC tracts shown in blue, right IC tracts shown in red, set to a more liberal threshold of minimum 0.1% and maximum 5% of waytotals. Still 3-dimensional view. S4 Figure A4: D. delphis, left IC tracts shown in blue, right IC tracts shown in red, set to a more liberal threshold of minimum 0.1% and maximum 5% of waytotals. Rotating 3-dimensional view. S4 Figure B1: S. attenuata, left IC tracts shown in blue, right IC tracts shown in red, minimum threshold set to 1% and maximum threshold set to 30% of waytotals. Orthographic view. S4 Figure B2: S. attenuata, left IC tracts shown in blue, right IC tracts shown in red, set to a more liberal threshold of minimum 0.1% and maximum 5% of waytotals. Orthographic view. S4 Figure B3: S. attenuata, left IC tracts shown in blue, right IC tracts shown in red, set to a more liberal threshold of minimum 0.1% and maximum 5% of waytotals. Still [file pone.0323617.s001.zip › supporting_information/s6_fig_a1.png]

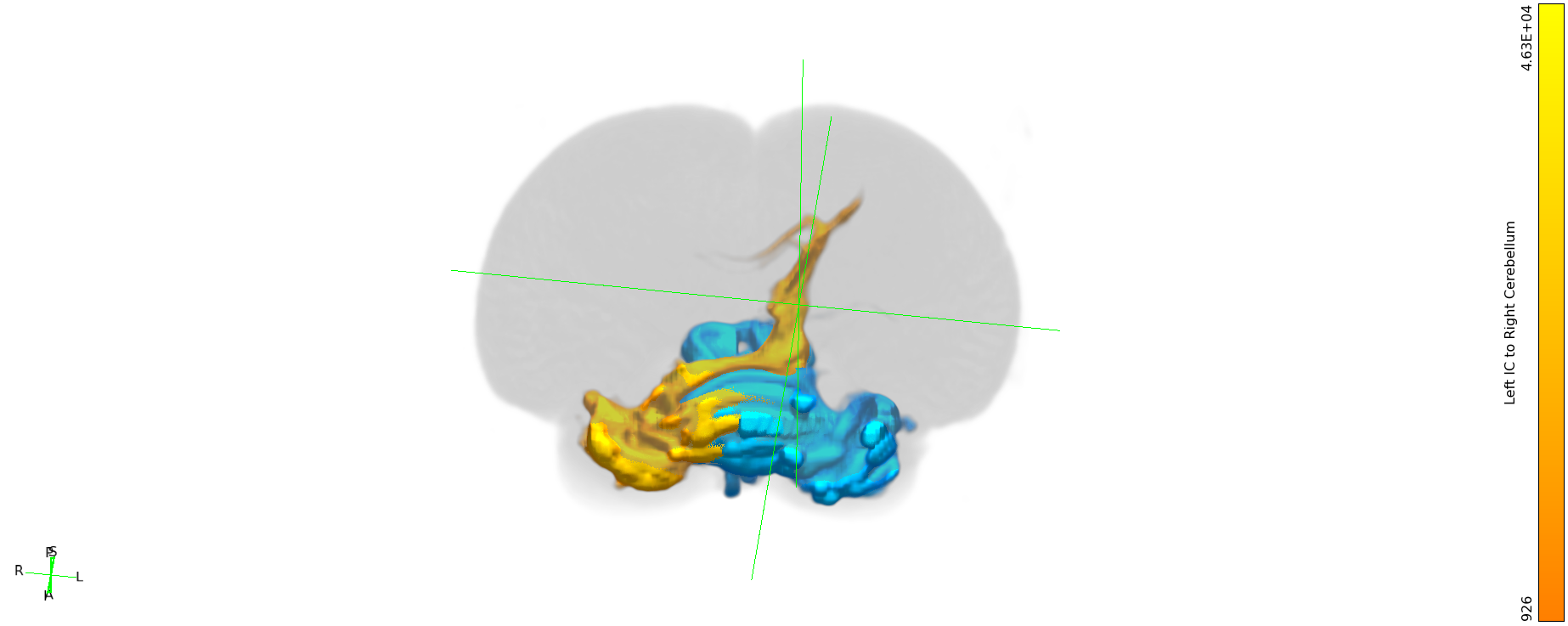

Supplement: S1 File — S1 Text. Detailed cerebellar and subcortical projection sites in IC-cerebellar traces. S2 Text. Detailed cortical projections in IC-cerebellar traces. S3 Figures. Masked regions of interest in FSLeyes. S3 Figure A. D. delphis. Red= right, blue=left for cerebella, yellow=right and turquoise=left for inferior colliculi. S3 Figure B. S. Attenuata. Red= right, blue=left for cerebella, yellow=right and turquoise=left for inferior colliculi. S3 Figure C. L. acutus. Red= right, blue=left for cerebella, yellow=right and turquoise=left for inferior colliculi. S3 Figure D. B. borealis. Red= right, blue=left for cerebella, yellow=right and turquoise=left for inferior colliculi. S4 Figures. Ascending auditory tractograms. S4 Figure A1: D. delphis, left IC tracts shown in blue, right IC tracts shown in red, minimum threshold set to 1% and maximum threshold set to 30% of waytotals. Orthographic view. S4 Figure A2: D. delphis, left IC tracts shown in blue, right IC tracts shown in red, set to a more liberal threshold of minimum 0.1% and maximum 5% of waytotals. Orthographic view. S4 Figure A3: D. delphis, left IC tracts shown in blue, right IC tracts shown in red, set to a more liberal threshold of minimum 0.1% and maximum 5% of waytotals. Still 3-dimensional view. S4 Figure A4: D. delphis, left IC tracts shown in blue, right IC tracts shown in red, set to a more liberal threshold of minimum 0.1% and maximum 5% of waytotals. Rotating 3-dimensional view. S4 Figure B1: S. attenuata, left IC tracts shown in blue, right IC tracts shown in red, minimum threshold set to 1% and maximum threshold set to 30% of waytotals. Orthographic view. S4 Figure B2: S. attenuata, left IC tracts shown in blue, right IC tracts shown in red, set to a more liberal threshold of minimum 0.1% and maximum 5% of waytotals. Orthographic view. S4 Figure B3: S. attenuata, left IC tracts shown in blue, right IC tracts shown in red, set to a more liberal threshold of minimum 0.1% and maximum 5% of waytotals. Still [file pone.0323617.s001.zip › supporting_information/s6_fig_a3.png]

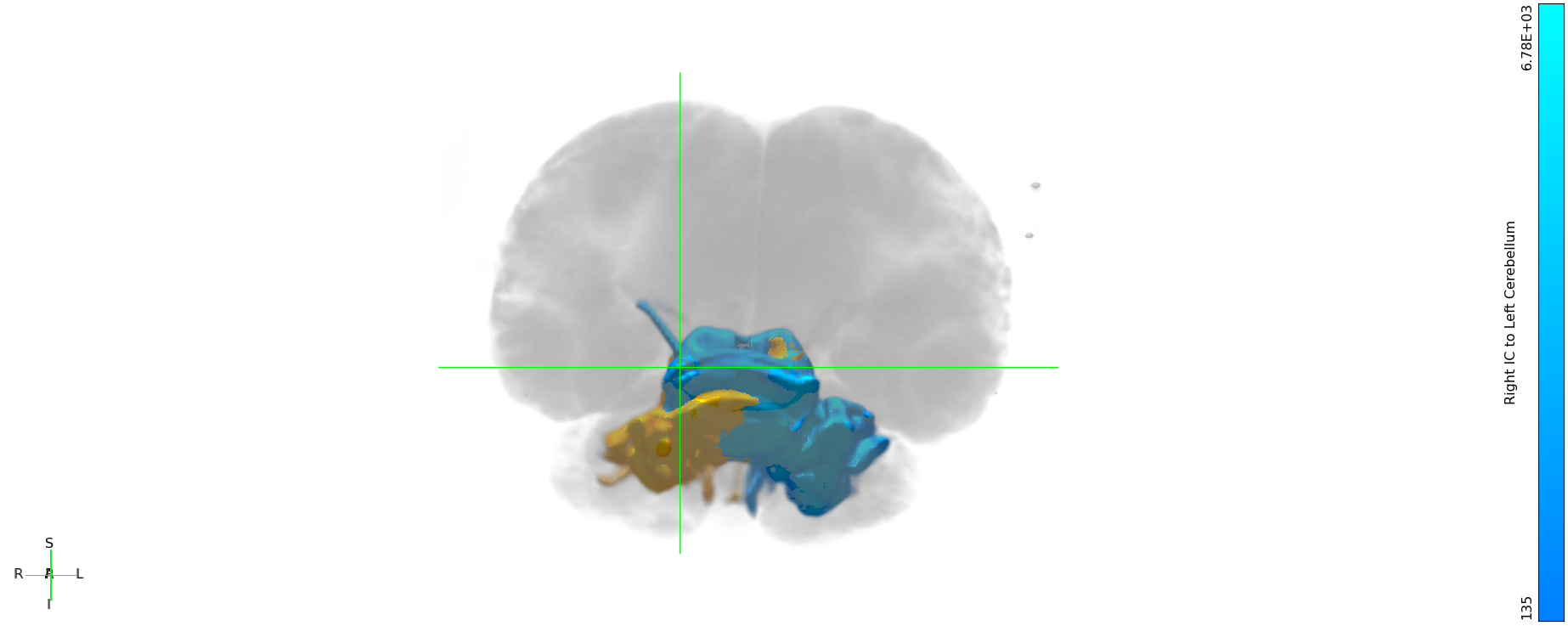

Supplement: S1 File — S1 Text. Detailed cerebellar and subcortical projection sites in IC-cerebellar traces. S2 Text. Detailed cortical projections in IC-cerebellar traces. S3 Figures. Masked regions of interest in FSLeyes. S3 Figure A. D. delphis. Red= right, blue=left for cerebella, yellow=right and turquoise=left for inferior colliculi. S3 Figure B. S. Attenuata. Red= right, blue=left for cerebella, yellow=right and turquoise=left for inferior colliculi. S3 Figure C. L. acutus. Red= right, blue=left for cerebella, yellow=right and turquoise=left for inferior colliculi. S3 Figure D. B. borealis. Red= right, blue=left for cerebella, yellow=right and turquoise=left for inferior colliculi. S4 Figures. Ascending auditory tractograms. S4 Figure A1: D. delphis, left IC tracts shown in blue, right IC tracts shown in red, minimum threshold set to 1% and maximum threshold set to 30% of waytotals. Orthographic view. S4 Figure A2: D. delphis, left IC tracts shown in blue, right IC tracts shown in red, set to a more liberal threshold of minimum 0.1% and maximum 5% of waytotals. Orthographic view. S4 Figure A3: D. delphis, left IC tracts shown in blue, right IC tracts shown in red, set to a more liberal threshold of minimum 0.1% and maximum 5% of waytotals. Still 3-dimensional view. S4 Figure A4: D. delphis, left IC tracts shown in blue, right IC tracts shown in red, set to a more liberal threshold of minimum 0.1% and maximum 5% of waytotals. Rotating 3-dimensional view. S4 Figure B1: S. attenuata, left IC tracts shown in blue, right IC tracts shown in red, minimum threshold set to 1% and maximum threshold set to 30% of waytotals. Orthographic view. S4 Figure B2: S. attenuata, left IC tracts shown in blue, right IC tracts shown in red, set to a more liberal threshold of minimum 0.1% and maximum 5% of waytotals. Orthographic view. S4 Figure B3: S. attenuata, left IC tracts shown in blue, right IC tracts shown in red, set to a more liberal threshold of minimum 0.1% and maximum 5% of waytotals. Still [file pone.0323617.s001.zip › supporting_information/s6_fig_b3.png]

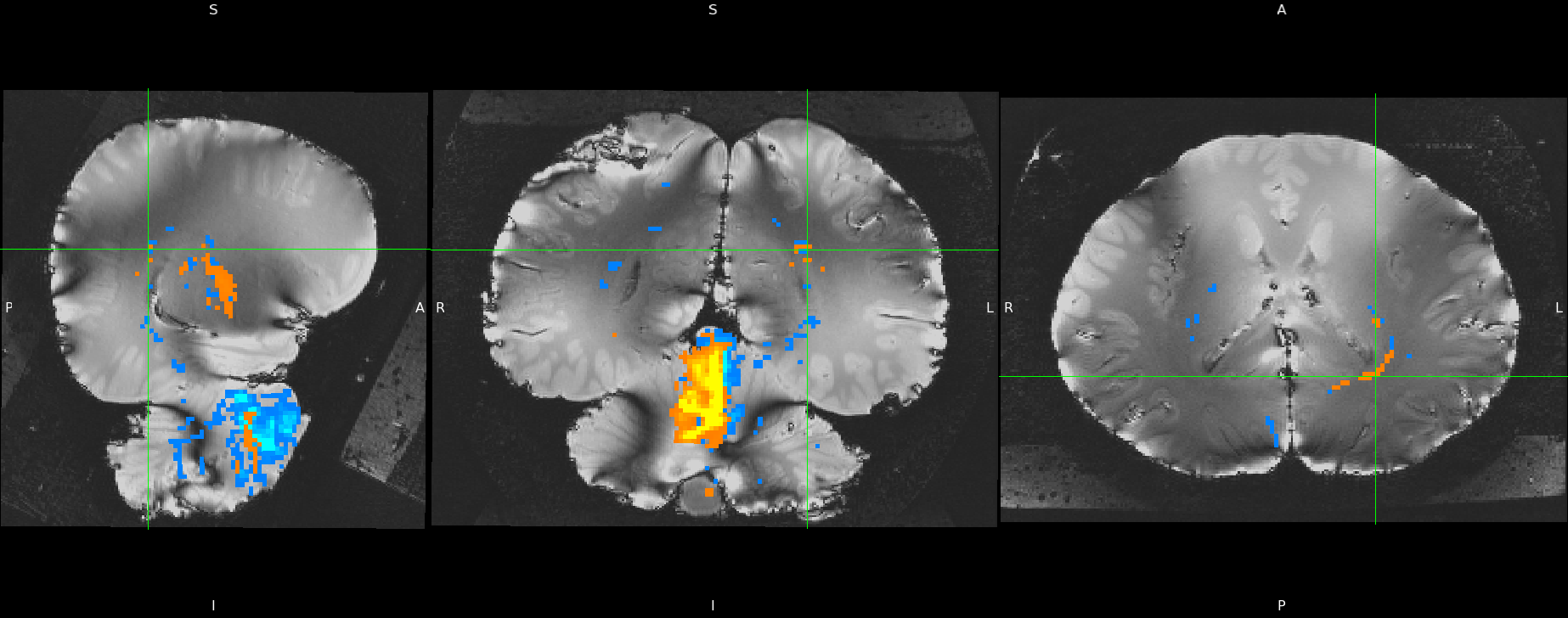

Supplement: S1 File — S1 Text. Detailed cerebellar and subcortical projection sites in IC-cerebellar traces. S2 Text. Detailed cortical projections in IC-cerebellar traces. S3 Figures. Masked regions of interest in FSLeyes. S3 Figure A. D. delphis. Red= right, blue=left for cerebella, yellow=right and turquoise=left for inferior colliculi. S3 Figure B. S. Attenuata. Red= right, blue=left for cerebella, yellow=right and turquoise=left for inferior colliculi. S3 Figure C. L. acutus. Red= right, blue=left for cerebella, yellow=right and turquoise=left for inferior colliculi. S3 Figure D. B. borealis. Red= right, blue=left for cerebella, yellow=right and turquoise=left for inferior colliculi. S4 Figures. Ascending auditory tractograms. S4 Figure A1: D. delphis, left IC tracts shown in blue, right IC tracts shown in red, minimum threshold set to 1% and maximum threshold set to 30% of waytotals. Orthographic view. S4 Figure A2: D. delphis, left IC tracts shown in blue, right IC tracts shown in red, set to a more liberal threshold of minimum 0.1% and maximum 5% of waytotals. Orthographic view. S4 Figure A3: D. delphis, left IC tracts shown in blue, right IC tracts shown in red, set to a more liberal threshold of minimum 0.1% and maximum 5% of waytotals. Still 3-dimensional view. S4 Figure A4: D. delphis, left IC tracts shown in blue, right IC tracts shown in red, set to a more liberal threshold of minimum 0.1% and maximum 5% of waytotals. Rotating 3-dimensional view. S4 Figure B1: S. attenuata, left IC tracts shown in blue, right IC tracts shown in red, minimum threshold set to 1% and maximum threshold set to 30% of waytotals. Orthographic view. S4 Figure B2: S. attenuata, left IC tracts shown in blue, right IC tracts shown in red, set to a more liberal threshold of minimum 0.1% and maximum 5% of waytotals. Orthographic view. S4 Figure B3: S. attenuata, left IC tracts shown in blue, right IC tracts shown in red, set to a more liberal threshold of minimum 0.1% and maximum 5% of waytotals. Still [file pone.0323617.s001.zip › supporting_information/s6_fig_b2.png]

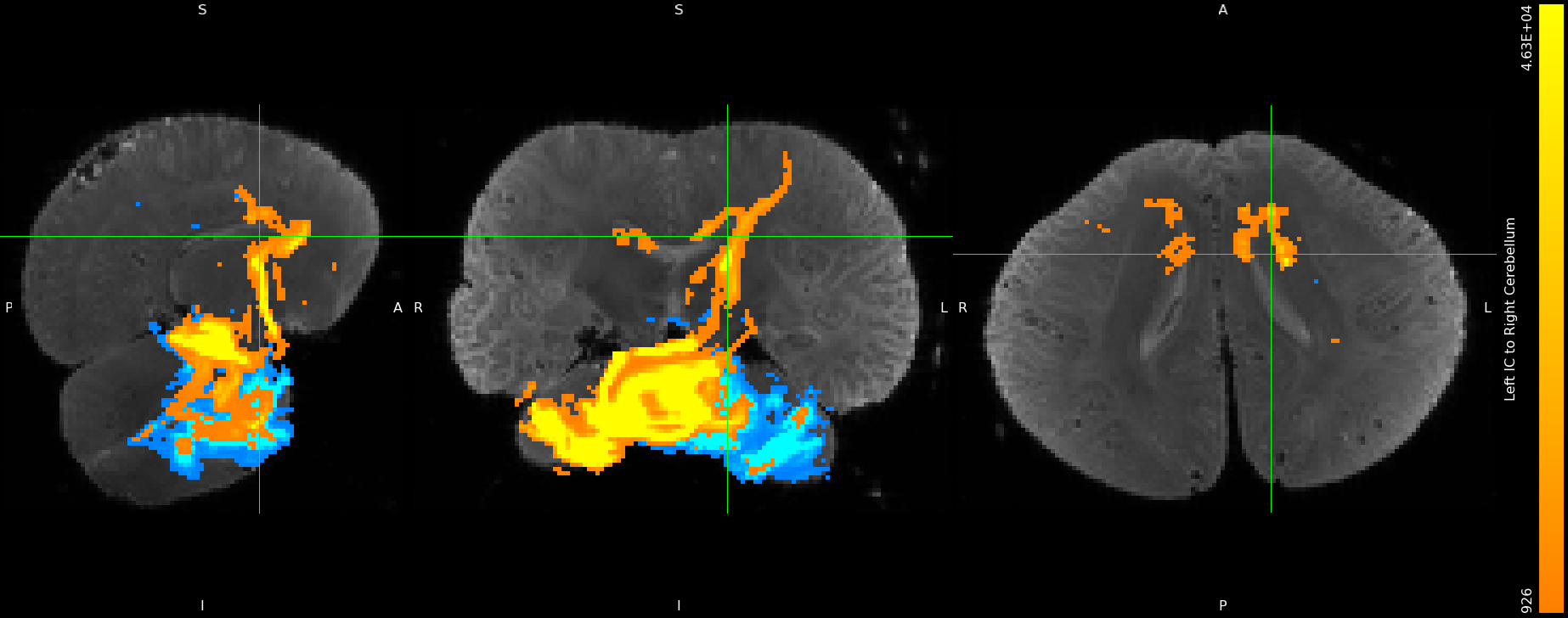

Supplement: S1 File — S1 Text. Detailed cerebellar and subcortical projection sites in IC-cerebellar traces. S2 Text. Detailed cortical projections in IC-cerebellar traces. S3 Figures. Masked regions of interest in FSLeyes. S3 Figure A. D. delphis. Red= right, blue=left for cerebella, yellow=right and turquoise=left for inferior colliculi. S3 Figure B. S. Attenuata. Red= right, blue=left for cerebella, yellow=right and turquoise=left for inferior colliculi. S3 Figure C. L. acutus. Red= right, blue=left for cerebella, yellow=right and turquoise=left for inferior colliculi. S3 Figure D. B. borealis. Red= right, blue=left for cerebella, yellow=right and turquoise=left for inferior colliculi. S4 Figures. Ascending auditory tractograms. S4 Figure A1: D. delphis, left IC tracts shown in blue, right IC tracts shown in red, minimum threshold set to 1% and maximum threshold set to 30% of waytotals. Orthographic view. S4 Figure A2: D. delphis, left IC tracts shown in blue, right IC tracts shown in red, set to a more liberal threshold of minimum 0.1% and maximum 5% of waytotals. Orthographic view. S4 Figure A3: D. delphis, left IC tracts shown in blue, right IC tracts shown in red, set to a more liberal threshold of minimum 0.1% and maximum 5% of waytotals. Still 3-dimensional view. S4 Figure A4: D. delphis, left IC tracts shown in blue, right IC tracts shown in red, set to a more liberal threshold of minimum 0.1% and maximum 5% of waytotals. Rotating 3-dimensional view. S4 Figure B1: S. attenuata, left IC tracts shown in blue, right IC tracts shown in red, minimum threshold set to 1% and maximum threshold set to 30% of waytotals. Orthographic view. S4 Figure B2: S. attenuata, left IC tracts shown in blue, right IC tracts shown in red, set to a more liberal threshold of minimum 0.1% and maximum 5% of waytotals. Orthographic view. S4 Figure B3: S. attenuata, left IC tracts shown in blue, right IC tracts shown in red, set to a more liberal threshold of minimum 0.1% and maximum 5% of waytotals. Still [file pone.0323617.s001.zip › supporting_information/s6_fig_a2.png]

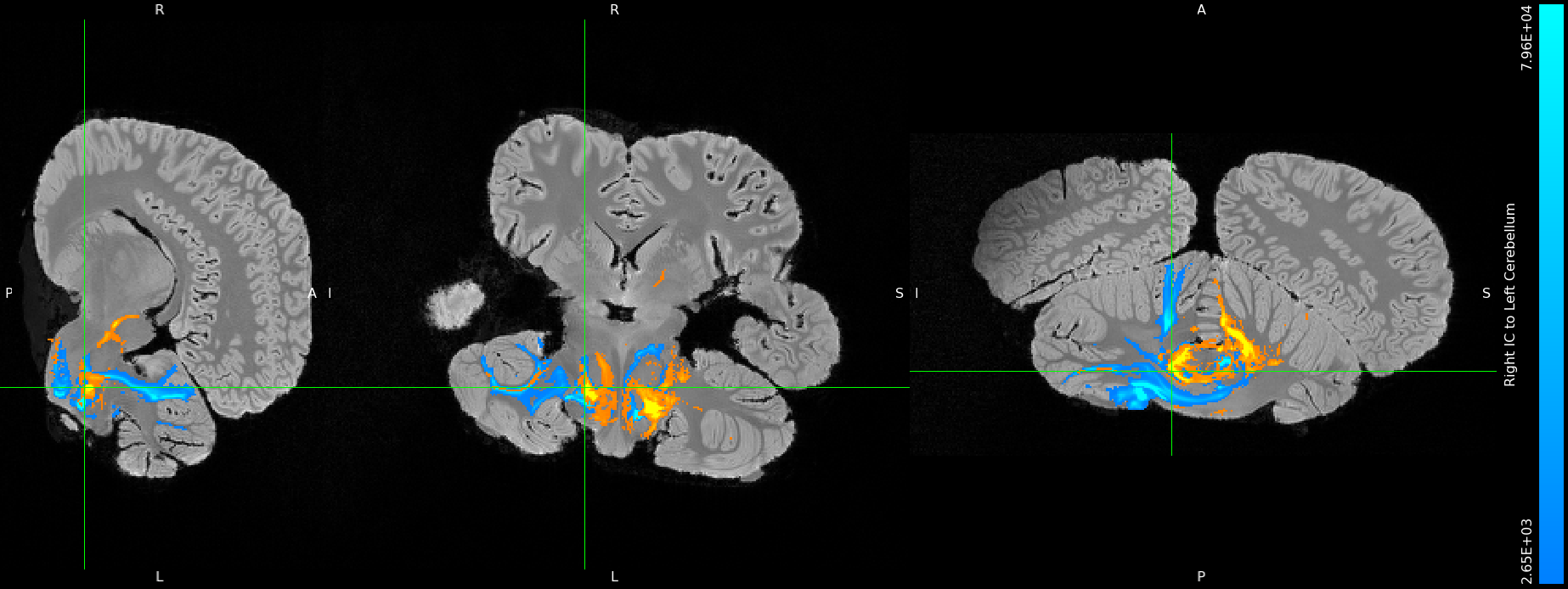

Supplement: S1 File — S1 Text. Detailed cerebellar and subcortical projection sites in IC-cerebellar traces. S2 Text. Detailed cortical projections in IC-cerebellar traces. S3 Figures. Masked regions of interest in FSLeyes. S3 Figure A. D. delphis. Red= right, blue=left for cerebella, yellow=right and turquoise=left for inferior colliculi. S3 Figure B. S. Attenuata. Red= right, blue=left for cerebella, yellow=right and turquoise=left for inferior colliculi. S3 Figure C. L. acutus. Red= right, blue=left for cerebella, yellow=right and turquoise=left for inferior colliculi. S3 Figure D. B. borealis. Red= right, blue=left for cerebella, yellow=right and turquoise=left for inferior colliculi. S4 Figures. Ascending auditory tractograms. S4 Figure A1: D. delphis, left IC tracts shown in blue, right IC tracts shown in red, minimum threshold set to 1% and maximum threshold set to 30% of waytotals. Orthographic view. S4 Figure A2: D. delphis, left IC tracts shown in blue, right IC tracts shown in red, set to a more liberal threshold of minimum 0.1% and maximum 5% of waytotals. Orthographic view. S4 Figure A3: D. delphis, left IC tracts shown in blue, right IC tracts shown in red, set to a more liberal threshold of minimum 0.1% and maximum 5% of waytotals. Still 3-dimensional view. S4 Figure A4: D. delphis, left IC tracts shown in blue, right IC tracts shown in red, set to a more liberal threshold of minimum 0.1% and maximum 5% of waytotals. Rotating 3-dimensional view. S4 Figure B1: S. attenuata, left IC tracts shown in blue, right IC tracts shown in red, minimum threshold set to 1% and maximum threshold set to 30% of waytotals. Orthographic view. S4 Figure B2: S. attenuata, left IC tracts shown in blue, right IC tracts shown in red, set to a more liberal threshold of minimum 0.1% and maximum 5% of waytotals. Orthographic view. S4 Figure B3: S. attenuata, left IC tracts shown in blue, right IC tracts shown in red, set to a more liberal threshold of minimum 0.1% and maximum 5% of waytotals. Still [file pone.0323617.s001.zip › supporting_information/s6_fig_d1.png]

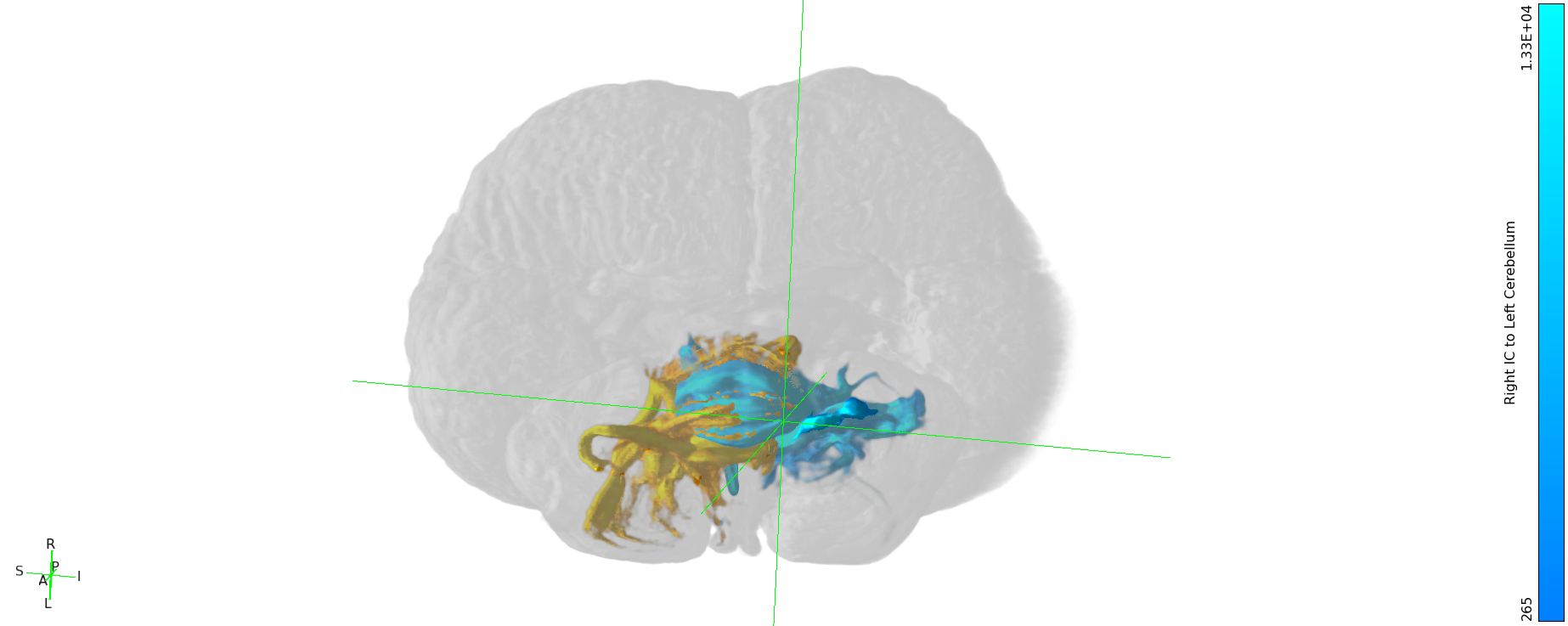

Supplement: S1 File — S1 Text. Detailed cerebellar and subcortical projection sites in IC-cerebellar traces. S2 Text. Detailed cortical projections in IC-cerebellar traces. S3 Figures. Masked regions of interest in FSLeyes. S3 Figure A. D. delphis. Red= right, blue=left for cerebella, yellow=right and turquoise=left for inferior colliculi. S3 Figure B. S. Attenuata. Red= right, blue=left for cerebella, yellow=right and turquoise=left for inferior colliculi. S3 Figure C. L. acutus. Red= right, blue=left for cerebella, yellow=right and turquoise=left for inferior colliculi. S3 Figure D. B. borealis. Red= right, blue=left for cerebella, yellow=right and turquoise=left for inferior colliculi. S4 Figures. Ascending auditory tractograms. S4 Figure A1: D. delphis, left IC tracts shown in blue, right IC tracts shown in red, minimum threshold set to 1% and maximum threshold set to 30% of waytotals. Orthographic view. S4 Figure A2: D. delphis, left IC tracts shown in blue, right IC tracts shown in red, set to a more liberal threshold of minimum 0.1% and maximum 5% of waytotals. Orthographic view. S4 Figure A3: D. delphis, left IC tracts shown in blue, right IC tracts shown in red, set to a more liberal threshold of minimum 0.1% and maximum 5% of waytotals. Still 3-dimensional view. S4 Figure A4: D. delphis, left IC tracts shown in blue, right IC tracts shown in red, set to a more liberal threshold of minimum 0.1% and maximum 5% of waytotals. Rotating 3-dimensional view. S4 Figure B1: S. attenuata, left IC tracts shown in blue, right IC tracts shown in red, minimum threshold set to 1% and maximum threshold set to 30% of waytotals. Orthographic view. S4 Figure B2: S. attenuata, left IC tracts shown in blue, right IC tracts shown in red, set to a more liberal threshold of minimum 0.1% and maximum 5% of waytotals. Orthographic view. S4 Figure B3: S. attenuata, left IC tracts shown in blue, right IC tracts shown in red, set to a more liberal threshold of minimum 0.1% and maximum 5% of waytotals. Still [file pone.0323617.s001.zip › supporting_information/s6_fig_d3.png]

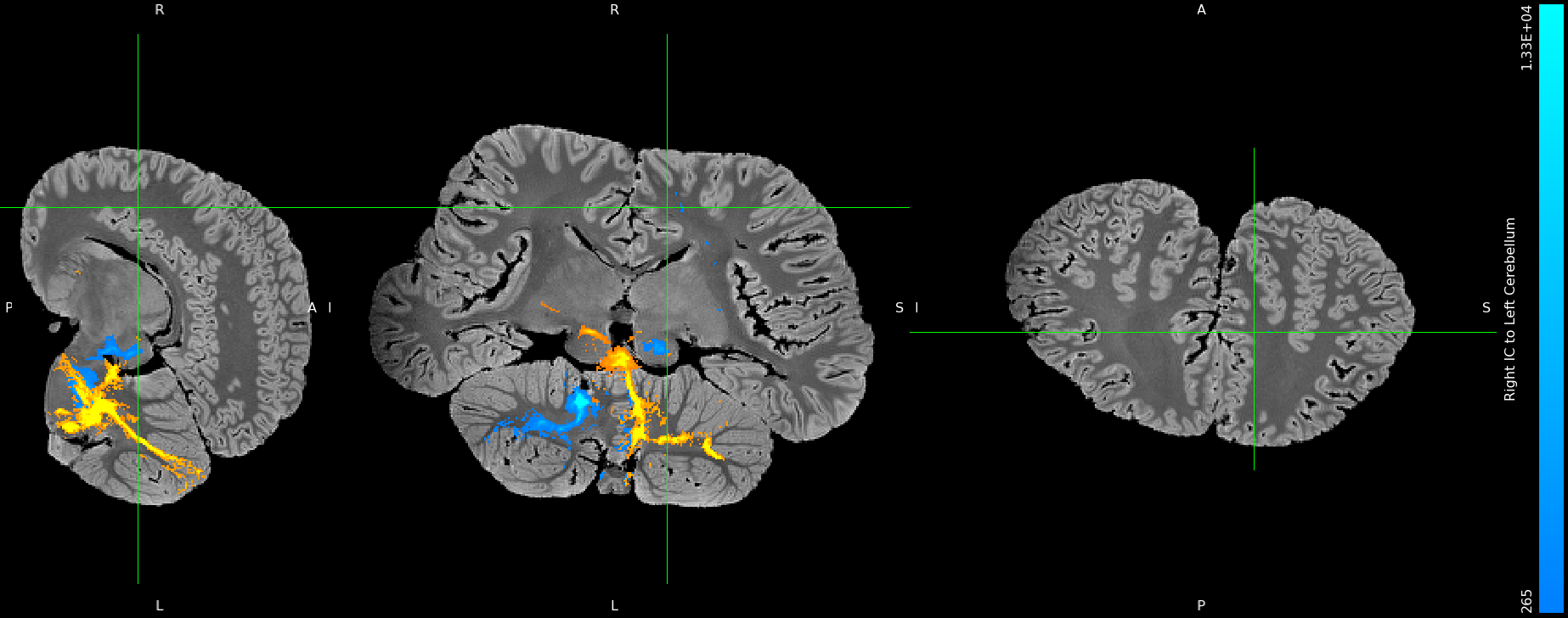

Supplement: S1 File — S1 Text. Detailed cerebellar and subcortical projection sites in IC-cerebellar traces. S2 Text. Detailed cortical projections in IC-cerebellar traces. S3 Figures. Masked regions of interest in FSLeyes. S3 Figure A. D. delphis. Red= right, blue=left for cerebella, yellow=right and turquoise=left for inferior colliculi. S3 Figure B. S. Attenuata. Red= right, blue=left for cerebella, yellow=right and turquoise=left for inferior colliculi. S3 Figure C. L. acutus. Red= right, blue=left for cerebella, yellow=right and turquoise=left for inferior colliculi. S3 Figure D. B. borealis. Red= right, blue=left for cerebella, yellow=right and turquoise=left for inferior colliculi. S4 Figures. Ascending auditory tractograms. S4 Figure A1: D. delphis, left IC tracts shown in blue, right IC tracts shown in red, minimum threshold set to 1% and maximum threshold set to 30% of waytotals. Orthographic view. S4 Figure A2: D. delphis, left IC tracts shown in blue, right IC tracts shown in red, set to a more liberal threshold of minimum 0.1% and maximum 5% of waytotals. Orthographic view. S4 Figure A3: D. delphis, left IC tracts shown in blue, right IC tracts shown in red, set to a more liberal threshold of minimum 0.1% and maximum 5% of waytotals. Still 3-dimensional view. S4 Figure A4: D. delphis, left IC tracts shown in blue, right IC tracts shown in red, set to a more liberal threshold of minimum 0.1% and maximum 5% of waytotals. Rotating 3-dimensional view. S4 Figure B1: S. attenuata, left IC tracts shown in blue, right IC tracts shown in red, minimum threshold set to 1% and maximum threshold set to 30% of waytotals. Orthographic view. S4 Figure B2: S. attenuata, left IC tracts shown in blue, right IC tracts shown in red, set to a more liberal threshold of minimum 0.1% and maximum 5% of waytotals. Orthographic view. S4 Figure B3: S. attenuata, left IC tracts shown in blue, right IC tracts shown in red, set to a more liberal threshold of minimum 0.1% and maximum 5% of waytotals. Still [file pone.0323617.s001.zip › supporting_information/s6_fig_d2.png]

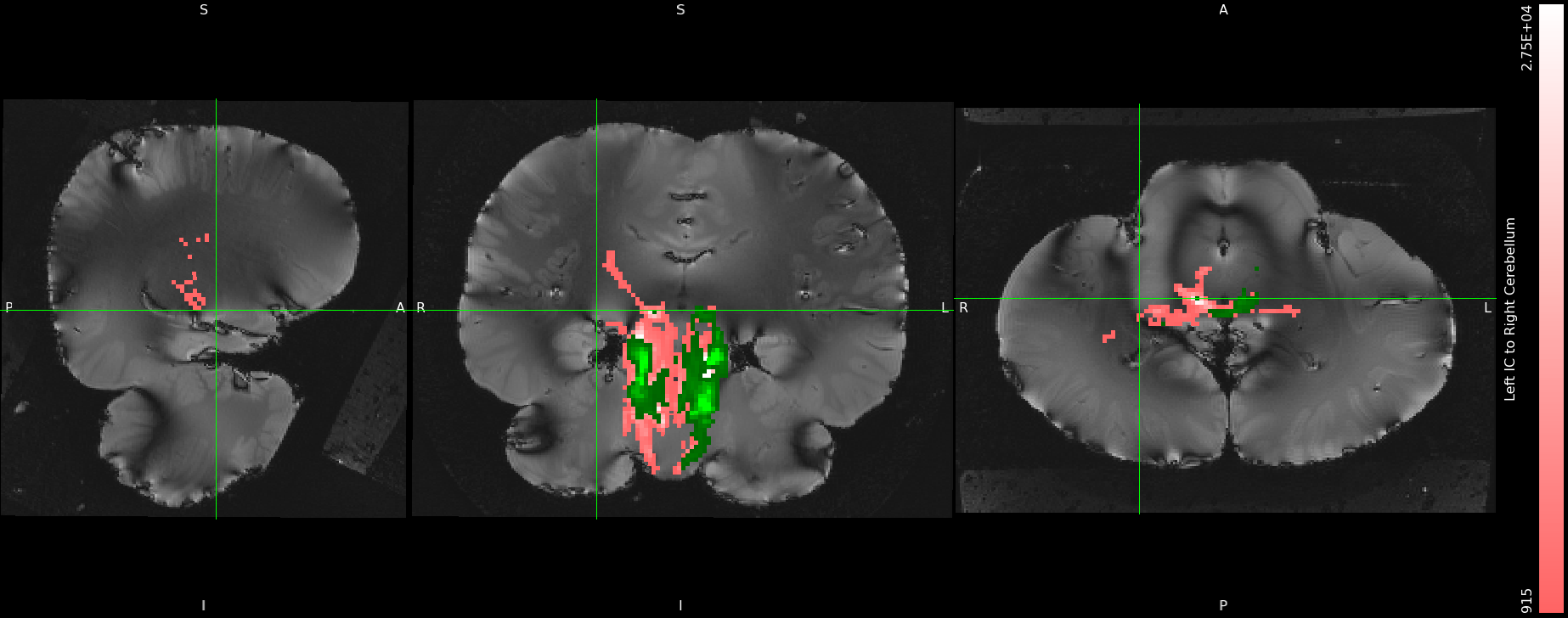

Supplement: S1 File — S1 Text. Detailed cerebellar and subcortical projection sites in IC-cerebellar traces. S2 Text. Detailed cortical projections in IC-cerebellar traces. S3 Figures. Masked regions of interest in FSLeyes. S3 Figure A. D. delphis. Red= right, blue=left for cerebella, yellow=right and turquoise=left for inferior colliculi. S3 Figure B. S. Attenuata. Red= right, blue=left for cerebella, yellow=right and turquoise=left for inferior colliculi. S3 Figure C. L. acutus. Red= right, blue=left for cerebella, yellow=right and turquoise=left for inferior colliculi. S3 Figure D. B. borealis. Red= right, blue=left for cerebella, yellow=right and turquoise=left for inferior colliculi. S4 Figures. Ascending auditory tractograms. S4 Figure A1: D. delphis, left IC tracts shown in blue, right IC tracts shown in red, minimum threshold set to 1% and maximum threshold set to 30% of waytotals. Orthographic view. S4 Figure A2: D. delphis, left IC tracts shown in blue, right IC tracts shown in red, set to a more liberal threshold of minimum 0.1% and maximum 5% of waytotals. Orthographic view. S4 Figure A3: D. delphis, left IC tracts shown in blue, right IC tracts shown in red, set to a more liberal threshold of minimum 0.1% and maximum 5% of waytotals. Still 3-dimensional view. S4 Figure A4: D. delphis, left IC tracts shown in blue, right IC tracts shown in red, set to a more liberal threshold of minimum 0.1% and maximum 5% of waytotals. Rotating 3-dimensional view. S4 Figure B1: S. attenuata, left IC tracts shown in blue, right IC tracts shown in red, minimum threshold set to 1% and maximum threshold set to 30% of waytotals. Orthographic view. S4 Figure B2: S. attenuata, left IC tracts shown in blue, right IC tracts shown in red, set to a more liberal threshold of minimum 0.1% and maximum 5% of waytotals. Orthographic view. S4 Figure B3: S. attenuata, left IC tracts shown in blue, right IC tracts shown in red, set to a more liberal threshold of minimum 0.1% and maximum 5% of waytotals. Still [file pone.0323617.s001.zip › supporting_information/s5_fig_b1.png]

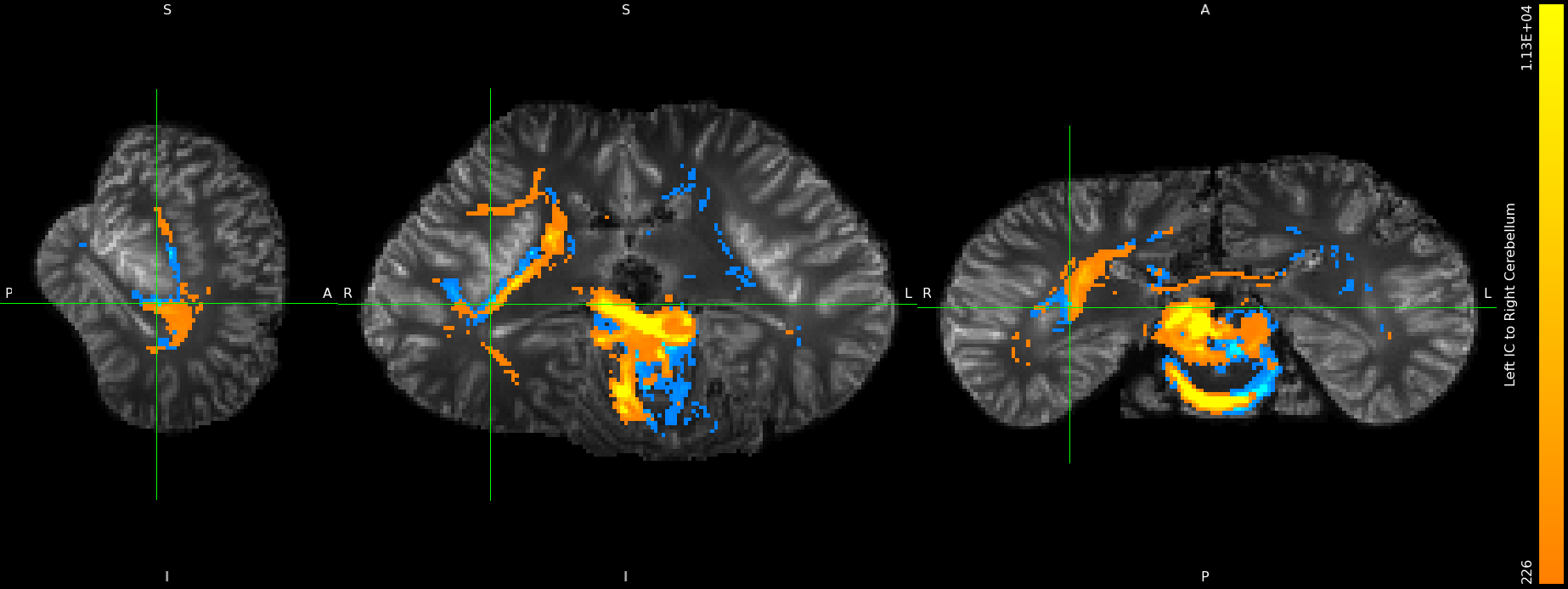

Supplement: S1 File — S1 Text. Detailed cerebellar and subcortical projection sites in IC-cerebellar traces. S2 Text. Detailed cortical projections in IC-cerebellar traces. S3 Figures. Masked regions of interest in FSLeyes. S3 Figure A. D. delphis. Red= right, blue=left for cerebella, yellow=right and turquoise=left for inferior colliculi. S3 Figure B. S. Attenuata. Red= right, blue=left for cerebella, yellow=right and turquoise=left for inferior colliculi. S3 Figure C. L. acutus. Red= right, blue=left for cerebella, yellow=right and turquoise=left for inferior colliculi. S3 Figure D. B. borealis. Red= right, blue=left for cerebella, yellow=right and turquoise=left for inferior colliculi. S4 Figures. Ascending auditory tractograms. S4 Figure A1: D. delphis, left IC tracts shown in blue, right IC tracts shown in red, minimum threshold set to 1% and maximum threshold set to 30% of waytotals. Orthographic view. S4 Figure A2: D. delphis, left IC tracts shown in blue, right IC tracts shown in red, set to a more liberal threshold of minimum 0.1% and maximum 5% of waytotals. Orthographic view. S4 Figure A3: D. delphis, left IC tracts shown in blue, right IC tracts shown in red, set to a more liberal threshold of minimum 0.1% and maximum 5% of waytotals. Still 3-dimensional view. S4 Figure A4: D. delphis, left IC tracts shown in blue, right IC tracts shown in red, set to a more liberal threshold of minimum 0.1% and maximum 5% of waytotals. Rotating 3-dimensional view. S4 Figure B1: S. attenuata, left IC tracts shown in blue, right IC tracts shown in red, minimum threshold set to 1% and maximum threshold set to 30% of waytotals. Orthographic view. S4 Figure B2: S. attenuata, left IC tracts shown in blue, right IC tracts shown in red, set to a more liberal threshold of minimum 0.1% and maximum 5% of waytotals. Orthographic view. S4 Figure B3: S. attenuata, left IC tracts shown in blue, right IC tracts shown in red, set to a more liberal threshold of minimum 0.1% and maximum 5% of waytotals. Still [file pone.0323617.s001.zip › supporting_information/s6_fig_c2.png]

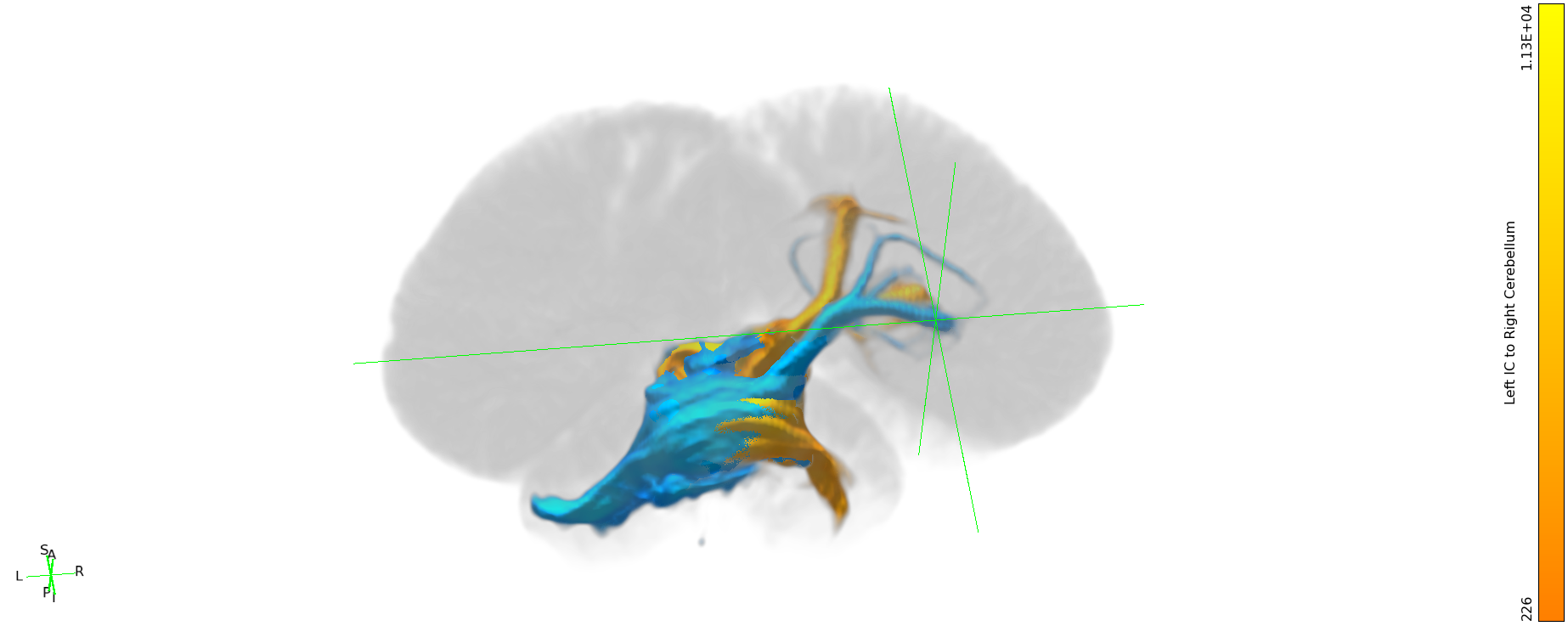

Supplement: S1 File — S1 Text. Detailed cerebellar and subcortical projection sites in IC-cerebellar traces. S2 Text. Detailed cortical projections in IC-cerebellar traces. S3 Figures. Masked regions of interest in FSLeyes. S3 Figure A. D. delphis. Red= right, blue=left for cerebella, yellow=right and turquoise=left for inferior colliculi. S3 Figure B. S. Attenuata. Red= right, blue=left for cerebella, yellow=right and turquoise=left for inferior colliculi. S3 Figure C. L. acutus. Red= right, blue=left for cerebella, yellow=right and turquoise=left for inferior colliculi. S3 Figure D. B. borealis. Red= right, blue=left for cerebella, yellow=right and turquoise=left for inferior colliculi. S4 Figures. Ascending auditory tractograms. S4 Figure A1: D. delphis, left IC tracts shown in blue, right IC tracts shown in red, minimum threshold set to 1% and maximum threshold set to 30% of waytotals. Orthographic view. S4 Figure A2: D. delphis, left IC tracts shown in blue, right IC tracts shown in red, set to a more liberal threshold of minimum 0.1% and maximum 5% of waytotals. Orthographic view. S4 Figure A3: D. delphis, left IC tracts shown in blue, right IC tracts shown in red, set to a more liberal threshold of minimum 0.1% and maximum 5% of waytotals. Still 3-dimensional view. S4 Figure A4: D. delphis, left IC tracts shown in blue, right IC tracts shown in red, set to a more liberal threshold of minimum 0.1% and maximum 5% of waytotals. Rotating 3-dimensional view. S4 Figure B1: S. attenuata, left IC tracts shown in blue, right IC tracts shown in red, minimum threshold set to 1% and maximum threshold set to 30% of waytotals. Orthographic view. S4 Figure B2: S. attenuata, left IC tracts shown in blue, right IC tracts shown in red, set to a more liberal threshold of minimum 0.1% and maximum 5% of waytotals. Orthographic view. S4 Figure B3: S. attenuata, left IC tracts shown in blue, right IC tracts shown in red, set to a more liberal threshold of minimum 0.1% and maximum 5% of waytotals. Still [file pone.0323617.s001.zip › supporting_information/s6_fig_c3.png]

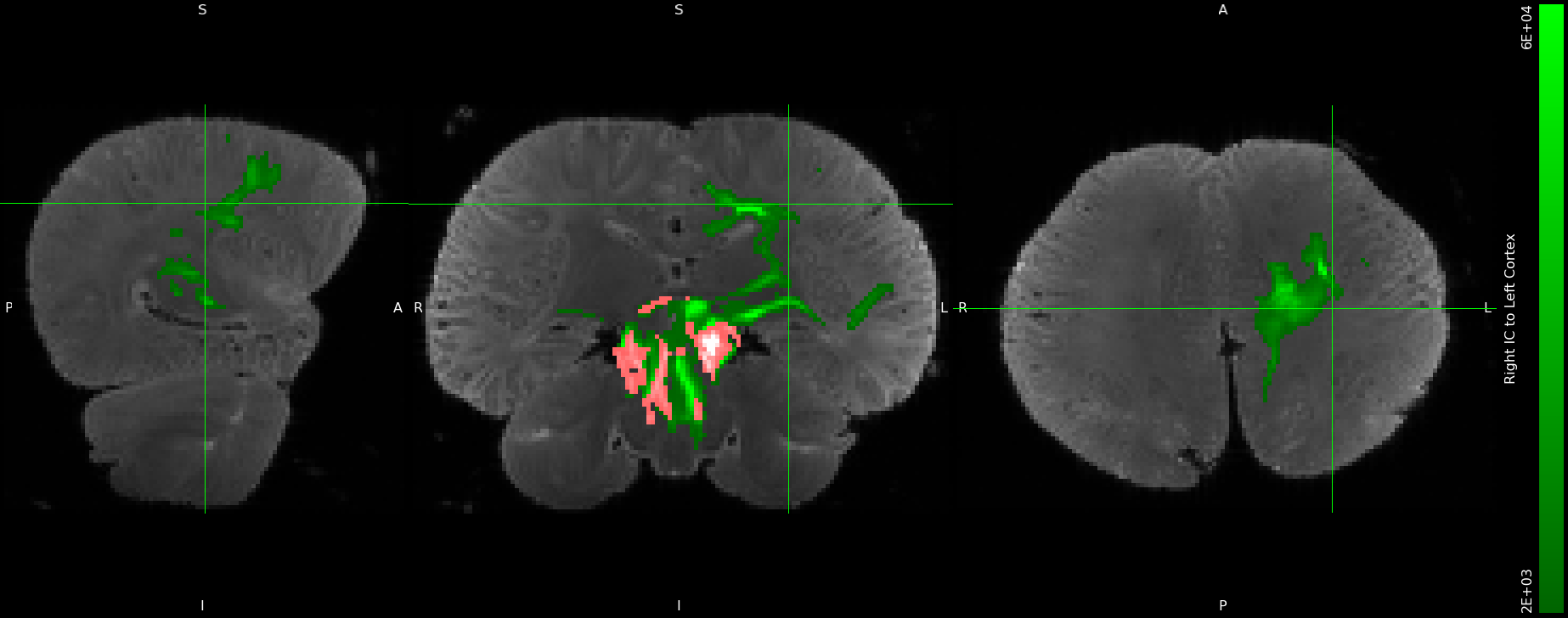

Supplement: S1 File — S1 Text. Detailed cerebellar and subcortical projection sites in IC-cerebellar traces. S2 Text. Detailed cortical projections in IC-cerebellar traces. S3 Figures. Masked regions of interest in FSLeyes. S3 Figure A. D. delphis. Red= right, blue=left for cerebella, yellow=right and turquoise=left for inferior colliculi. S3 Figure B. S. Attenuata. Red= right, blue=left for cerebella, yellow=right and turquoise=left for inferior colliculi. S3 Figure C. L. acutus. Red= right, blue=left for cerebella, yellow=right and turquoise=left for inferior colliculi. S3 Figure D. B. borealis. Red= right, blue=left for cerebella, yellow=right and turquoise=left for inferior colliculi. S4 Figures. Ascending auditory tractograms. S4 Figure A1: D. delphis, left IC tracts shown in blue, right IC tracts shown in red, minimum threshold set to 1% and maximum threshold set to 30% of waytotals. Orthographic view. S4 Figure A2: D. delphis, left IC tracts shown in blue, right IC tracts shown in red, set to a more liberal threshold of minimum 0.1% and maximum 5% of waytotals. Orthographic view. S4 Figure A3: D. delphis, left IC tracts shown in blue, right IC tracts shown in red, set to a more liberal threshold of minimum 0.1% and maximum 5% of waytotals. Still 3-dimensional view. S4 Figure A4: D. delphis, left IC tracts shown in blue, right IC tracts shown in red, set to a more liberal threshold of minimum 0.1% and maximum 5% of waytotals. Rotating 3-dimensional view. S4 Figure B1: S. attenuata, left IC tracts shown in blue, right IC tracts shown in red, minimum threshold set to 1% and maximum threshold set to 30% of waytotals. Orthographic view. S4 Figure B2: S. attenuata, left IC tracts shown in blue, right IC tracts shown in red, set to a more liberal threshold of minimum 0.1% and maximum 5% of waytotals. Orthographic view. S4 Figure B3: S. attenuata, left IC tracts shown in blue, right IC tracts shown in red, set to a more liberal threshold of minimum 0.1% and maximum 5% of waytotals. Still [file pone.0323617.s001.zip › supporting_information/s5_fig_a1.png]

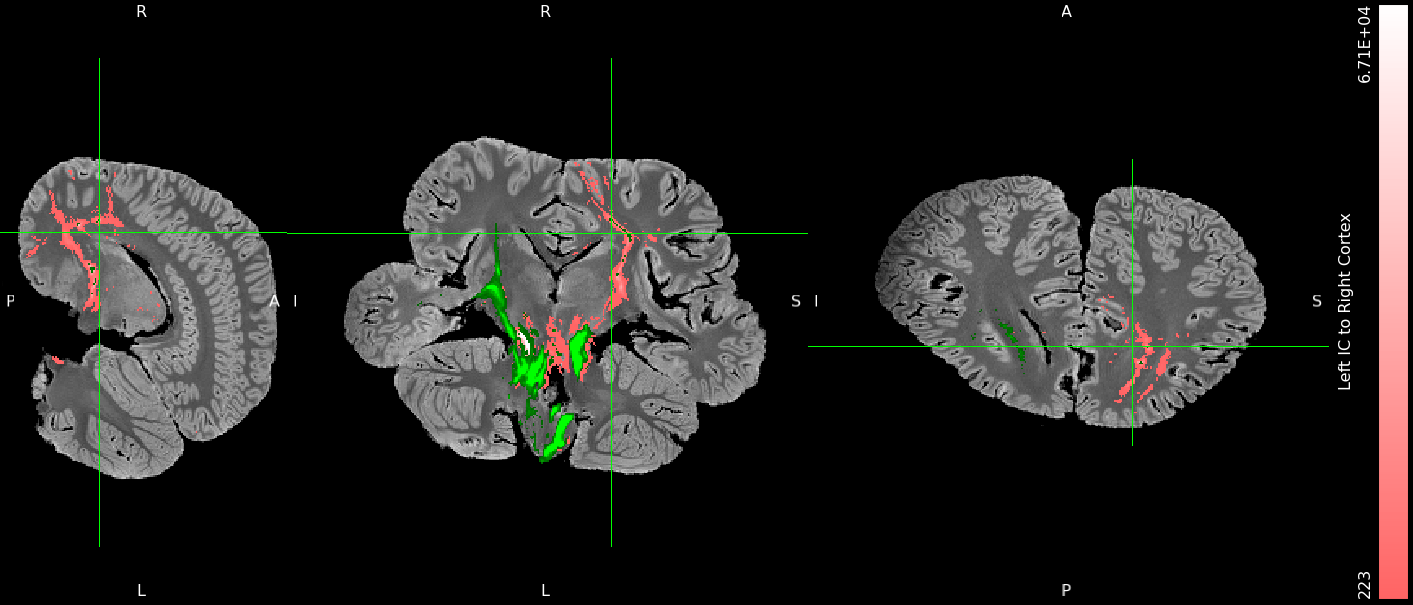

Supplement: S1 File — S1 Text. Detailed cerebellar and subcortical projection sites in IC-cerebellar traces. S2 Text. Detailed cortical projections in IC-cerebellar traces. S3 Figures. Masked regions of interest in FSLeyes. S3 Figure A. D. delphis. Red= right, blue=left for cerebella, yellow=right and turquoise=left for inferior colliculi. S3 Figure B. S. Attenuata. Red= right, blue=left for cerebella, yellow=right and turquoise=left for inferior colliculi. S3 Figure C. L. acutus. Red= right, blue=left for cerebella, yellow=right and turquoise=left for inferior colliculi. S3 Figure D. B. borealis. Red= right, blue=left for cerebella, yellow=right and turquoise=left for inferior colliculi. S4 Figures. Ascending auditory tractograms. S4 Figure A1: D. delphis, left IC tracts shown in blue, right IC tracts shown in red, minimum threshold set to 1% and maximum threshold set to 30% of waytotals. Orthographic view. S4 Figure A2: D. delphis, left IC tracts shown in blue, right IC tracts shown in red, set to a more liberal threshold of minimum 0.1% and maximum 5% of waytotals. Orthographic view. S4 Figure A3: D. delphis, left IC tracts shown in blue, right IC tracts shown in red, set to a more liberal threshold of minimum 0.1% and maximum 5% of waytotals. Still 3-dimensional view. S4 Figure A4: D. delphis, left IC tracts shown in blue, right IC tracts shown in red, set to a more liberal threshold of minimum 0.1% and maximum 5% of waytotals. Rotating 3-dimensional view. S4 Figure B1: S. attenuata, left IC tracts shown in blue, right IC tracts shown in red, minimum threshold set to 1% and maximum threshold set to 30% of waytotals. Orthographic view. S4 Figure B2: S. attenuata, left IC tracts shown in blue, right IC tracts shown in red, set to a more liberal threshold of minimum 0.1% and maximum 5% of waytotals. Orthographic view. S4 Figure B3: S. attenuata, left IC tracts shown in blue, right IC tracts shown in red, set to a more liberal threshold of minimum 0.1% and maximum 5% of waytotals. Still [file pone.0323617.s001.zip › supporting_information/s5_fig_d2.png]

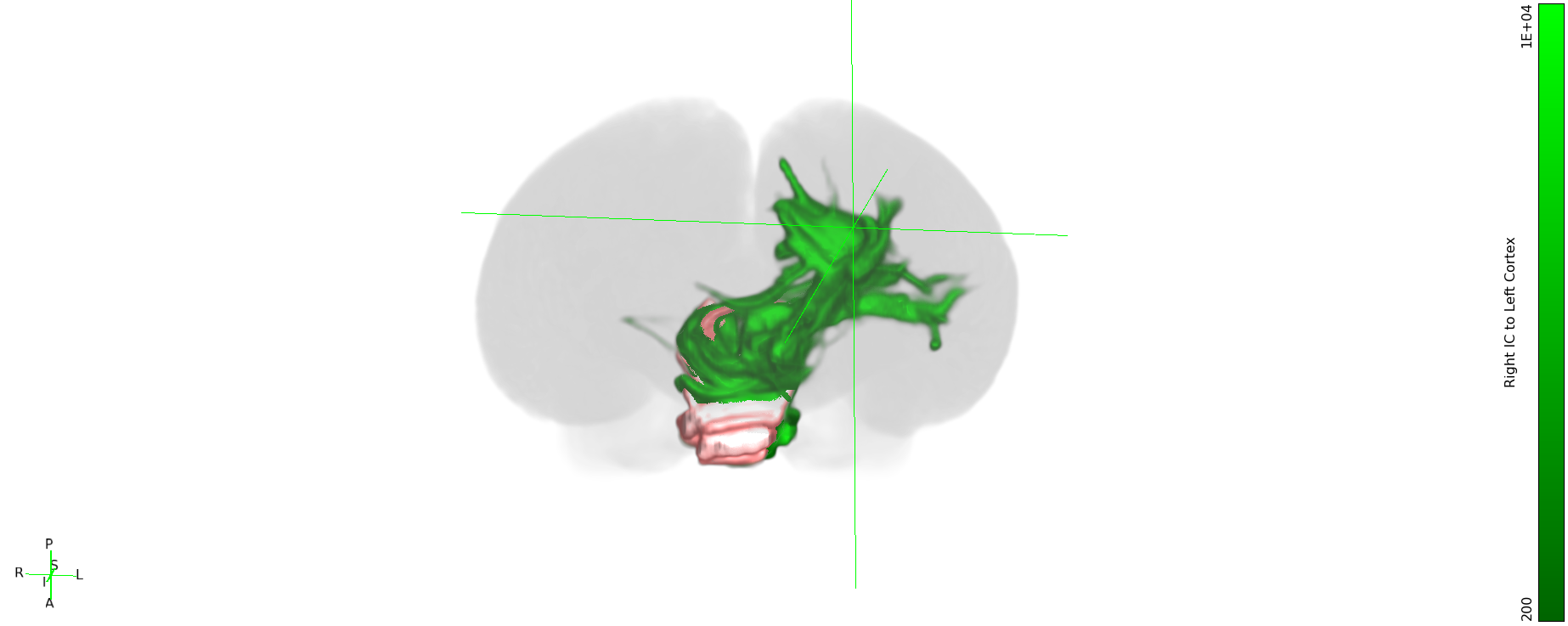

Supplement: S1 File — S1 Text. Detailed cerebellar and subcortical projection sites in IC-cerebellar traces. S2 Text. Detailed cortical projections in IC-cerebellar traces. S3 Figures. Masked regions of interest in FSLeyes. S3 Figure A. D. delphis. Red= right, blue=left for cerebella, yellow=right and turquoise=left for inferior colliculi. S3 Figure B. S. Attenuata. Red= right, blue=left for cerebella, yellow=right and turquoise=left for inferior colliculi. S3 Figure C. L. acutus. Red= right, blue=left for cerebella, yellow=right and turquoise=left for inferior colliculi. S3 Figure D. B. borealis. Red= right, blue=left for cerebella, yellow=right and turquoise=left for inferior colliculi. S4 Figures. Ascending auditory tractograms. S4 Figure A1: D. delphis, left IC tracts shown in blue, right IC tracts shown in red, minimum threshold set to 1% and maximum threshold set to 30% of waytotals. Orthographic view. S4 Figure A2: D. delphis, left IC tracts shown in blue, right IC tracts shown in red, set to a more liberal threshold of minimum 0.1% and maximum 5% of waytotals. Orthographic view. S4 Figure A3: D. delphis, left IC tracts shown in blue, right IC tracts shown in red, set to a more liberal threshold of minimum 0.1% and maximum 5% of waytotals. Still 3-dimensional view. S4 Figure A4: D. delphis, left IC tracts shown in blue, right IC tracts shown in red, set to a more liberal threshold of minimum 0.1% and maximum 5% of waytotals. Rotating 3-dimensional view. S4 Figure B1: S. attenuata, left IC tracts shown in blue, right IC tracts shown in red, minimum threshold set to 1% and maximum threshold set to 30% of waytotals. Orthographic view. S4 Figure B2: S. attenuata, left IC tracts shown in blue, right IC tracts shown in red, set to a more liberal threshold of minimum 0.1% and maximum 5% of waytotals. Orthographic view. S4 Figure B3: S. attenuata, left IC tracts shown in blue, right IC tracts shown in red, set to a more liberal threshold of minimum 0.1% and maximum 5% of waytotals. Still [file pone.0323617.s001.zip › supporting_information/s5_fig_a3.png]

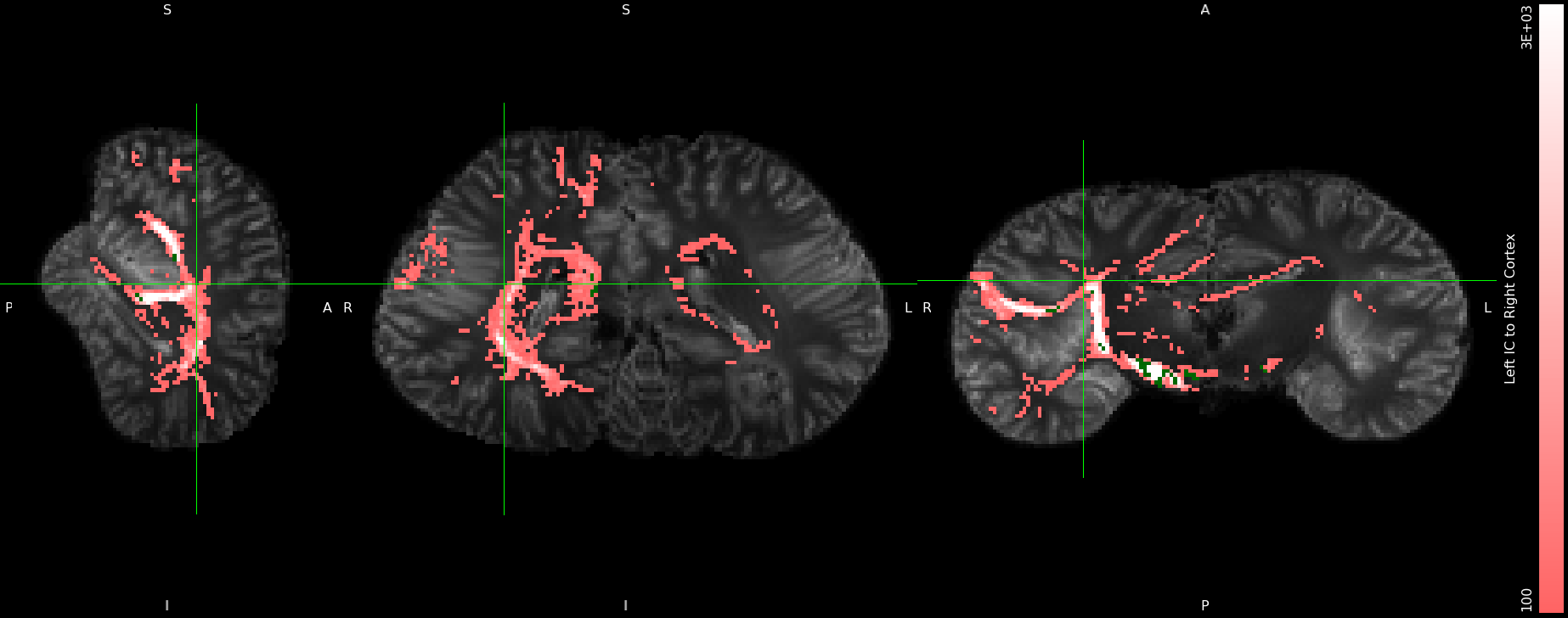

Supplement: S1 File — S1 Text. Detailed cerebellar and subcortical projection sites in IC-cerebellar traces. S2 Text. Detailed cortical projections in IC-cerebellar traces. S3 Figures. Masked regions of interest in FSLeyes. S3 Figure A. D. delphis. Red= right, blue=left for cerebella, yellow=right and turquoise=left for inferior colliculi. S3 Figure B. S. Attenuata. Red= right, blue=left for cerebella, yellow=right and turquoise=left for inferior colliculi. S3 Figure C. L. acutus. Red= right, blue=left for cerebella, yellow=right and turquoise=left for inferior colliculi. S3 Figure D. B. borealis. Red= right, blue=left for cerebella, yellow=right and turquoise=left for inferior colliculi. S4 Figures. Ascending auditory tractograms. S4 Figure A1: D. delphis, left IC tracts shown in blue, right IC tracts shown in red, minimum threshold set to 1% and maximum threshold set to 30% of waytotals. Orthographic view. S4 Figure A2: D. delphis, left IC tracts shown in blue, right IC tracts shown in red, set to a more liberal threshold of minimum 0.1% and maximum 5% of waytotals. Orthographic view. S4 Figure A3: D. delphis, left IC tracts shown in blue, right IC tracts shown in red, set to a more liberal threshold of minimum 0.1% and maximum 5% of waytotals. Still 3-dimensional view. S4 Figure A4: D. delphis, left IC tracts shown in blue, right IC tracts shown in red, set to a more liberal threshold of minimum 0.1% and maximum 5% of waytotals. Rotating 3-dimensional view. S4 Figure B1: S. attenuata, left IC tracts shown in blue, right IC tracts shown in red, minimum threshold set to 1% and maximum threshold set to 30% of waytotals. Orthographic view. S4 Figure B2: S. attenuata, left IC tracts shown in blue, right IC tracts shown in red, set to a more liberal threshold of minimum 0.1% and maximum 5% of waytotals. Orthographic view. S4 Figure B3: S. attenuata, left IC tracts shown in blue, right IC tracts shown in red, set to a more liberal threshold of minimum 0.1% and maximum 5% of waytotals. Still [file pone.0323617.s001.zip › supporting_information/s5_fig_c1.png]

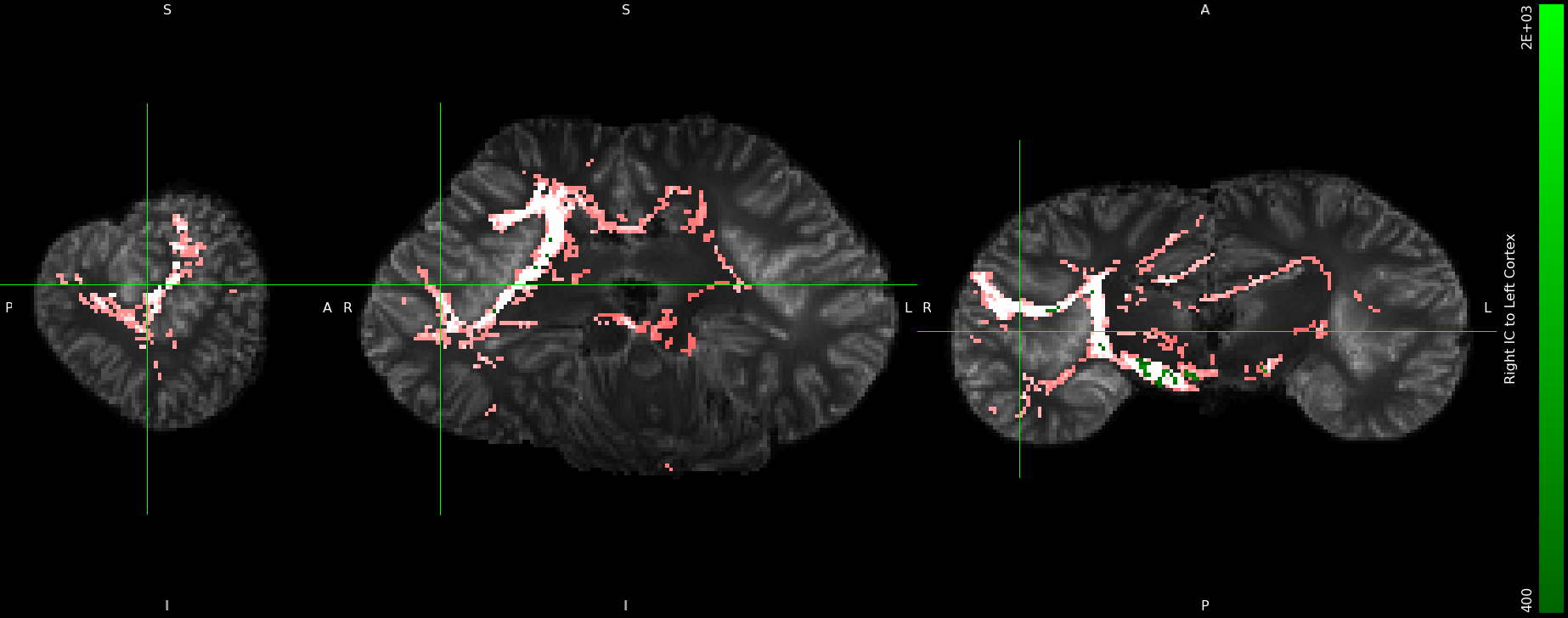

Supplement: S1 File — S1 Text. Detailed cerebellar and subcortical projection sites in IC-cerebellar traces. S2 Text. Detailed cortical projections in IC-cerebellar traces. S3 Figures. Masked regions of interest in FSLeyes. S3 Figure A. D. delphis. Red= right, blue=left for cerebella, yellow=right and turquoise=left for inferior colliculi. S3 Figure B. S. Attenuata. Red= right, blue=left for cerebella, yellow=right and turquoise=left for inferior colliculi. S3 Figure C. L. acutus. Red= right, blue=left for cerebella, yellow=right and turquoise=left for inferior colliculi. S3 Figure D. B. borealis. Red= right, blue=left for cerebella, yellow=right and turquoise=left for inferior colliculi. S4 Figures. Ascending auditory tractograms. S4 Figure A1: D. delphis, left IC tracts shown in blue, right IC tracts shown in red, minimum threshold set to 1% and maximum threshold set to 30% of waytotals. Orthographic view. S4 Figure A2: D. delphis, left IC tracts shown in blue, right IC tracts shown in red, set to a more liberal threshold of minimum 0.1% and maximum 5% of waytotals. Orthographic view. S4 Figure A3: D. delphis, left IC tracts shown in blue, right IC tracts shown in red, set to a more liberal threshold of minimum 0.1% and maximum 5% of waytotals. Still 3-dimensional view. S4 Figure A4: D. delphis, left IC tracts shown in blue, right IC tracts shown in red, set to a more liberal threshold of minimum 0.1% and maximum 5% of waytotals. Rotating 3-dimensional view. S4 Figure B1: S. attenuata, left IC tracts shown in blue, right IC tracts shown in red, minimum threshold set to 1% and maximum threshold set to 30% of waytotals. Orthographic view. S4 Figure B2: S. attenuata, left IC tracts shown in blue, right IC tracts shown in red, set to a more liberal threshold of minimum 0.1% and maximum 5% of waytotals. Orthographic view. S4 Figure B3: S. attenuata, left IC tracts shown in blue, right IC tracts shown in red, set to a more liberal threshold of minimum 0.1% and maximum 5% of waytotals. Still [file pone.0323617.s001.zip › supporting_information/s5_fig_c2.png]

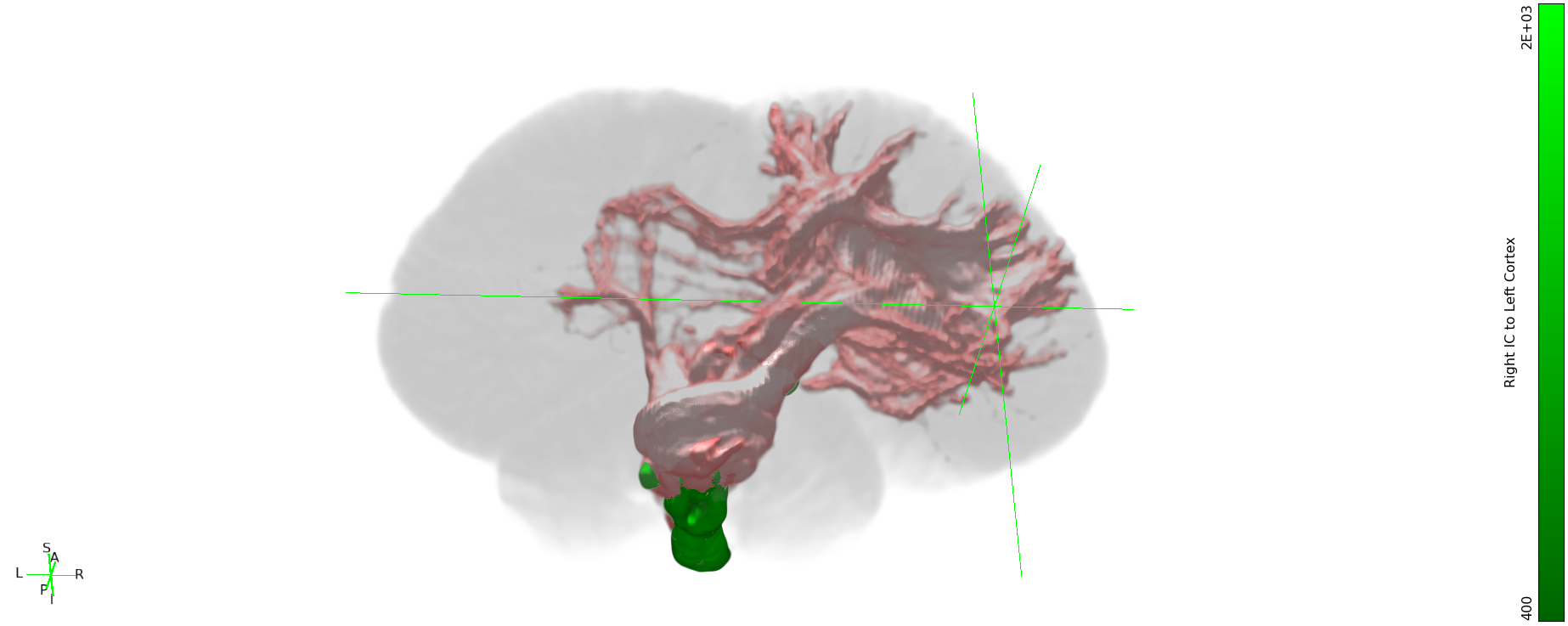

Supplement: S1 File — S1 Text. Detailed cerebellar and subcortical projection sites in IC-cerebellar traces. S2 Text. Detailed cortical projections in IC-cerebellar traces. S3 Figures. Masked regions of interest in FSLeyes. S3 Figure A. D. delphis. Red= right, blue=left for cerebella, yellow=right and turquoise=left for inferior colliculi. S3 Figure B. S. Attenuata. Red= right, blue=left for cerebella, yellow=right and turquoise=left for inferior colliculi. S3 Figure C. L. acutus. Red= right, blue=left for cerebella, yellow=right and turquoise=left for inferior colliculi. S3 Figure D. B. borealis. Red= right, blue=left for cerebella, yellow=right and turquoise=left for inferior colliculi. S4 Figures. Ascending auditory tractograms. S4 Figure A1: D. delphis, left IC tracts shown in blue, right IC tracts shown in red, minimum threshold set to 1% and maximum threshold set to 30% of waytotals. Orthographic view. S4 Figure A2: D. delphis, left IC tracts shown in blue, right IC tracts shown in red, set to a more liberal threshold of minimum 0.1% and maximum 5% of waytotals. Orthographic view. S4 Figure A3: D. delphis, left IC tracts shown in blue, right IC tracts shown in red, set to a more liberal threshold of minimum 0.1% and maximum 5% of waytotals. Still 3-dimensional view. S4 Figure A4: D. delphis, left IC tracts shown in blue, right IC tracts shown in red, set to a more liberal threshold of minimum 0.1% and maximum 5% of waytotals. Rotating 3-dimensional view. S4 Figure B1: S. attenuata, left IC tracts shown in blue, right IC tracts shown in red, minimum threshold set to 1% and maximum threshold set to 30% of waytotals. Orthographic view. S4 Figure B2: S. attenuata, left IC tracts shown in blue, right IC tracts shown in red, set to a more liberal threshold of minimum 0.1% and maximum 5% of waytotals. Orthographic view. S4 Figure B3: S. attenuata, left IC tracts shown in blue, right IC tracts shown in red, set to a more liberal threshold of minimum 0.1% and maximum 5% of waytotals. Still [file pone.0323617.s001.zip › supporting_information/s5_fig_c3.png]

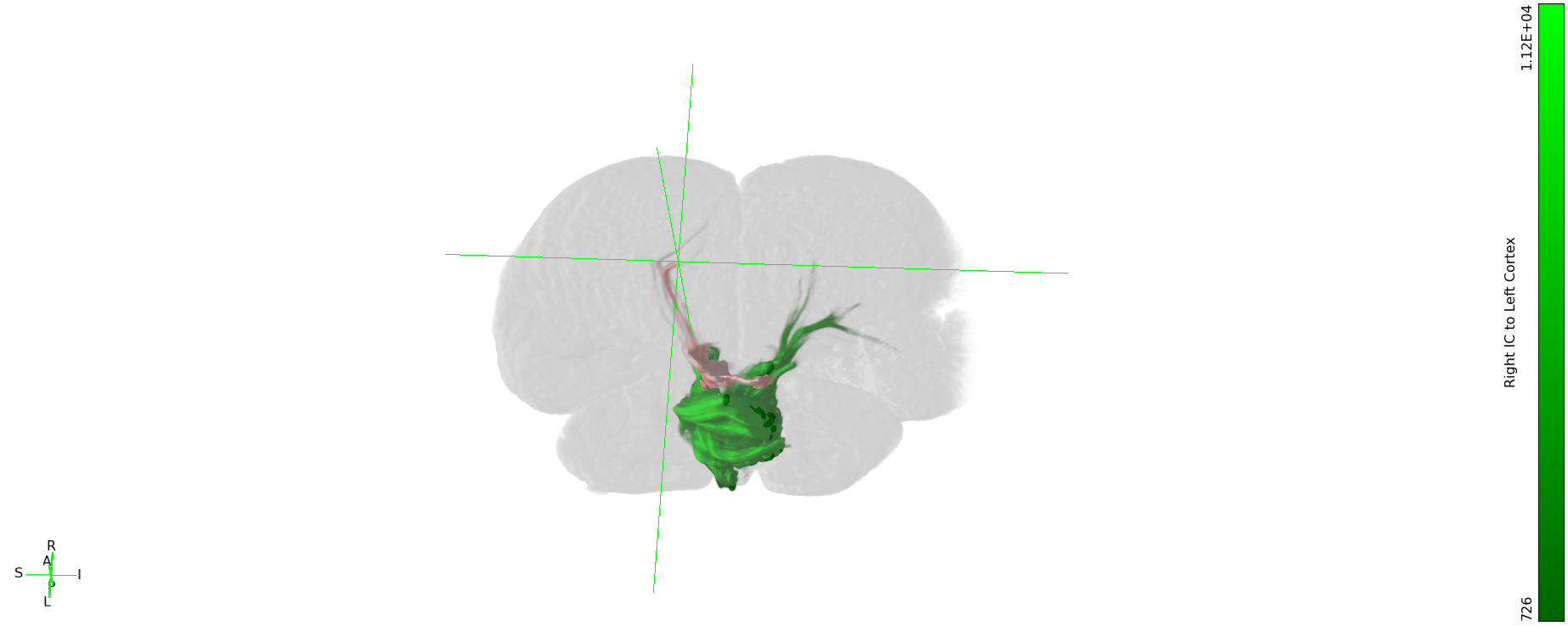

Supplement: S1 File — S1 Text. Detailed cerebellar and subcortical projection sites in IC-cerebellar traces. S2 Text. Detailed cortical projections in IC-cerebellar traces. S3 Figures. Masked regions of interest in FSLeyes. S3 Figure A. D. delphis. Red= right, blue=left for cerebella, yellow=right and turquoise=left for inferior colliculi. S3 Figure B. S. Attenuata. Red= right, blue=left for cerebella, yellow=right and turquoise=left for inferior colliculi. S3 Figure C. L. acutus. Red= right, blue=left for cerebella, yellow=right and turquoise=left for inferior colliculi. S3 Figure D. B. borealis. Red= right, blue=left for cerebella, yellow=right and turquoise=left for inferior colliculi. S4 Figures. Ascending auditory tractograms. S4 Figure A1: D. delphis, left IC tracts shown in blue, right IC tracts shown in red, minimum threshold set to 1% and maximum threshold set to 30% of waytotals. Orthographic view. S4 Figure A2: D. delphis, left IC tracts shown in blue, right IC tracts shown in red, set to a more liberal threshold of minimum 0.1% and maximum 5% of waytotals. Orthographic view. S4 Figure A3: D. delphis, left IC tracts shown in blue, right IC tracts shown in red, set to a more liberal threshold of minimum 0.1% and maximum 5% of waytotals. Still 3-dimensional view. S4 Figure A4: D. delphis, left IC tracts shown in blue, right IC tracts shown in red, set to a more liberal threshold of minimum 0.1% and maximum 5% of waytotals. Rotating 3-dimensional view. S4 Figure B1: S. attenuata, left IC tracts shown in blue, right IC tracts shown in red, minimum threshold set to 1% and maximum threshold set to 30% of waytotals. Orthographic view. S4 Figure B2: S. attenuata, left IC tracts shown in blue, right IC tracts shown in red, set to a more liberal threshold of minimum 0.1% and maximum 5% of waytotals. Orthographic view. S4 Figure B3: S. attenuata, left IC tracts shown in blue, right IC tracts shown in red, set to a more liberal threshold of minimum 0.1% and maximum 5% of waytotals. Still [file pone.0323617.s001.zip › supporting_information/s5_fig_d3.png]

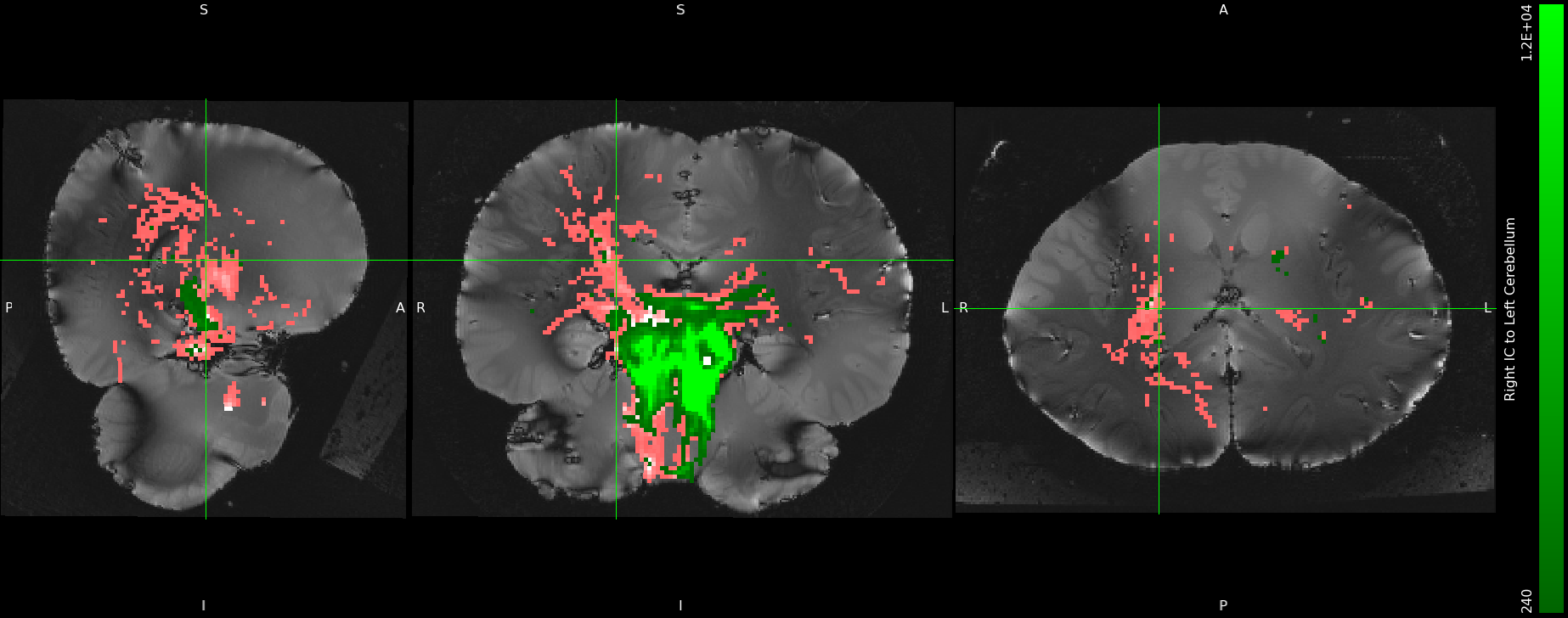

Supplement: S1 File — S1 Text. Detailed cerebellar and subcortical projection sites in IC-cerebellar traces. S2 Text. Detailed cortical projections in IC-cerebellar traces. S3 Figures. Masked regions of interest in FSLeyes. S3 Figure A. D. delphis. Red= right, blue=left for cerebella, yellow=right and turquoise=left for inferior colliculi. S3 Figure B. S. Attenuata. Red= right, blue=left for cerebella, yellow=right and turquoise=left for inferior colliculi. S3 Figure C. L. acutus. Red= right, blue=left for cerebella, yellow=right and turquoise=left for inferior colliculi. S3 Figure D. B. borealis. Red= right, blue=left for cerebella, yellow=right and turquoise=left for inferior colliculi. S4 Figures. Ascending auditory tractograms. S4 Figure A1: D. delphis, left IC tracts shown in blue, right IC tracts shown in red, minimum threshold set to 1% and maximum threshold set to 30% of waytotals. Orthographic view. S4 Figure A2: D. delphis, left IC tracts shown in blue, right IC tracts shown in red, set to a more liberal threshold of minimum 0.1% and maximum 5% of waytotals. Orthographic view. S4 Figure A3: D. delphis, left IC tracts shown in blue, right IC tracts shown in red, set to a more liberal threshold of minimum 0.1% and maximum 5% of waytotals. Still 3-dimensional view. S4 Figure A4: D. delphis, left IC tracts shown in blue, right IC tracts shown in red, set to a more liberal threshold of minimum 0.1% and maximum 5% of waytotals. Rotating 3-dimensional view. S4 Figure B1: S. attenuata, left IC tracts shown in blue, right IC tracts shown in red, minimum threshold set to 1% and maximum threshold set to 30% of waytotals. Orthographic view. S4 Figure B2: S. attenuata, left IC tracts shown in blue, right IC tracts shown in red, set to a more liberal threshold of minimum 0.1% and maximum 5% of waytotals. Orthographic view. S4 Figure B3: S. attenuata, left IC tracts shown in blue, right IC tracts shown in red, set to a more liberal threshold of minimum 0.1% and maximum 5% of waytotals. Still [file pone.0323617.s001.zip › supporting_information/s5_fig_b2.png]

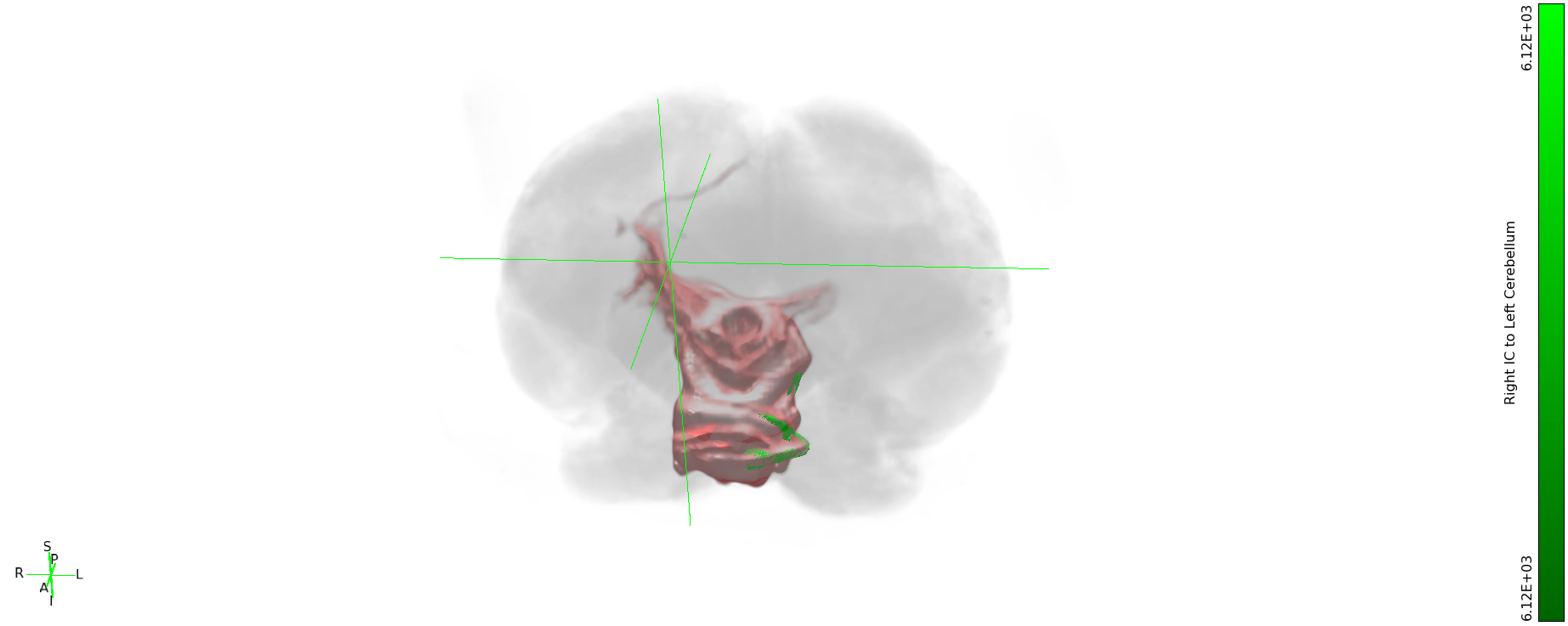

Supplement: S1 File — S1 Text. Detailed cerebellar and subcortical projection sites in IC-cerebellar traces. S2 Text. Detailed cortical projections in IC-cerebellar traces. S3 Figures. Masked regions of interest in FSLeyes. S3 Figure A. D. delphis. Red= right, blue=left for cerebella, yellow=right and turquoise=left for inferior colliculi. S3 Figure B. S. Attenuata. Red= right, blue=left for cerebella, yellow=right and turquoise=left for inferior colliculi. S3 Figure C. L. acutus. Red= right, blue=left for cerebella, yellow=right and turquoise=left for inferior colliculi. S3 Figure D. B. borealis. Red= right, blue=left for cerebella, yellow=right and turquoise=left for inferior colliculi. S4 Figures. Ascending auditory tractograms. S4 Figure A1: D. delphis, left IC tracts shown in blue, right IC tracts shown in red, minimum threshold set to 1% and maximum threshold set to 30% of waytotals. Orthographic view. S4 Figure A2: D. delphis, left IC tracts shown in blue, right IC tracts shown in red, set to a more liberal threshold of minimum 0.1% and maximum 5% of waytotals. Orthographic view. S4 Figure A3: D. delphis, left IC tracts shown in blue, right IC tracts shown in red, set to a more liberal threshold of minimum 0.1% and maximum 5% of waytotals. Still 3-dimensional view. S4 Figure A4: D. delphis, left IC tracts shown in blue, right IC tracts shown in red, set to a more liberal threshold of minimum 0.1% and maximum 5% of waytotals. Rotating 3-dimensional view. S4 Figure B1: S. attenuata, left IC tracts shown in blue, right IC tracts shown in red, minimum threshold set to 1% and maximum threshold set to 30% of waytotals. Orthographic view. S4 Figure B2: S. attenuata, left IC tracts shown in blue, right IC tracts shown in red, set to a more liberal threshold of minimum 0.1% and maximum 5% of waytotals. Orthographic view. S4 Figure B3: S. attenuata, left IC tracts shown in blue, right IC tracts shown in red, set to a more liberal threshold of minimum 0.1% and maximum 5% of waytotals. Still [file pone.0323617.s001.zip › supporting_information/s5_fig_b3.png]

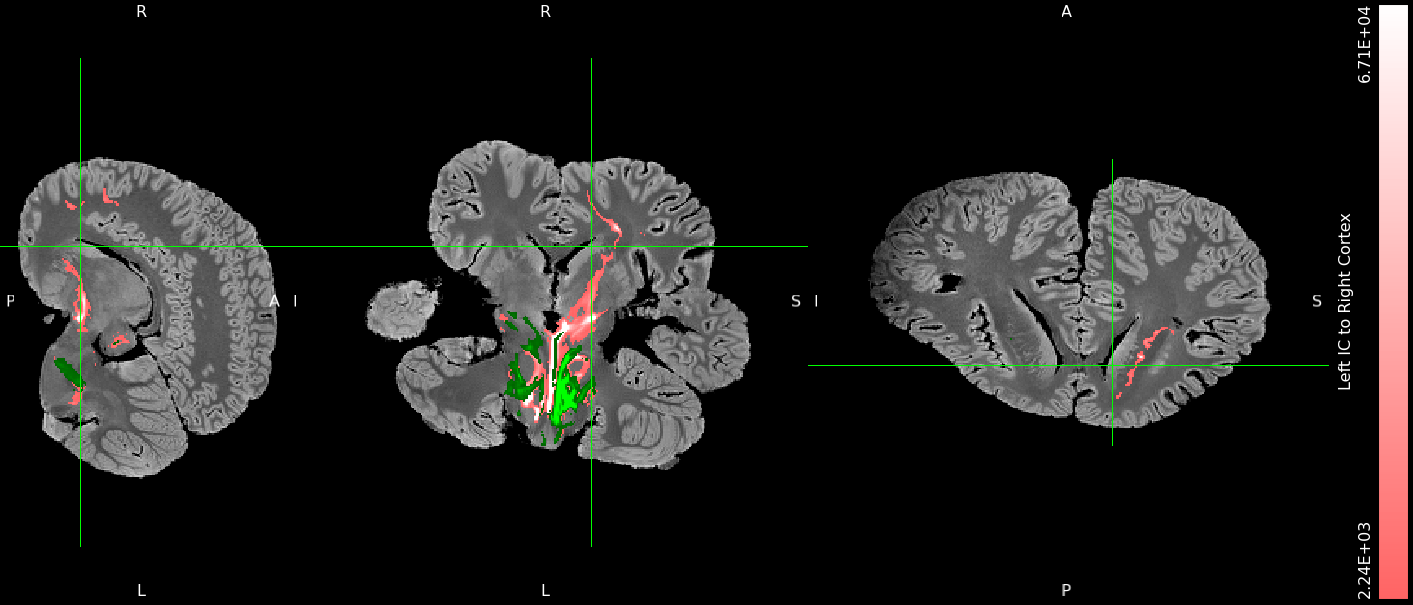

Supplement: S1 File — S1 Text. Detailed cerebellar and subcortical projection sites in IC-cerebellar traces. S2 Text. Detailed cortical projections in IC-cerebellar traces. S3 Figures. Masked regions of interest in FSLeyes. S3 Figure A. D. delphis. Red= right, blue=left for cerebella, yellow=right and turquoise=left for inferior colliculi. S3 Figure B. S. Attenuata. Red= right, blue=left for cerebella, yellow=right and turquoise=left for inferior colliculi. S3 Figure C. L. acutus. Red= right, blue=left for cerebella, yellow=right and turquoise=left for inferior colliculi. S3 Figure D. B. borealis. Red= right, blue=left for cerebella, yellow=right and turquoise=left for inferior colliculi. S4 Figures. Ascending auditory tractograms. S4 Figure A1: D. delphis, left IC tracts shown in blue, right IC tracts shown in red, minimum threshold set to 1% and maximum threshold set to 30% of waytotals. Orthographic view. S4 Figure A2: D. delphis, left IC tracts shown in blue, right IC tracts shown in red, set to a more liberal threshold of minimum 0.1% and maximum 5% of waytotals. Orthographic view. S4 Figure A3: D. delphis, left IC tracts shown in blue, right IC tracts shown in red, set to a more liberal threshold of minimum 0.1% and maximum 5% of waytotals. Still 3-dimensional view. S4 Figure A4: D. delphis, left IC tracts shown in blue, right IC tracts shown in red, set to a more liberal threshold of minimum 0.1% and maximum 5% of waytotals. Rotating 3-dimensional view. S4 Figure B1: S. attenuata, left IC tracts shown in blue, right IC tracts shown in red, minimum threshold set to 1% and maximum threshold set to 30% of waytotals. Orthographic view. S4 Figure B2: S. attenuata, left IC tracts shown in blue, right IC tracts shown in red, set to a more liberal threshold of minimum 0.1% and maximum 5% of waytotals. Orthographic view. S4 Figure B3: S. attenuata, left IC tracts shown in blue, right IC tracts shown in red, set to a more liberal threshold of minimum 0.1% and maximum 5% of waytotals. Still [file pone.0323617.s001.zip › supporting_information/s5_fig_d1.png]

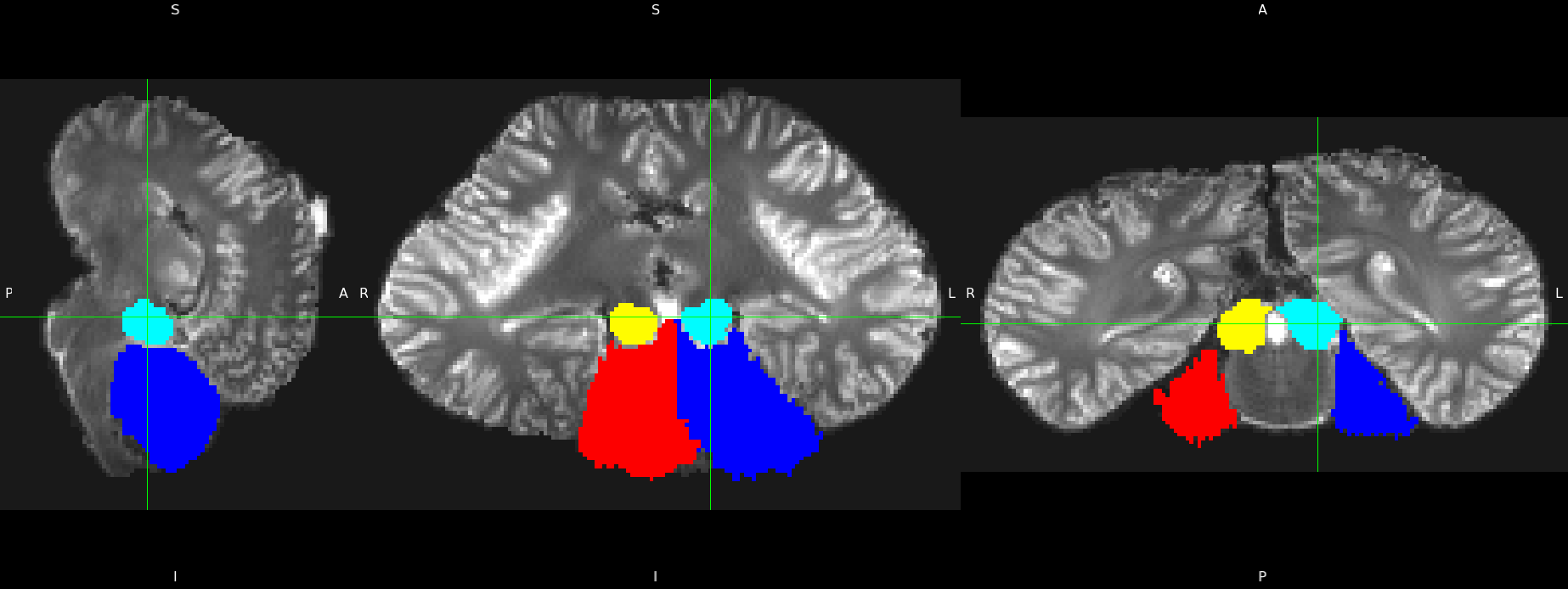

Supplement: S1 File — S1 Text. Detailed cerebellar and subcortical projection sites in IC-cerebellar traces. S2 Text. Detailed cortical projections in IC-cerebellar traces. S3 Figures. Masked regions of interest in FSLeyes. S3 Figure A. D. delphis. Red= right, blue=left for cerebella, yellow=right and turquoise=left for inferior colliculi. S3 Figure B. S. Attenuata. Red= right, blue=left for cerebella, yellow=right and turquoise=left for inferior colliculi. S3 Figure C. L. acutus. Red= right, blue=left for cerebella, yellow=right and turquoise=left for inferior colliculi. S3 Figure D. B. borealis. Red= right, blue=left for cerebella, yellow=right and turquoise=left for inferior colliculi. S4 Figures. Ascending auditory tractograms. S4 Figure A1: D. delphis, left IC tracts shown in blue, right IC tracts shown in red, minimum threshold set to 1% and maximum threshold set to 30% of waytotals. Orthographic view. S4 Figure A2: D. delphis, left IC tracts shown in blue, right IC tracts shown in red, set to a more liberal threshold of minimum 0.1% and maximum 5% of waytotals. Orthographic view. S4 Figure A3: D. delphis, left IC tracts shown in blue, right IC tracts shown in red, set to a more liberal threshold of minimum 0.1% and maximum 5% of waytotals. Still 3-dimensional view. S4 Figure A4: D. delphis, left IC tracts shown in blue, right IC tracts shown in red, set to a more liberal threshold of minimum 0.1% and maximum 5% of waytotals. Rotating 3-dimensional view. S4 Figure B1: S. attenuata, left IC tracts shown in blue, right IC tracts shown in red, minimum threshold set to 1% and maximum threshold set to 30% of waytotals. Orthographic view. S4 Figure B2: S. attenuata, left IC tracts shown in blue, right IC tracts shown in red, set to a more liberal threshold of minimum 0.1% and maximum 5% of waytotals. Orthographic view. S4 Figure B3: S. attenuata, left IC tracts shown in blue, right IC tracts shown in red, set to a more liberal threshold of minimum 0.1% and maximum 5% of waytotals. Still [file pone.0323617.s001.zip › supporting_information/s3_fig_c.png]

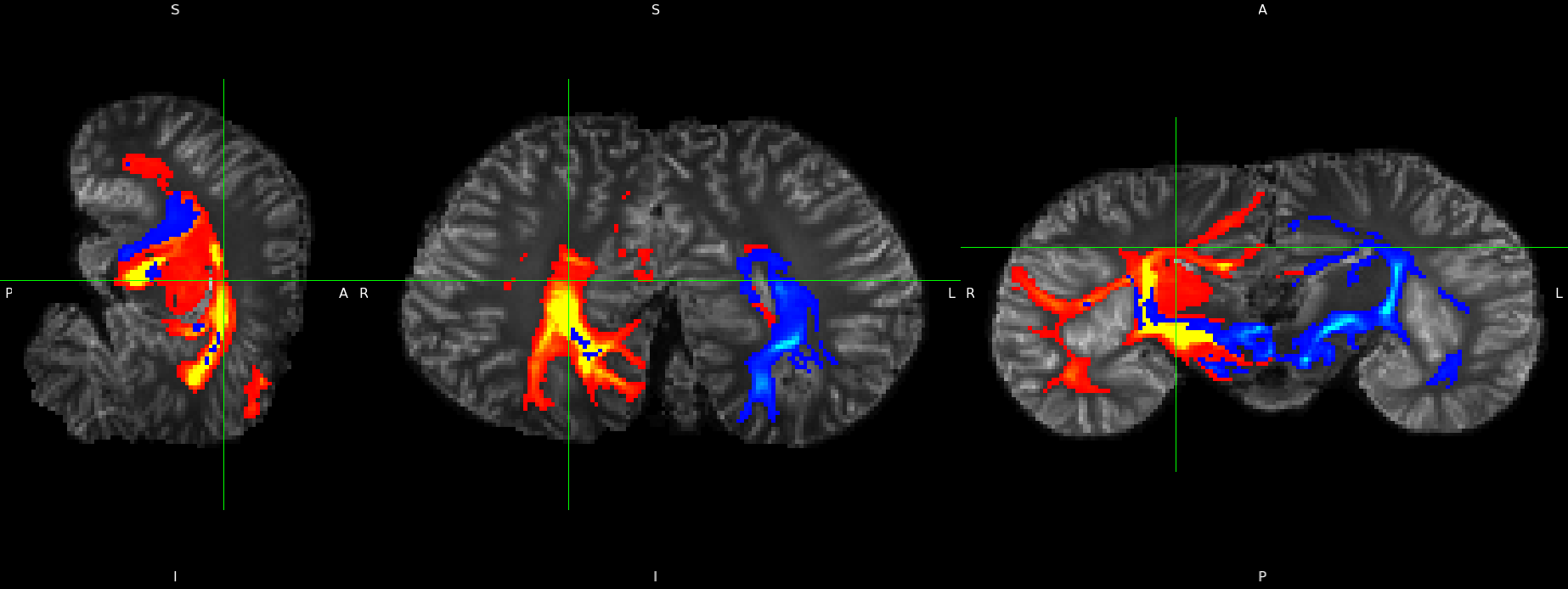

Supplement: S1 File — S1 Text. Detailed cerebellar and subcortical projection sites in IC-cerebellar traces. S2 Text. Detailed cortical projections in IC-cerebellar traces. S3 Figures. Masked regions of interest in FSLeyes. S3 Figure A. D. delphis. Red= right, blue=left for cerebella, yellow=right and turquoise=left for inferior colliculi. S3 Figure B. S. Attenuata. Red= right, blue=left for cerebella, yellow=right and turquoise=left for inferior colliculi. S3 Figure C. L. acutus. Red= right, blue=left for cerebella, yellow=right and turquoise=left for inferior colliculi. S3 Figure D. B. borealis. Red= right, blue=left for cerebella, yellow=right and turquoise=left for inferior colliculi. S4 Figures. Ascending auditory tractograms. S4 Figure A1: D. delphis, left IC tracts shown in blue, right IC tracts shown in red, minimum threshold set to 1% and maximum threshold set to 30% of waytotals. Orthographic view. S4 Figure A2: D. delphis, left IC tracts shown in blue, right IC tracts shown in red, set to a more liberal threshold of minimum 0.1% and maximum 5% of waytotals. Orthographic view. S4 Figure A3: D. delphis, left IC tracts shown in blue, right IC tracts shown in red, set to a more liberal threshold of minimum 0.1% and maximum 5% of waytotals. Still 3-dimensional view. S4 Figure A4: D. delphis, left IC tracts shown in blue, right IC tracts shown in red, set to a more liberal threshold of minimum 0.1% and maximum 5% of waytotals. Rotating 3-dimensional view. S4 Figure B1: S. attenuata, left IC tracts shown in blue, right IC tracts shown in red, minimum threshold set to 1% and maximum threshold set to 30% of waytotals. Orthographic view. S4 Figure B2: S. attenuata, left IC tracts shown in blue, right IC tracts shown in red, set to a more liberal threshold of minimum 0.1% and maximum 5% of waytotals. Orthographic view. S4 Figure B3: S. attenuata, left IC tracts shown in blue, right IC tracts shown in red, set to a more liberal threshold of minimum 0.1% and maximum 5% of waytotals. Still [file pone.0323617.s001.zip › supporting_information/s4_fig_c2.png]

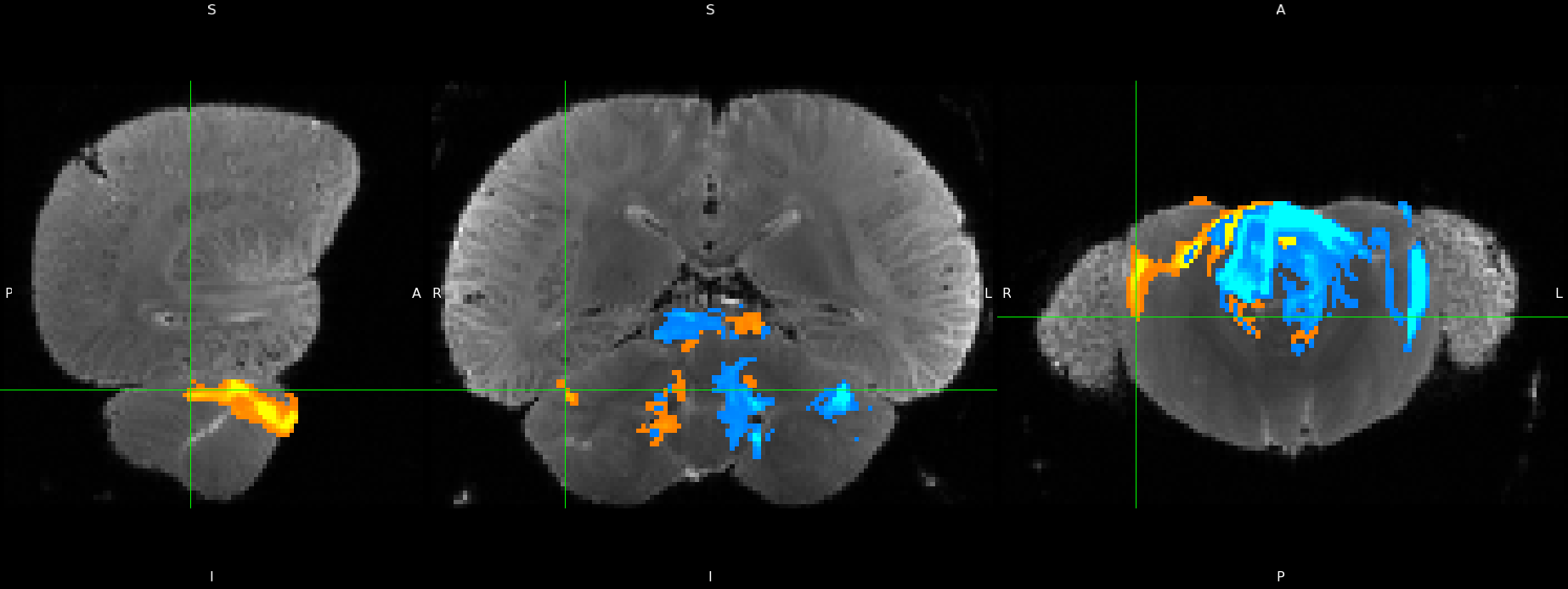

Supplement: S1 File — S1 Text. Detailed cerebellar and subcortical projection sites in IC-cerebellar traces. S2 Text. Detailed cortical projections in IC-cerebellar traces. S3 Figures. Masked regions of interest in FSLeyes. S3 Figure A. D. delphis. Red= right, blue=left for cerebella, yellow=right and turquoise=left for inferior colliculi. S3 Figure B. S. Attenuata. Red= right, blue=left for cerebella, yellow=right and turquoise=left for inferior colliculi. S3 Figure C. L. acutus. Red= right, blue=left for cerebella, yellow=right and turquoise=left for inferior colliculi. S3 Figure D. B. borealis. Red= right, blue=left for cerebella, yellow=right and turquoise=left for inferior colliculi. S4 Figures. Ascending auditory tractograms. S4 Figure A1: D. delphis, left IC tracts shown in blue, right IC tracts shown in red, minimum threshold set to 1% and maximum threshold set to 30% of waytotals. Orthographic view. S4 Figure A2: D. delphis, left IC tracts shown in blue, right IC tracts shown in red, set to a more liberal threshold of minimum 0.1% and maximum 5% of waytotals. Orthographic view. S4 Figure A3: D. delphis, left IC tracts shown in blue, right IC tracts shown in red, set to a more liberal threshold of minimum 0.1% and maximum 5% of waytotals. Still 3-dimensional view. S4 Figure A4: D. delphis, left IC tracts shown in blue, right IC tracts shown in red, set to a more liberal threshold of minimum 0.1% and maximum 5% of waytotals. Rotating 3-dimensional view. S4 Figure B1: S. attenuata, left IC tracts shown in blue, right IC tracts shown in red, minimum threshold set to 1% and maximum threshold set to 30% of waytotals. Orthographic view. S4 Figure B2: S. attenuata, left IC tracts shown in blue, right IC tracts shown in red, set to a more liberal threshold of minimum 0.1% and maximum 5% of waytotals. Orthographic view. S4 Figure B3: S. attenuata, left IC tracts shown in blue, right IC tracts shown in red, set to a more liberal threshold of minimum 0.1% and maximum 5% of waytotals. Still [file pone.0323617.s001.zip › supporting_information/s7_fig_a1.png]

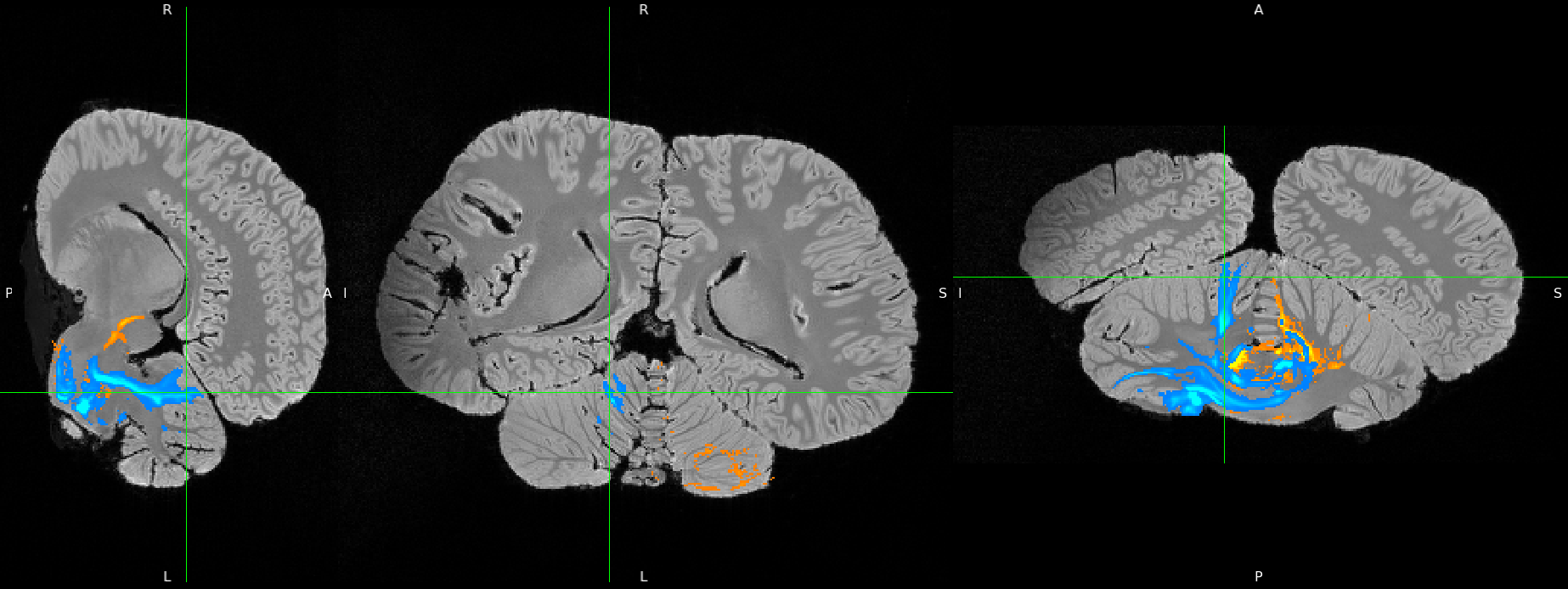

Supplement: S1 File — S1 Text. Detailed cerebellar and subcortical projection sites in IC-cerebellar traces. S2 Text. Detailed cortical projections in IC-cerebellar traces. S3 Figures. Masked regions of interest in FSLeyes. S3 Figure A. D. delphis. Red= right, blue=left for cerebella, yellow=right and turquoise=left for inferior colliculi. S3 Figure B. S. Attenuata. Red= right, blue=left for cerebella, yellow=right and turquoise=left for inferior colliculi. S3 Figure C. L. acutus. Red= right, blue=left for cerebella, yellow=right and turquoise=left for inferior colliculi. S3 Figure D. B. borealis. Red= right, blue=left for cerebella, yellow=right and turquoise=left for inferior colliculi. S4 Figures. Ascending auditory tractograms. S4 Figure A1: D. delphis, left IC tracts shown in blue, right IC tracts shown in red, minimum threshold set to 1% and maximum threshold set to 30% of waytotals. Orthographic view. S4 Figure A2: D. delphis, left IC tracts shown in blue, right IC tracts shown in red, set to a more liberal threshold of minimum 0.1% and maximum 5% of waytotals. Orthographic view. S4 Figure A3: D. delphis, left IC tracts shown in blue, right IC tracts shown in red, set to a more liberal threshold of minimum 0.1% and maximum 5% of waytotals. Still 3-dimensional view. S4 Figure A4: D. delphis, left IC tracts shown in blue, right IC tracts shown in red, set to a more liberal threshold of minimum 0.1% and maximum 5% of waytotals. Rotating 3-dimensional view. S4 Figure B1: S. attenuata, left IC tracts shown in blue, right IC tracts shown in red, minimum threshold set to 1% and maximum threshold set to 30% of waytotals. Orthographic view. S4 Figure B2: S. attenuata, left IC tracts shown in blue, right IC tracts shown in red, set to a more liberal threshold of minimum 0.1% and maximum 5% of waytotals. Orthographic view. S4 Figure B3: S. attenuata, left IC tracts shown in blue, right IC tracts shown in red, set to a more liberal threshold of minimum 0.1% and maximum 5% of waytotals. Still [file pone.0323617.s001.zip › supporting_information/s7_fig_d2.png]

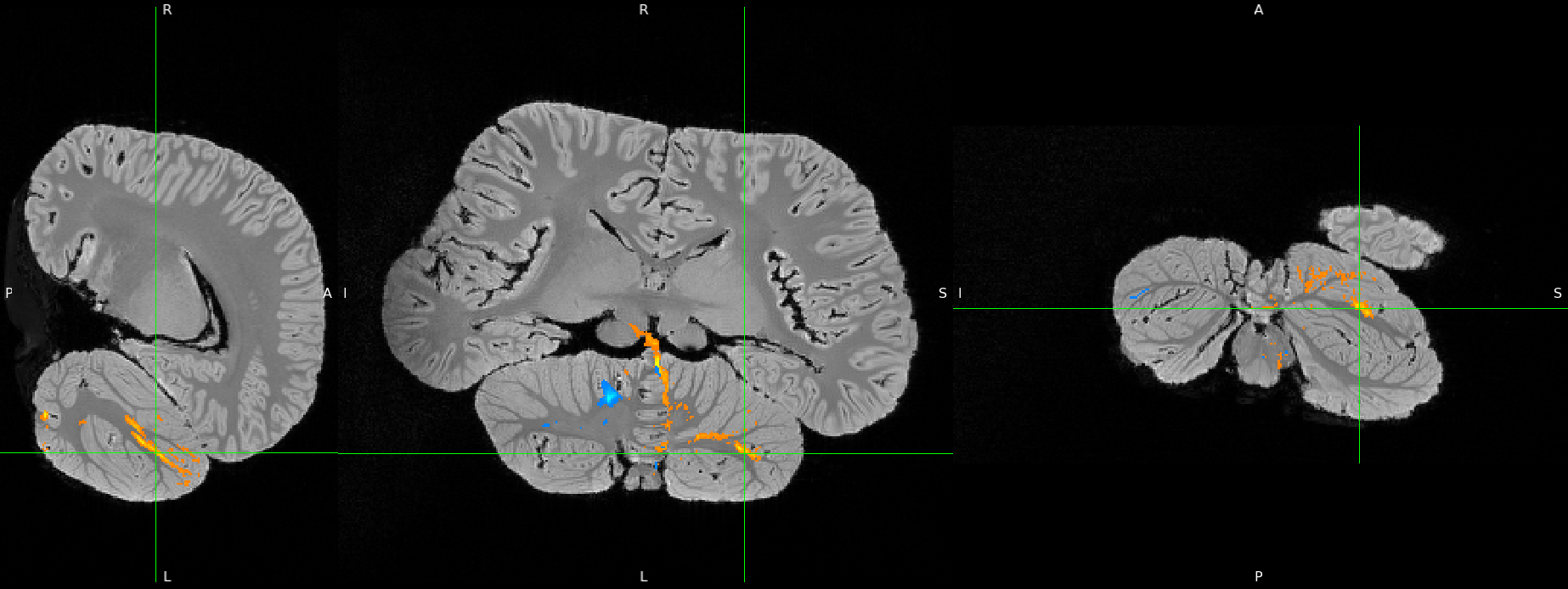

Supplement: S1 File — S1 Text. Detailed cerebellar and subcortical projection sites in IC-cerebellar traces. S2 Text. Detailed cortical projections in IC-cerebellar traces. S3 Figures. Masked regions of interest in FSLeyes. S3 Figure A. D. delphis. Red= right, blue=left for cerebella, yellow=right and turquoise=left for inferior colliculi. S3 Figure B. S. Attenuata. Red= right, blue=left for cerebella, yellow=right and turquoise=left for inferior colliculi. S3 Figure C. L. acutus. Red= right, blue=left for cerebella, yellow=right and turquoise=left for inferior colliculi. S3 Figure D. B. borealis. Red= right, blue=left for cerebella, yellow=right and turquoise=left for inferior colliculi. S4 Figures. Ascending auditory tractograms. S4 Figure A1: D. delphis, left IC tracts shown in blue, right IC tracts shown in red, minimum threshold set to 1% and maximum threshold set to 30% of waytotals. Orthographic view. S4 Figure A2: D. delphis, left IC tracts shown in blue, right IC tracts shown in red, set to a more liberal threshold of minimum 0.1% and maximum 5% of waytotals. Orthographic view. S4 Figure A3: D. delphis, left IC tracts shown in blue, right IC tracts shown in red, set to a more liberal threshold of minimum 0.1% and maximum 5% of waytotals. Still 3-dimensional view. S4 Figure A4: D. delphis, left IC tracts shown in blue, right IC tracts shown in red, set to a more liberal threshold of minimum 0.1% and maximum 5% of waytotals. Rotating 3-dimensional view. S4 Figure B1: S. attenuata, left IC tracts shown in blue, right IC tracts shown in red, minimum threshold set to 1% and maximum threshold set to 30% of waytotals. Orthographic view. S4 Figure B2: S. attenuata, left IC tracts shown in blue, right IC tracts shown in red, set to a more liberal threshold of minimum 0.1% and maximum 5% of waytotals. Orthographic view. S4 Figure B3: S. attenuata, left IC tracts shown in blue, right IC tracts shown in red, set to a more liberal threshold of minimum 0.1% and maximum 5% of waytotals. Still [file pone.0323617.s001.zip › supporting_information/s7_fig_d1.png]

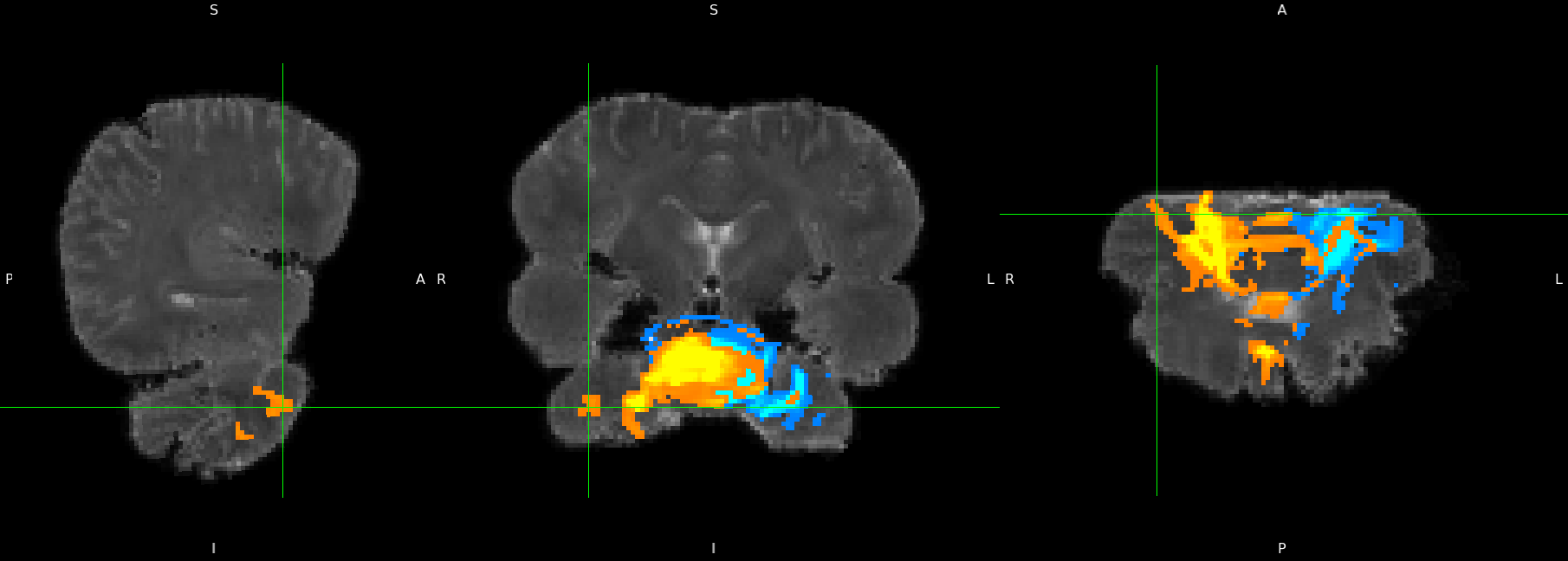

Supplement: S1 File — S1 Text. Detailed cerebellar and subcortical projection sites in IC-cerebellar traces. S2 Text. Detailed cortical projections in IC-cerebellar traces. S3 Figures. Masked regions of interest in FSLeyes. S3 Figure A. D. delphis. Red= right, blue=left for cerebella, yellow=right and turquoise=left for inferior colliculi. S3 Figure B. S. Attenuata. Red= right, blue=left for cerebella, yellow=right and turquoise=left for inferior colliculi. S3 Figure C. L. acutus. Red= right, blue=left for cerebella, yellow=right and turquoise=left for inferior colliculi. S3 Figure D. B. borealis. Red= right, blue=left for cerebella, yellow=right and turquoise=left for inferior colliculi. S4 Figures. Ascending auditory tractograms. S4 Figure A1: D. delphis, left IC tracts shown in blue, right IC tracts shown in red, minimum threshold set to 1% and maximum threshold set to 30% of waytotals. Orthographic view. S4 Figure A2: D. delphis, left IC tracts shown in blue, right IC tracts shown in red, set to a more liberal threshold of minimum 0.1% and maximum 5% of waytotals. Orthographic view. S4 Figure A3: D. delphis, left IC tracts shown in blue, right IC tracts shown in red, set to a more liberal threshold of minimum 0.1% and maximum 5% of waytotals. Still 3-dimensional view. S4 Figure A4: D. delphis, left IC tracts shown in blue, right IC tracts shown in red, set to a more liberal threshold of minimum 0.1% and maximum 5% of waytotals. Rotating 3-dimensional view. S4 Figure B1: S. attenuata, left IC tracts shown in blue, right IC tracts shown in red, minimum threshold set to 1% and maximum threshold set to 30% of waytotals. Orthographic view. S4 Figure B2: S. attenuata, left IC tracts shown in blue, right IC tracts shown in red, set to a more liberal threshold of minimum 0.1% and maximum 5% of waytotals. Orthographic view. S4 Figure B3: S. attenuata, left IC tracts shown in blue, right IC tracts shown in red, set to a more liberal threshold of minimum 0.1% and maximum 5% of waytotals. Still [file pone.0323617.s001.zip › supporting_information/s7_fig_b1.png]

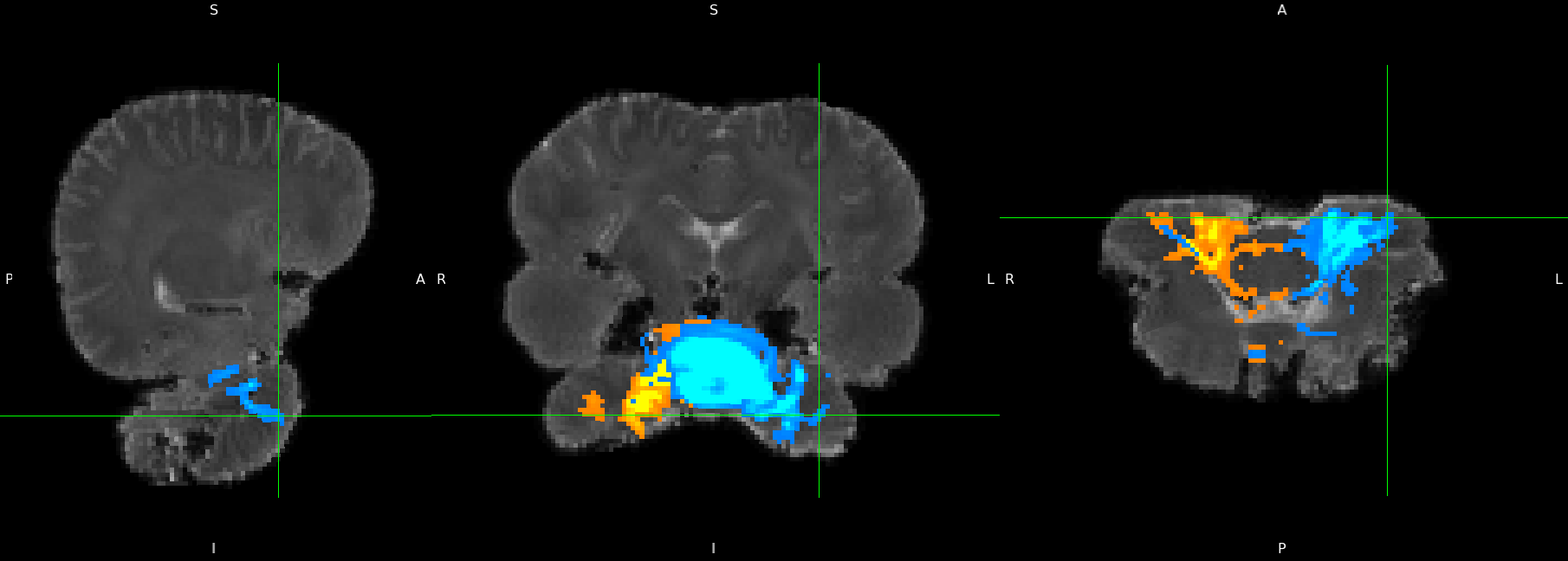

Supplement: S1 File — S1 Text. Detailed cerebellar and subcortical projection sites in IC-cerebellar traces. S2 Text. Detailed cortical projections in IC-cerebellar traces. S3 Figures. Masked regions of interest in FSLeyes. S3 Figure A. D. delphis. Red= right, blue=left for cerebella, yellow=right and turquoise=left for inferior colliculi. S3 Figure B. S. Attenuata. Red= right, blue=left for cerebella, yellow=right and turquoise=left for inferior colliculi. S3 Figure C. L. acutus. Red= right, blue=left for cerebella, yellow=right and turquoise=left for inferior colliculi. S3 Figure D. B. borealis. Red= right, blue=left for cerebella, yellow=right and turquoise=left for inferior colliculi. S4 Figures. Ascending auditory tractograms. S4 Figure A1: D. delphis, left IC tracts shown in blue, right IC tracts shown in red, minimum threshold set to 1% and maximum threshold set to 30% of waytotals. Orthographic view. S4 Figure A2: D. delphis, left IC tracts shown in blue, right IC tracts shown in red, set to a more liberal threshold of minimum 0.1% and maximum 5% of waytotals. Orthographic view. S4 Figure A3: D. delphis, left IC tracts shown in blue, right IC tracts shown in red, set to a more liberal threshold of minimum 0.1% and maximum 5% of waytotals. Still 3-dimensional view. S4 Figure A4: D. delphis, left IC tracts shown in blue, right IC tracts shown in red, set to a more liberal threshold of minimum 0.1% and maximum 5% of waytotals. Rotating 3-dimensional view. S4 Figure B1: S. attenuata, left IC tracts shown in blue, right IC tracts shown in red, minimum threshold set to 1% and maximum threshold set to 30% of waytotals. Orthographic view. S4 Figure B2: S. attenuata, left IC tracts shown in blue, right IC tracts shown in red, set to a more liberal threshold of minimum 0.1% and maximum 5% of waytotals. Orthographic view. S4 Figure B3: S. attenuata, left IC tracts shown in blue, right IC tracts shown in red, set to a more liberal threshold of minimum 0.1% and maximum 5% of waytotals. Still [file pone.0323617.s001.zip › supporting_information/s7_fig_b2.png]

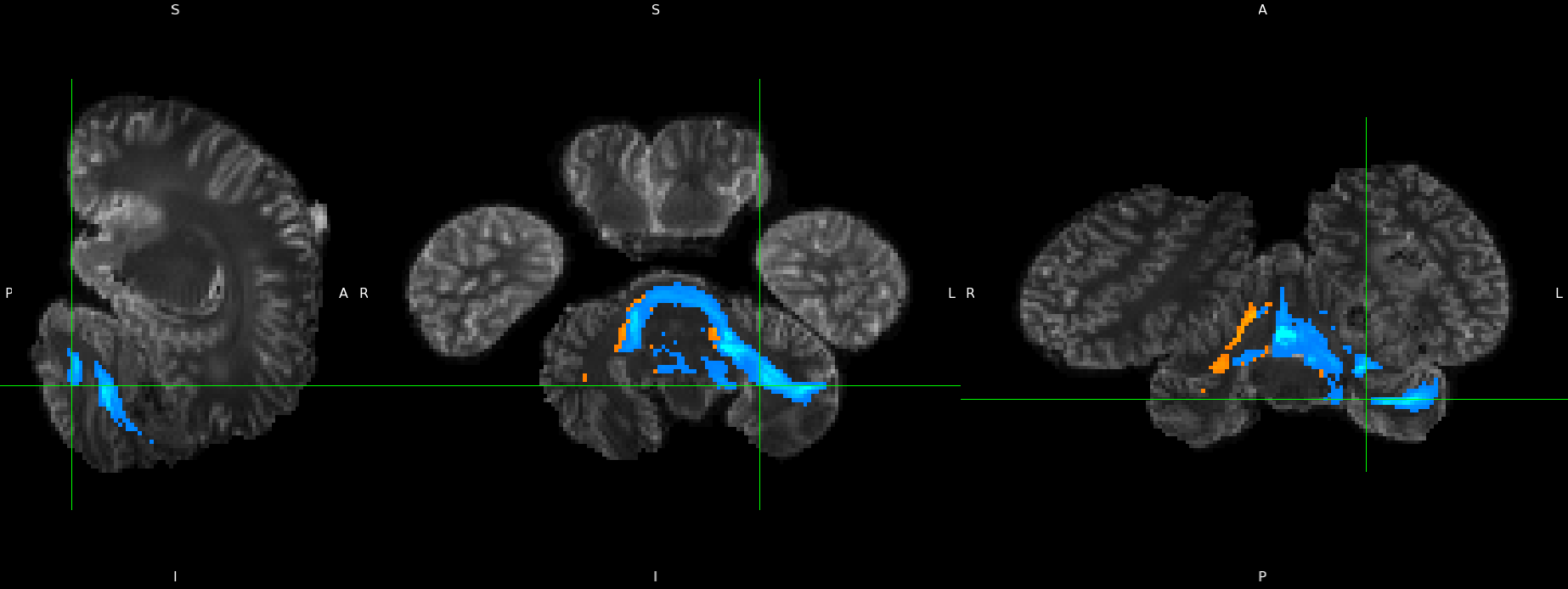

Supplement: S1 File — S1 Text. Detailed cerebellar and subcortical projection sites in IC-cerebellar traces. S2 Text. Detailed cortical projections in IC-cerebellar traces. S3 Figures. Masked regions of interest in FSLeyes. S3 Figure A. D. delphis. Red= right, blue=left for cerebella, yellow=right and turquoise=left for inferior colliculi. S3 Figure B. S. Attenuata. Red= right, blue=left for cerebella, yellow=right and turquoise=left for inferior colliculi. S3 Figure C. L. acutus. Red= right, blue=left for cerebella, yellow=right and turquoise=left for inferior colliculi. S3 Figure D. B. borealis. Red= right, blue=left for cerebella, yellow=right and turquoise=left for inferior colliculi. S4 Figures. Ascending auditory tractograms. S4 Figure A1: D. delphis, left IC tracts shown in blue, right IC tracts shown in red, minimum threshold set to 1% and maximum threshold set to 30% of waytotals. Orthographic view. S4 Figure A2: D. delphis, left IC tracts shown in blue, right IC tracts shown in red, set to a more liberal threshold of minimum 0.1% and maximum 5% of waytotals. Orthographic view. S4 Figure A3: D. delphis, left IC tracts shown in blue, right IC tracts shown in red, set to a more liberal threshold of minimum 0.1% and maximum 5% of waytotals. Still 3-dimensional view. S4 Figure A4: D. delphis, left IC tracts shown in blue, right IC tracts shown in red, set to a more liberal threshold of minimum 0.1% and maximum 5% of waytotals. Rotating 3-dimensional view. S4 Figure B1: S. attenuata, left IC tracts shown in blue, right IC tracts shown in red, minimum threshold set to 1% and maximum threshold set to 30% of waytotals. Orthographic view. S4 Figure B2: S. attenuata, left IC tracts shown in blue, right IC tracts shown in red, set to a more liberal threshold of minimum 0.1% and maximum 5% of waytotals. Orthographic view. S4 Figure B3: S. attenuata, left IC tracts shown in blue, right IC tracts shown in red, set to a more liberal threshold of minimum 0.1% and maximum 5% of waytotals. Still [file pone.0323617.s001.zip › supporting_information/s7_fig_c2.png]

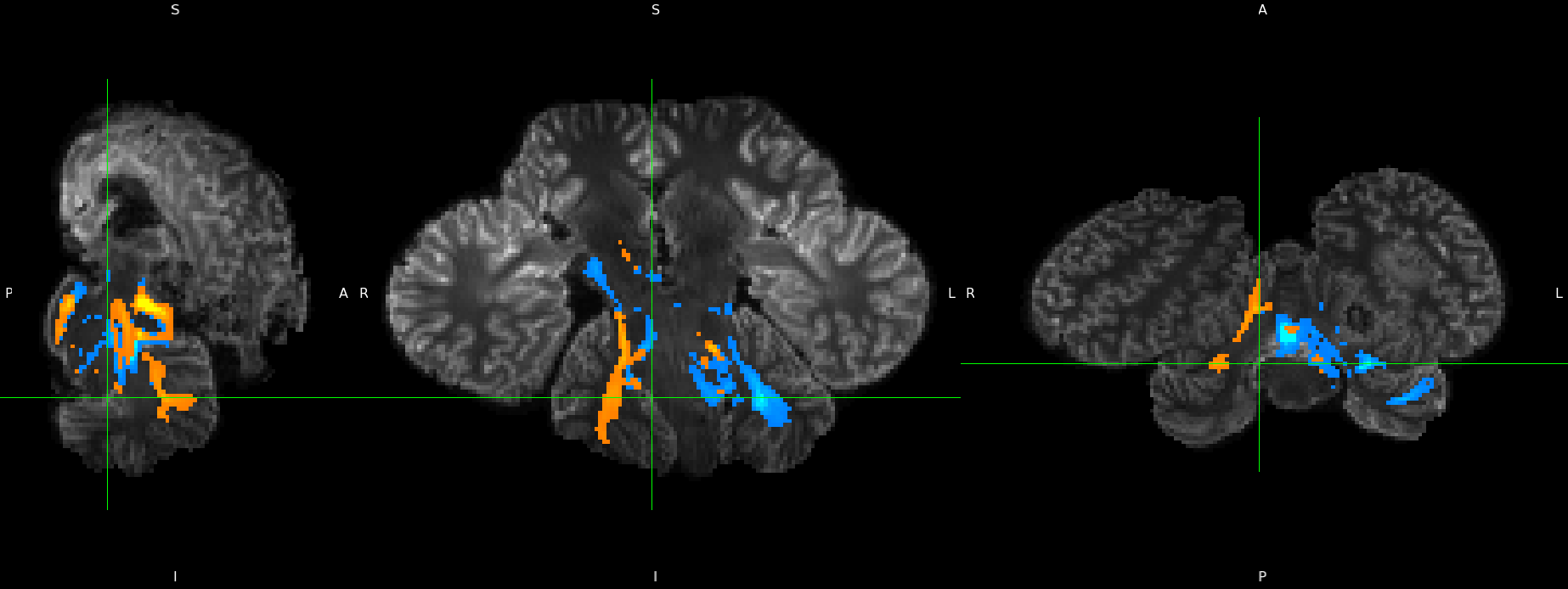

Supplement: S1 File — S1 Text. Detailed cerebellar and subcortical projection sites in IC-cerebellar traces. S2 Text. Detailed cortical projections in IC-cerebellar traces. S3 Figures. Masked regions of interest in FSLeyes. S3 Figure A. D. delphis. Red= right, blue=left for cerebella, yellow=right and turquoise=left for inferior colliculi. S3 Figure B. S. Attenuata. Red= right, blue=left for cerebella, yellow=right and turquoise=left for inferior colliculi. S3 Figure C. L. acutus. Red= right, blue=left for cerebella, yellow=right and turquoise=left for inferior colliculi. S3 Figure D. B. borealis. Red= right, blue=left for cerebella, yellow=right and turquoise=left for inferior colliculi. S4 Figures. Ascending auditory tractograms. S4 Figure A1: D. delphis, left IC tracts shown in blue, right IC tracts shown in red, minimum threshold set to 1% and maximum threshold set to 30% of waytotals. Orthographic view. S4 Figure A2: D. delphis, left IC tracts shown in blue, right IC tracts shown in red, set to a more liberal threshold of minimum 0.1% and maximum 5% of waytotals. Orthographic view. S4 Figure A3: D. delphis, left IC tracts shown in blue, right IC tracts shown in red, set to a more liberal threshold of minimum 0.1% and maximum 5% of waytotals. Still 3-dimensional view. S4 Figure A4: D. delphis, left IC tracts shown in blue, right IC tracts shown in red, set to a more liberal threshold of minimum 0.1% and maximum 5% of waytotals. Rotating 3-dimensional view. S4 Figure B1: S. attenuata, left IC tracts shown in blue, right IC tracts shown in red, minimum threshold set to 1% and maximum threshold set to 30% of waytotals. Orthographic view. S4 Figure B2: S. attenuata, left IC tracts shown in blue, right IC tracts shown in red, set to a more liberal threshold of minimum 0.1% and maximum 5% of waytotals. Orthographic view. S4 Figure B3: S. attenuata, left IC tracts shown in blue, right IC tracts shown in red, set to a more liberal threshold of minimum 0.1% and maximum 5% of waytotals. Still [file pone.0323617.s001.zip › supporting_information/s7_fig_c1.png]

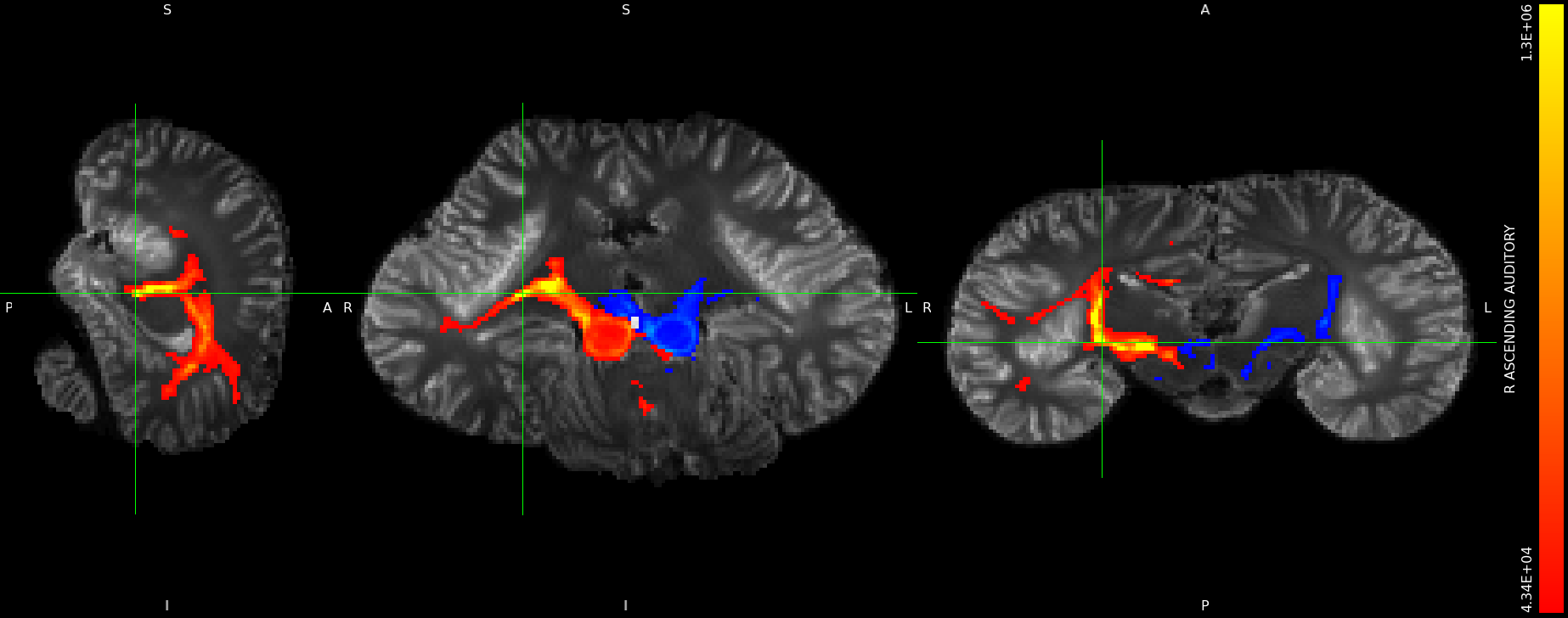

Supplement: S1 File — S1 Text. Detailed cerebellar and subcortical projection sites in IC-cerebellar traces. S2 Text. Detailed cortical projections in IC-cerebellar traces. S3 Figures. Masked regions of interest in FSLeyes. S3 Figure A. D. delphis. Red= right, blue=left for cerebella, yellow=right and turquoise=left for inferior colliculi. S3 Figure B. S. Attenuata. Red= right, blue=left for cerebella, yellow=right and turquoise=left for inferior colliculi. S3 Figure C. L. acutus. Red= right, blue=left for cerebella, yellow=right and turquoise=left for inferior colliculi. S3 Figure D. B. borealis. Red= right, blue=left for cerebella, yellow=right and turquoise=left for inferior colliculi. S4 Figures. Ascending auditory tractograms. S4 Figure A1: D. delphis, left IC tracts shown in blue, right IC tracts shown in red, minimum threshold set to 1% and maximum threshold set to 30% of waytotals. Orthographic view. S4 Figure A2: D. delphis, left IC tracts shown in blue, right IC tracts shown in red, set to a more liberal threshold of minimum 0.1% and maximum 5% of waytotals. Orthographic view. S4 Figure A3: D. delphis, left IC tracts shown in blue, right IC tracts shown in red, set to a more liberal threshold of minimum 0.1% and maximum 5% of waytotals. Still 3-dimensional view. S4 Figure A4: D. delphis, left IC tracts shown in blue, right IC tracts shown in red, set to a more liberal threshold of minimum 0.1% and maximum 5% of waytotals. Rotating 3-dimensional view. S4 Figure B1: S. attenuata, left IC tracts shown in blue, right IC tracts shown in red, minimum threshold set to 1% and maximum threshold set to 30% of waytotals. Orthographic view. S4 Figure B2: S. attenuata, left IC tracts shown in blue, right IC tracts shown in red, set to a more liberal threshold of minimum 0.1% and maximum 5% of waytotals. Orthographic view. S4 Figure B3: S. attenuata, left IC tracts shown in blue, right IC tracts shown in red, set to a more liberal threshold of minimum 0.1% and maximum 5% of waytotals. Still [file pone.0323617.s001.zip › supporting_information/s4_fig_c1.png]

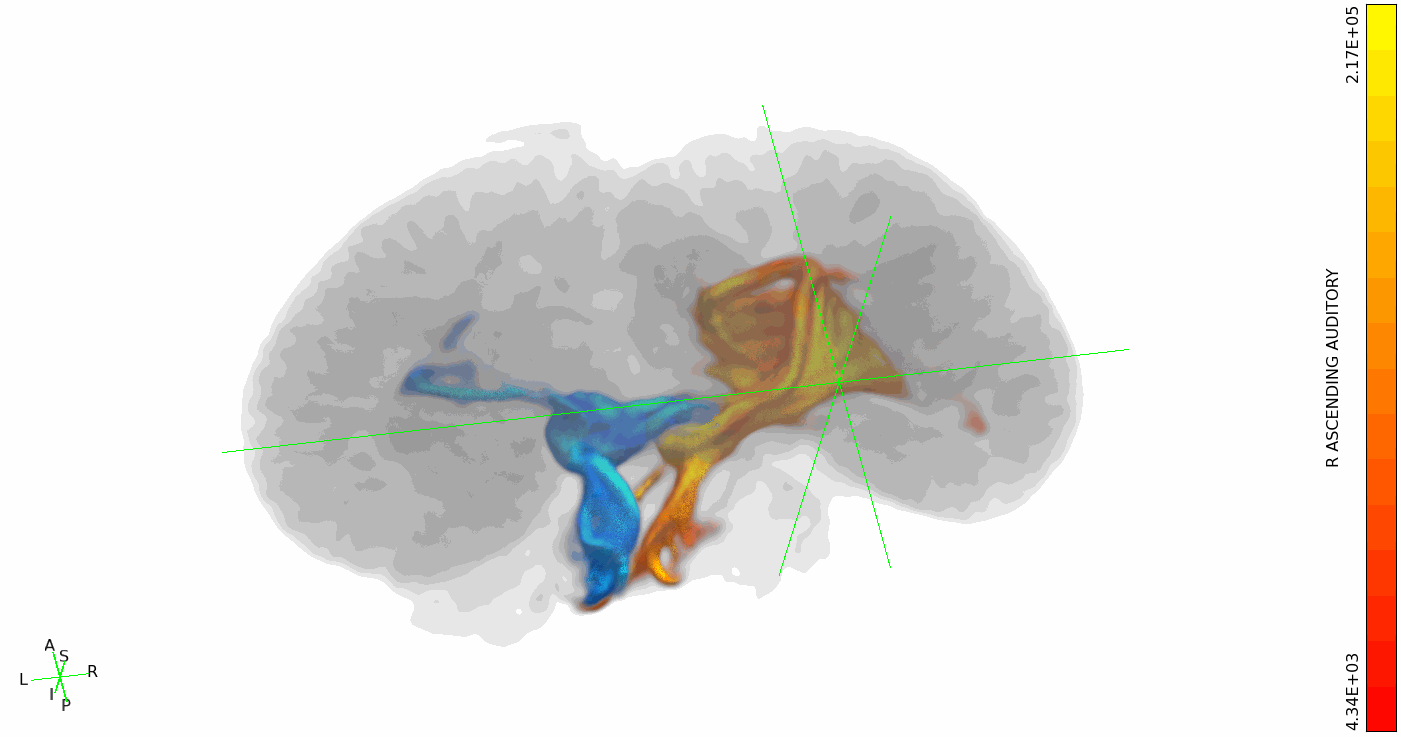

Supplement: S1 File — S1 Text. Detailed cerebellar and subcortical projection sites in IC-cerebellar traces. S2 Text. Detailed cortical projections in IC-cerebellar traces. S3 Figures. Masked regions of interest in FSLeyes. S3 Figure A. D. delphis. Red= right, blue=left for cerebella, yellow=right and turquoise=left for inferior colliculi. S3 Figure B. S. Attenuata. Red= right, blue=left for cerebella, yellow=right and turquoise=left for inferior colliculi. S3 Figure C. L. acutus. Red= right, blue=left for cerebella, yellow=right and turquoise=left for inferior colliculi. S3 Figure D. B. borealis. Red= right, blue=left for cerebella, yellow=right and turquoise=left for inferior colliculi. S4 Figures. Ascending auditory tractograms. S4 Figure A1: D. delphis, left IC tracts shown in blue, right IC tracts shown in red, minimum threshold set to 1% and maximum threshold set to 30% of waytotals. Orthographic view. S4 Figure A2: D. delphis, left IC tracts shown in blue, right IC tracts shown in red, set to a more liberal threshold of minimum 0.1% and maximum 5% of waytotals. Orthographic view. S4 Figure A3: D. delphis, left IC tracts shown in blue, right IC tracts shown in red, set to a more liberal threshold of minimum 0.1% and maximum 5% of waytotals. Still 3-dimensional view. S4 Figure A4: D. delphis, left IC tracts shown in blue, right IC tracts shown in red, set to a more liberal threshold of minimum 0.1% and maximum 5% of waytotals. Rotating 3-dimensional view. S4 Figure B1: S. attenuata, left IC tracts shown in blue, right IC tracts shown in red, minimum threshold set to 1% and maximum threshold set to 30% of waytotals. Orthographic view. S4 Figure B2: S. attenuata, left IC tracts shown in blue, right IC tracts shown in red, set to a more liberal threshold of minimum 0.1% and maximum 5% of waytotals. Orthographic view. S4 Figure B3: S. attenuata, left IC tracts shown in blue, right IC tracts shown in red, set to a more liberal threshold of minimum 0.1% and maximum 5% of waytotals. Still [file pone.0323617.s001.zip › supporting_information/s4_fig_c4.gif]

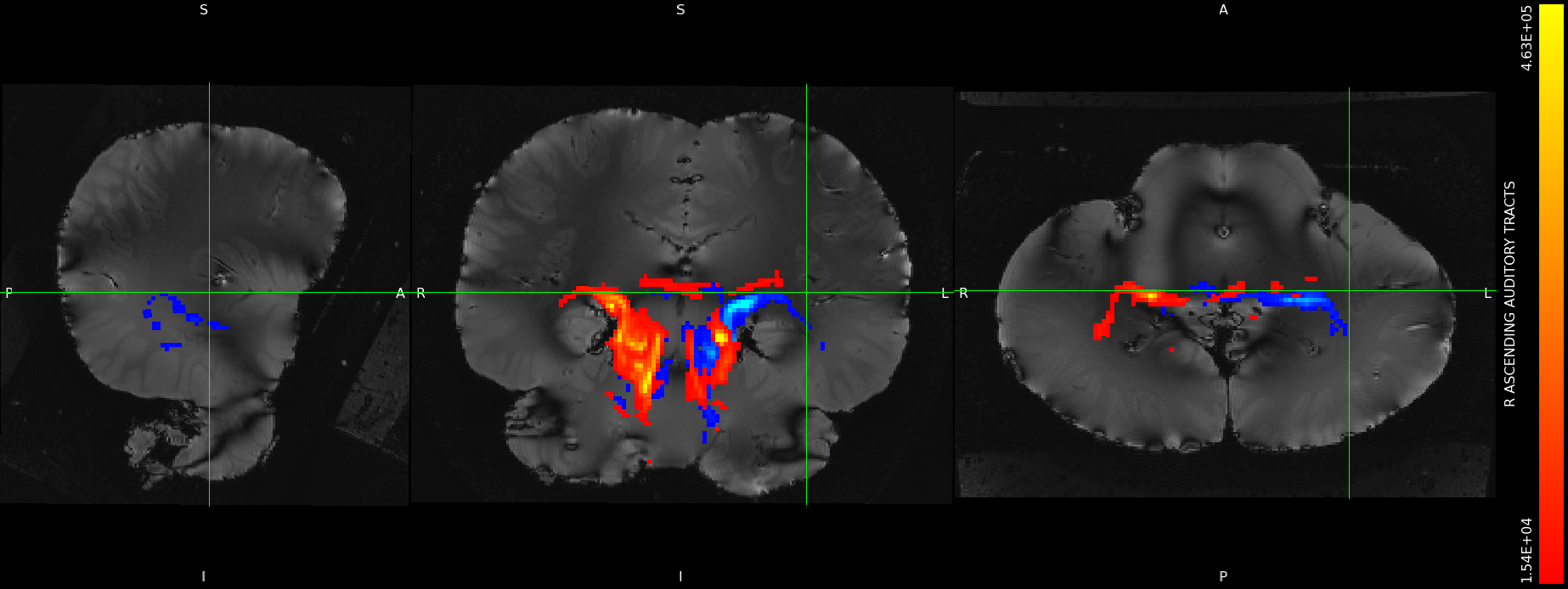

Supplement: S1 File — S1 Text. Detailed cerebellar and subcortical projection sites in IC-cerebellar traces. S2 Text. Detailed cortical projections in IC-cerebellar traces. S3 Figures. Masked regions of interest in FSLeyes. S3 Figure A. D. delphis. Red= right, blue=left for cerebella, yellow=right and turquoise=left for inferior colliculi. S3 Figure B. S. Attenuata. Red= right, blue=left for cerebella, yellow=right and turquoise=left for inferior colliculi. S3 Figure C. L. acutus. Red= right, blue=left for cerebella, yellow=right and turquoise=left for inferior colliculi. S3 Figure D. B. borealis. Red= right, blue=left for cerebella, yellow=right and turquoise=left for inferior colliculi. S4 Figures. Ascending auditory tractograms. S4 Figure A1: D. delphis, left IC tracts shown in blue, right IC tracts shown in red, minimum threshold set to 1% and maximum threshold set to 30% of waytotals. Orthographic view. S4 Figure A2: D. delphis, left IC tracts shown in blue, right IC tracts shown in red, set to a more liberal threshold of minimum 0.1% and maximum 5% of waytotals. Orthographic view. S4 Figure A3: D. delphis, left IC tracts shown in blue, right IC tracts shown in red, set to a more liberal threshold of minimum 0.1% and maximum 5% of waytotals. Still 3-dimensional view. S4 Figure A4: D. delphis, left IC tracts shown in blue, right IC tracts shown in red, set to a more liberal threshold of minimum 0.1% and maximum 5% of waytotals. Rotating 3-dimensional view. S4 Figure B1: S. attenuata, left IC tracts shown in blue, right IC tracts shown in red, minimum threshold set to 1% and maximum threshold set to 30% of waytotals. Orthographic view. S4 Figure B2: S. attenuata, left IC tracts shown in blue, right IC tracts shown in red, set to a more liberal threshold of minimum 0.1% and maximum 5% of waytotals. Orthographic view. S4 Figure B3: S. attenuata, left IC tracts shown in blue, right IC tracts shown in red, set to a more liberal threshold of minimum 0.1% and maximum 5% of waytotals. Still [file pone.0323617.s001.zip › supporting_information/s4_fig_b1.png]
