# Supplementary material for: Lateralized cerebellar connectivity differentiates auditory pathways in echolocating and non-echolocating whales
Source: PLoS One. 2025 Jun 6;20(6):e0323617. doi: 10.1371/journal.pone.0323617 (PMC12143552; doi:10.1371/journal.pone.0323617)
Supplement: Supplementary file 10 [file pone.0323617.s001.zip › supporting_information/s2_text.odt]

S2 Text. Detailed cortical projections in IC-cerebellar traces.

In D. delphis, the left IC to right cerebellar trace contained a visibly robust cortical projection to a medial-rostro-dorsal extreme in the left and right hemispheres alike, potentially in or near the anterior cingulate cortex ([S6 Figures A1-4](#_iq00q5xn6s6q)). These medial-rosto-dorsal cortical projections from the left IC to right cerebellum trace reached both hemispheres, crossing over visibly via the corpus callosum, and were accompanied by an additional rostro-dorsal projection site that was more lateral, potentially in or near putative motor and premotor regions, and was much more pronounced in the left cortex ([S6 Figure A2](#_iq00q5xn6s6q)). Notably, the cortical projections of the quantitatively weaker right IC to left cerebellum traces were primarily located in the temporal lobe of the left cortical hemisphere, with some sparse tracts appearing alongside the aforementioned left IC to right cerebellum projections in the rostro-dorsal regions of the left cortex, and with very few tracts visible in the right cortex ([S6 Figures A1-4](#_iq00q5xn6s6q)). On the whole, in D. delphis, these traces differed from ascending auditory ones in two key ways: first, temporal lobe projections were considerably weaker in left cortex and absent in right cortex, and those observed in left cortex were primarily supplied by the contralateral right IC; second, the more rostro-dorsal projections in left cortex were primarily supplied by the ipsilateral left IC and contralateral right cerebellum, whereas in the ascending auditory traces, the contralateral right IC appeared to innervate this rostro-dorsal region of left cortex more prominently (see [S4 Figures A1-4 ](#_vaxnpjfotzwc)versus [S6 Figures A1-4](#_iq00q5xn6s6q)). 

	 In contrast to D. delphis, in L. acutus, the cortical projections observed in the IC to cerebellar traces were most prominent in the right rather than left hemisphere; specifically, right IC to left cerebellar tracts were more likely to be observed in the right cortical hemisphere than left IC to right cerebellar tracts in the left cortical hemisphere ([S6 Figures C1-4](#_iq00q5xn6s6q)). Interestingly, right IC to left cerebellar traces provided the vast majority of cortical projections in the left hemisphere; left IC to right cerebellar traces showed very few left-hemispheric cortical projection sites ([S6 Figures C1-4](#_iq00q5xn6s6q)). Both right IC to left cerebellar and left IC to right cerebellar traces projected to similar cortical areas as those implicated in ascending auditory traces in L. acutus, though the overlap was most minimal in the caudal-most projection sites reached by ascending auditory traces (compare [S4 Figure C2](#_vaxnpjfotzwc) with [S6 Figure C2](#_iq00q5xn6s6q)). IC to cerebellar traces in L. acutus differed from ascending auditory traces most notably in the extent of their dorsal-rostral projections; in the right hemisphere and left hemispheres, respectively, left IC to right cerebellar and right IC to left cerebellar traces were observed reaching a dorso-rostral extreme that was not reached by plain ascending auditory traces (most apparent in [S6 Figure C4](#_iq00q5xn6s6q)).

	Out of all of the dolphins, S. attenuata exhibited the weakest cortical projections in IC to cerebellar traces. As in the case of L. acutus, cortical projections for these traces overlapped the least with those in ascending auditory traces in the caudal and ventral cortical extremes, and were more likely to appear in dorsal extremes than their ascending auditory counterparts (compare [S4 Figure B2 ](#_vaxnpjfotzwc)with [S6 Figure B2](#_iq00q5xn6s6q)). On the other hand, in S. attenuata, a more unique dorso-caudal left-cortical projection site was observed in both right IC to left cerebellar traces and left IC to right cerebellar ones ([S6 Figure B2](#_iq00q5xn6s6q)). Mirroring the case of D. delphis, both right IC to left cerebellar traces and left IC to right cerebellar traces were more likely to innervate the left cortex than the right, though in S. attenuata this tendency was less pronounced, as some right IC-left cerebellar projections to right cortex appeared at the more liberal threshold ([S6 Figure B2](#_iq00q5xn6s6q)).

As stated in the main text, cortical projections from IC to cerebellar traces were incredibly sparse in B. borealis ([S6 Figures D3-4](#_iq00q5xn6s6q)). Some weak tracts from left IC to right cerebellar traces were observed in the left thalamus at the more conservative threshold ([S6 Figure D1](#_iq00q5xn6s6q)), and the more liberal threshold revealed minor right IC-left cerebellar tracts that reached right dorso-rostral cortex ([S6 Figure D2](#_iq00q5xn6s6q)). No other cortical projections were robust enough to remain visible after thresholds were applied.
