# Supplementary material for: Lateralized cerebellar connectivity differentiates auditory pathways in echolocating and non-echolocating whales
Source: PLoS One. 2025 Jun 6;20(6):e0323617. doi: 10.1371/journal.pone.0323617 (PMC12143552; doi:10.1371/journal.pone.0323617)
Supplement: Supplementary file 10 [file pone.0323617.s001.zip › supporting_information/s1_text.odt]

S1 Text. Detailed cerebellar and subcortical projection sites in IC-cerebellar traces.

In left IC to right cerebellar traces in D. delphis, the strongest cerebellar projections were at the rostral extreme of the cerebellum, particularly in its connections with brainstem nuclei and the pons ([S](#_iq00q5xn6s6q)[6 Figure ](#_iq00q5xn6s6q)[A1-2](#_iq00q5xn6s6q)). The most rostral and dorsal of these tracts passed from a rostro-dorsal region of left cortex to the right cerebellum via the substantia nigra and crus cerebri, just dorsal to the pons ([S6 Figure A1-2](#_iq00q5xn6s6q)). From here, one branch of the trace ran ventrally to join with a larger clump of tracts traversing the pons, while another remained dorso-rostral, connecting with a dorsal-rostral extreme of the cerebellum, likely a rostral extreme of lobule IX ([S6 Figure A1-2](#_iq00q5xn6s6q)). Toward its ventral extreme, the robust pontine fiber bundle branched off into three directions. One branch extended contralaterally and caudally to the left-side trapezoid body, cerebellar peduncle, and lateral lemniscus ([S6 Figure A2](#_iq00q5xn6s6q)); a second branch remained rostral but moved laterally and ventrally via a cerebellar peduncle to putative lobule IX ([S7 Figure A1](#_3zpl9zue7xys)); a third branch forked off from the second after passing through the cerebellar peduncle, projecting more caudally to putative lobule VIIb, a projection mirrored in the right IC-left cerebellar trace ([S7 Figures A1-2](#_3zpl9zue7xys)). Near the rostral-caudal fork between the latter two paths, a small projection to putative lobule VIIIa can be seen ([S7 Figures A1-2](#_3zpl9zue7xys)). Lobule X, also fairly small in cetacea, also appears to receive bilateral projections from both left and right IC in both cerebella ([S7 Figure A1](#_3zpl9zue7xys)).

	In contrast to the left IC to right cerebellar traces, the right IC to left cerebellar trace in D. delphis did not include a clear path from cortex to cerebellum through the rostral midbrain. There were robust tracts in the ventral pons, and similarly to the left IC to right cerebellar traces, these forked into multiple pathways, including one that passed to right-side auditory structures like the trapezoid body and lateral lemniscus, and another to a rostral portion of left-cerebellar lobule IX via the peduncles ([S7 Figures A1-2](#_3zpl9zue7xys)). The caudal-reaching fork in this trace had a projection that reached a more caudal-lateral extreme than was observed in the left IC-right cerebellar trace, likely near Crus I and II ([S7 Figure A2](#_3zpl9zue7xys)). Additionally, the right IC to left cerebellar trace also exhibited a more robust path to ventral lobule IX than any observed in left IC to right cerebellar traces ([S7 Figure A2](#_3zpl9zue7xys)). Rostral to these sites, there was a rostral and medial projection to putative lobule VIIb, similar to the projection to this lobule observed in the left IC to right cerebellar trace ([S7 Figures A1-2](#_3zpl9zue7xys)). Notably, the right IC to left cerebellar tracts were more bilaterally robust in the brainstem auditory regions like the lateral lemniscus and trapezoid body, and additionally made it further down the brainstem, than left IC to right cerebellar tracts ([S7 Figures A1-2](#_3zpl9zue7xys)). 

	Similarly to D. delphis, the left IC-right cerebellar traces in S. attenuata exhibited multiple distinct pathways at the rostral extreme of the pons, with three apparently transverse bundles at its dorsal extreme, its ventral extreme, and a more medial dorsal level, possibly representing pathways through the superior, inferior, and medial cerebellar peduncles, respectively ([S7 Figure B1](#_3zpl9zue7xys)). In contrast to D. delphis, all three of these, including the dorsal-most of the bundles, was also observed in the right IC to left cerebellar traces, and direct cortical connections through rostral diencephalon and mesencephalon were not observed in either ([S7 Figures B1-2](#_3zpl9zue7xys)), though more caudal ones from IC to putative MGN were ([S6 Figures B1-2](#_iq00q5xn6s6q)). As in the other dolphins, the majority of the pontine projections for both traces appeared to target putative lobules VIIIb and IX ([S7 Figures B1-2](#_3zpl9zue7xys)). Also, as observed in both other dolphin specimens, the right IC-left cerebellar traces in S. attenuata contained a far-lateral caudal projection, likely to putative left Crus I and II, not mirrored in the right cerebellum and left IC-right cerebellar traces ([S7 Figures B1-2](#_3zpl9zue7xys)). In contrast to the other dolphins, both traces displayed robust pathways throughout the vermis ([Fig 7b](#_6xd3uru0cdzf)). Furthermore, compared to other specimens, the tracts in the brainstem regions between pons and cerebellum in S. attenuata tended to be more bilaterally robust for both left IC to right cerebellar and right IC to left cerebellar traces ([S6 Figures B1-2](#_iq00q5xn6s6q)). Finally, more clearly than in any other specimens, both IC to cerebellar traces in S. attenuata displayed prominent dorsal-ventral projections through the mylencephalon, possibly traversing the inferior cerebellar peduncle, that proceeded ventrally and laterally from the floor of ventricle IV on the caudal brainstem, potentially contacting the facial colliculi situated here, to the caudal border of the inferior olives and eventually the rostral-most portion of the spinal cord ([Fig 7b](#_6xd3uru0cdzf)). Interestingly, the left IC to right cerebellum trace, but not the right IC to left cerebellum trace, produced a second, similarly continuous and robust projection between the floor of ventricle IV and the caudal edge of the inferior olives, medial and slightly rostral to the bilateral parallel projections described above, closer to the facial motor nucleus ([Fig 7b](#_6xd3uru0cdzf)). 

In L. acutus, both IC to cerebellar traces had their most concentrated paths in the transverse fibers of the pons most rostrally, and in all three of the cerebellar peduncles most caudally ([S7 Figures C1-2](#_3zpl9zue7xys)). In left IC to right cerebellar traces, the most prominent of these peduncular paths appeared to target the juncture of putative lobules VIIIa and VIIIb, a pattern mirrored in the right IC to left cerebellar traces of L acutus ([S7 Figures C1-2](#_3zpl9zue7xys)). We also observed a unique feature of left IC to right cerebellar traces that was not observed in their contralateral counterparts: a second projection more medial, caudal, and dorsal than the first, fairly close to vermis, likely targeting putative lobule VI ([S7 Figure C1](#_3zpl9zue7xys)), a projection that was only observed here and in the left cerebellum of B. borealis ([S6 Figure D1](#_iq00q5xn6s6q)).

	The right IC to left cerebellar traces in L. acutus included a unique feature also observed in those of D. delphis: a more caudal-lateral projection than any observed in the left IC to right cerebellar traces, likely to putative Crus I or Crus II ([S7 Figure C2](#_3zpl9zue7xys)). Further rostral to this, the most pronounced projection to left cerebellum was found in the putative conjunction of lobules VIIIa and VIIIb, connected to the auditory brainstem regions caudal to the ventral pons via the peduncles as was observed in the left IC to right cerebellar traces of L. acutus ([S7 Figures C1-2](#_3zpl9zue7xys)). Notably, right IC to left cerebellar tracts were more bilaterally robust in these brainstem regions caudal to the ventral pons than left IC to right cerebellum tracts, contacting the right cerebellar peduncles in addition to the left ones ([S7 Figure C2](#_3zpl9zue7xys)). Additionally, the right IC to left cerebellar traces contained a secondary projection not mirrored in the left IC to right cerebellar traces. This projection was almost as robust as the one to lobules VIIIa and VIIIb, and was found more rostrally in the coronal plane as the most robust transverse pontine fiber crossover, likely targeting lobule IX ([S7 Figure C2](#_3zpl9zue7xys)). Finally, right IC to left cerebellar traces in L. acutus included a rather robust set of tracts through the vermis, particularly vermal lobules IV, VI, and VIIat, that were not observed in the left IC to right cerebellum traces of this specimen ([Fig 7c](#_6xd3uru0cdzf)). 

In B. borealis, right IC to left cerebellar traces exhibited four main projections: one to a far-rostral point in putative lobule IX ([S6 Figure D1](#_iq00q5xn6s6q)); a second that forked from the far-rostral projection to a lateral extreme that was slightly more caudal but still rostral of the midpoint of the rostral-caudal axis, putatively identified as a lobule VIIb projection ([S6 Figure D1](#_iq00q5xn6s6q)); a third that projected caudally, ventrally, and laterally relative to the first two towards the border of lobules VIIb and VIIIa ([S6 Figures D1-2](#_iq00q5xn6s6q)); and finally, a fourth far-caudal and far-dorsal projection, directly lateral to the vermis, that forked into two projections that approached putative lobule VI and crus I ([S7 Figure D2](#_3zpl9zue7xys)). Finally, right IC to left cerebellum traces contained more paths through the pons than left IC to right cerebellum traces ([S7 Figure D2](#_3zpl9zue7xys)). 

	By contrast, left IC to right cerebellar traces in B. borealis featured only two pronounced projections, and these mirrored the first and third above-listed ones observed in right IC to left cerebellar traces: one to a lateral, far-rostral, and mid-ventral location, putatively identified as lobule IX ([S6 Figures D3-4](#_iq00q5xn6s6q)), and the other to a lateral far-caudal and ventral site around the junctures of lobules VIIb and VIIIa ([S7 Figure D1](#_3zpl9zue7xys), [S6 Figure D2](#_iq00q5xn6s6q)). The left IC to right cerebellum trace also contained a minor projection to left cerebellum’s putative lobule VIIb, directly overlapping with the right IC to left cerebellar trace’s projection to this site ([S6 Figure D1](#_iq00q5xn6s6q)).
